# Asymmetric Synthesis of Spirooxindoles via Nucleophilic Epoxidation Promoted by Bifunctional Organocatalysts

**DOI:** 10.3390/molecules23020438

**Published:** 2018-02-16

**Authors:** Martina Miceli, Andrea Mazziotta, Chiara Palumbo, Elia Roma, Eleonora Tosi, Giovanna Longhi, Sergio Abbate, Paolo Lupattelli, Giuseppe Mazzeo, Tecla Gasperi

**Affiliations:** 1 Dipartimento di Scienze- Sezione di Nanoscienze e Nanotecnologie, Università degli Studi di Roma Tre, V.le G. Marconi 446, I-00146 Rome, Italy; martina.miceli@icloud.com (M.M.); adma@kemi.dtu.dk (A.M.); ch.palumbo@hotmail.it (C.P.); elia.roma@uniroma3.it (E.R.); eleonoratosi93@gmail.com (E.T.); 2 Dipartimento di Medicina Molecolare e Traslazionale (DMMT), Università di Brescia, viale Europa 11, 25123 Brescia, Italy; giovanna.longhi@unibs.it (G.L.); sergio.abbate@unibs.it (S.A.); giuseppe.mazzeo@unibs.it (G.M.); 3 Dipartimento di Scienze, Università degli Studi della Basilicata, via dell′Ateneo Lucano 10, I-85100 Potenza, Italy; paolo.lupattelli@unibas.it

**Keywords:** epoxidation, organocatalysis, epoxyoxindole, alkylidenoxindoles, H-bond network, non-covalent catalysis, chiroptical properties

## Abstract

Taking into account the postulated reaction mechanism for the organocatalytic epoxidation of electron-poor olefins developed by our laboratory, we have investigated the key factors able to positively influence the H-bond network installed inside the substrate/catalyst/oxidizing agent. With this aim, we have: (i) tested a few catalysts displaying various effects that noticeably differ in terms of steric hindrance and electron demand; (ii) employed α-alkylidene oxindoles decorated with different substituents on the aromatic ring (**11a**–**g**), the exocylic double bond (**11h**–**l**), and the amide moiety (**11m**–**v**). The observed results suggest that the modification of the electron-withdrawing group (EWG) weakly conditions the overall outcomes, and conversely a strong influence is unambiguously ascribable to either the *N*-protected or *N*-unprotected lactam framework. Specifically, when the NH free substrates (**11m**–**u**) are employed, an inversion of the stereochemical control is observed, while the introduction of a Boc protecting group affords the desired product **12v** in excellent enantioselectivity (97:3 *er*).

## 1. Introduction

“If carbonyl compounds have been said to be virtually the backbone of organic synthesis, the epoxides correspond to at least one of the main muscles” (Prof. D. Seebach) [1]. As an irreplaceable muscle, over the years, chiral epoxides have proved to be highly valuable intermediates and outstanding building blocks in fine organic synthesis. Indeed, the oxirane ring easily undergoes several stereoselective chemical transformations into a variety of multi-functionalized compounds that directly led to advanced precursors of both natural products and biologically active molecules [2,3,4,5,6,7]. Among them, spiro-epoxyoxindoles, featured with a spiro-carbon and unstable oxirane motif, have piqued the interest of a growing number of research groups [8,9,10]. Such striking allure is mainly due not only to their recognised medicinal effectiveness [11,12,13,14,15,16,17], but also to their role providing direct access to architecturally complex heterocycles [18,19,20,21,22]. Although various fascinating approaches have been developed for their preparation, only few are efficient and effective strategies to achieve this notable framework with high optical purity. For instance, since the seminal work of Brière and Metzner [23], stereoselective Darzen-type processes have been the object of several investigations. In 2014 Xiao et al. succeeded in the synthesis of epoxyoxindoles from isatines [8]. Likewise, Feng and co-workers investigated a Co(acac)_2_-*N,N*′-dioxide-catalyzed Darzens reaction to afford benzyl-substituted *trans*-spirooxirane-oxindoles [10], while, more recently, Wong has focused his efforts on the use of (R)-BINOL and Ti(iOPr)_4_ as catalysts reaching a high level of stereoselectivity involving chiral sulfur ylides in situ generated from camphor-derived sulfonium salts [24]. Conversely, less explored have been the stereoselective epoxidation of α-ylidenoxindoles with peroxide, which even though these furnished the titled products with excellent diastereomeric ratios, provided the corresponding spiroepoxides only with moderate to good enantioselectivity [25,26,27].

**Figure 1:**
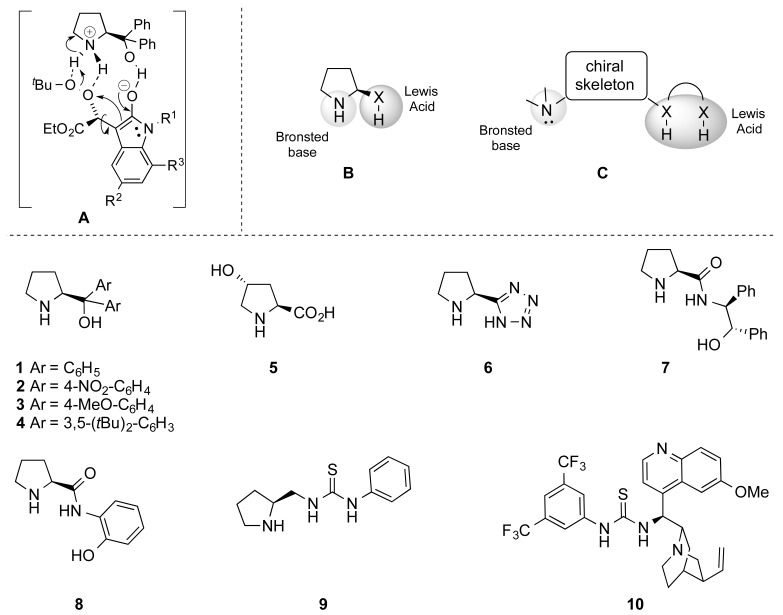
Postulated transition state (**A**) of the previously optimized organocatalytic epoxidation that highlights the substrate/catalyst/oxidant H-bond network. Hypothesized catalyst structures (**B**,**C**). Organocatalysts examined in this study (**1**–**10**).

In the past few years, we have been engaged in the development of the nucleophilic epoxidation of α-ylidenoxindoles as well as in the improvement of the corresponding organocatalytic version [25,26], which mostly relies on the catalyst’s ability to install in the transition state a strong network with substrate and oxidizing agent (Figure 1A). Pursuing our research and prompted by the continuous interest concerning spiroepoxy derivatives, we envisaged enhancing the previously reported procedure by introducing further additional groups capable of installing more H-bonds inside the substrate/catalyst/oxidant system by modifying both the substrate and the catalyst. Specifically, since the first step of our nucleophilic epoxidation involved an *oxa*-Michael addition and, taking into account the analogous activation process of the enoyl-CoA hydratase in fatty acid metabolism, we envisaged the possibility of employing a catalyst bearing two hydrogen donor groups that should increase the α,β-unsaturated bond electrophilicity and make stronger the H-bond network in the postulated transition state **A**.

## 2. Results and Discussion

### 2.1. Evaluation of the Catalyst Modification

Initially, considering the efficiency of the α, α-diarylprolinol **1**, the catalyst scope was enlarged to various compounds in which: (i) either the aromatic rings or the pyrrolidine framework were decorated with substituents different in terms of hindrance- and electron demand (**2**–**5**, Figure 1); (ii) the Brønsted basic site was preserved, whereas the Lewis acidic site was changed to a tetrazolic ring (**6**, Figure 1); (iii) the Brønsted basic site was preserved, while two hydrogen bond donors were introduced (**7** and **8**, e.g., an hydroxyl group and an amide moiety, Figure 1); (iv) the Brønsted basic site was preserved, and linked to a thiourea through a methylene (**9**, Figure 1); (v) the chiral skeleton was changed to an *epi*-quinine, in which the basic site is a tertiary amine and the Lewis acid group is a thiourea (Soós catalyst **10**, Figure 1).

The nucleophilic epoxidation reaction was initially performed using the alkyliden oxindole **11a** as model substrate, testing catalysts **2**–**10** and employing previously optimized conditions [25] (i.e., TBHP as oxidizing agent, hexane as solvent, and room temperature). The observed outcomes were compared with the simplest α,α-diphenyl prolinol **1** (Table 1). The presence of an electron withdrawing group (Ar = 4-NO_2_-C_6_H_4,_ catalyst **2**, entry 2) on the aromatic moiety dramatically reduce the enantioselectivity as well as the efficiency in terms of time and yield, even though the diastereomeric ratio was quite favourable to the epoxide *trans*-**12**. Conversely, the introduction of an electron donating moiety (Ar = 4-OMe-C_6_H_4,_ catalyst **3**, entry 3), dramatically reduced the diastereoselectivity, while the enantiomeric ratio was almost comparable to the reference catalyst. Additionally, the more hindered catalyst **4** (entry 4) slightly favoured the formation of the *cis* isomer (25:75) without any significant enhancement in the enantioselectivity, which was completely smoothed over when a second stereogenic centre was introduced on the five-membered ring (entry 5).

The tetrazolic catalyst **6** (entry 6), featured with an NH function instead of the free hydroxyl moiety, furnished an interesting high diastereo- (79:21) and enantioselectivity (92:8), but a pronounced decrease of reactivity was observed in terms of reaction time (240 h) and yield (4%). The use of other amidic catalysts was subsequently evaluated: pyrrolidine derivative **7** and **8** (Figure 1), in which the OH function is sustained by a second NH function, and thioureas **9** and **10** (Figure 1), in which the OH function is substituted by an NH moiety. Unfortunately, no substantial improvement was observed and only lower enantioselectivity was detected (entries **7**–**9**). A distinctive issue has to be addressed when compound **10** was employed. Indeed, although the Soós thiourea (**10**) furnished the desired products with an optical purity comparable to the model reaction, the low overall yield (36%) and a reaction time that was too long (144 h) made catalyst **10** unsuitable for further investigations.

Since the Since the just disclosed results are quite difficult to rationalize and clearly highlight that the overall domino reaction is not positively affected by either the substitution of the hydroxyl with other functions, or a change of the catalyst’s scaffold, or the introduction of an additional H-bonding site, we decided to pursue our investigations employing diarylprolinol **1**, the best catalyst so far.

Specifically, the nucleophilic epoxidation was performed on the *N*-methyl carboxylate ylideneoxindoles **11b**–**g** (entries 11–16) that always furnished the easily separable diastereomers *trans*-**12b**–**g** and *cis*-**13b**–**g**, in good to excellent yields and with notable enantioselectivity (up to 93:7 *er*) in favour of the enantiomers (*2R′,3R*′) and (*2S′,3R*′), respectively. Noteworthy, the epoxidation of compounds endowed with two chlorine atoms (entry 13), led to the highest enantioselectivities for the *trans* product (93:7 *er*) together with the highest optical purity for the *cis* isomer too (77:23 *er*). In addition, in few cases (entries 11–13, 15), an inversion of the diastereomeric ratio was observed, in favour of the products *cis-***13**. Such an effect could be mainly ascribable to some supplementary interactions between the substituent and the catalyst able to stabilize the transition state that precedes the *cis* isomer formation. Furthermore, the steric hindrance as well as the electronic demand on the aromatic ring due to either a methoxy group (entry 14) or to a naphthyl-derivative (entry 16) do not strongly affect the overall reactivity.

### 2.2. Evaluation of the Substrate Scope

In order to explore the effect of the EWG on the developed catalytic system, the reaction was subsequently performed on α-ylidenoxindole where the carboxylate had been replaced by the more hindered phosphonic moiety, which should contribute mostly to an inductive effect (-I) When phosphonate α-ylidenoxindoles **11h**–**l** (Table 2) were employed as substrates in the optimised organocatalytic epoxidation, a moderate to good enantioselectivity was observed for the *trans* isomers (**12h**–**l**, up to 80:20 *er*); meanwhile, the steric hindrance due to the larger electronwithdrawing group [PO(OEt)_2_] enhanced the formation of the *cis* isomers (**13h**–**l**), which, as already observed with carboxylic derivatives, became the major diastereomer when a halide (**11l**) was introduced in position 5 on the aromatic ring.

The given stereochemical outcomes are assigned upon a thorough comparison of the reported experiments, analytical data, and a quantum mechanical *ab initio* calculation of chiroptical properties. Specifically, the major diastereomers were identified as the *trans* products by comparing the spectroscopic analyses of each isolated compound with the corresponding data already reported in literature [26], while the (2′*S*,3′*S*) configuration was assigned to the major enantiomer of *trans*-**12h** (see the stereochemistry analysis section below).

The forehead results clearly suggest that the tested substituents on the exocyclic double bond do not dramatically influence the reagent approach to the electrophilic center (C_β_) which is preferentially driven toward the less-hindered *Re*-face of the double bond, although the steric hindrance noticeably affects the stereoinduction.

Subsequently, the substrate scope was enlarged by evaluating α-ylidenoxindoles **11m**–**u** variously decorated on the aromatic ring and featured a *N*-unprotected amide, which should install a further H-bond between the substrate and the catalyst-oxidant complex (Table 3).

Comparing the data depicted in Table 3 with the outcomes previously observed [25], it is undeniable that the reaction was successfully in term of overall yields (up to 99%) without any loss concerning the diastereomeric ratios (up to 78:22 dr), whereas only a slight decrease in the enantioselectivity was detected. Nevertheless, a quite surprising result was the outgrowth deriving from the iodine derivative **11p** (entry 4, Table 3), which furnished an inverted diastereomeric ratio (78:22 in favour of the *trans* isomer), lower reactivity (170h vs. 24h), and lower optical purity (68:32 vs. 80:20) compared to the corresponding methylated molecule (entry 11, Table 1).

Such observations, taking into account the stereoselectivity previously detected with phosphonate derivatives **11i**–**k**, which were decorated with different *N*-substituents (entries 2–4, Table 2), suggest a special role of the free NH function in the H-bond network existing among substrate/catalyst/oxidant, a hypothesis which was validated (i) by analysing the configuration of the newly formed stereogenic centres; as well as (ii) by introducing the *tert*-butyl carbonyl moiety as nitrogen-protecting group that should furnish a further coordination site in the epoxidation transition state.

**Scheme 1:**
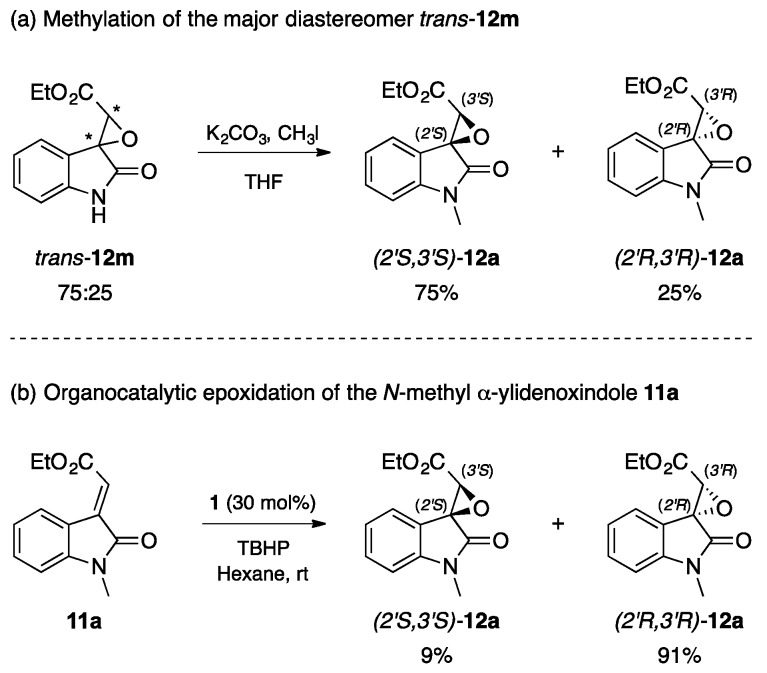
Comparison between *N*-methyl spiroepoxyoxindoles furnished (**a**) by the methylation of the major diastereomer *trans*-**12m**; and (**b**) by the organocatalytic epoxidation of the *N*-methyl α-ylidenoxindole **11a**.

In order to understand how the nucleophilic attack of the oxidizing agent toward the electrophilic centre (C_β_) on the electron poor olefins **11m**–**u** is stereochemically driven, the experiment depicted in Scheme 1a was performed. Specifically, the *N*-methylation of the major diastereomer *trans–***12m** led to a clean product that, upon spectroscopic and chiral HPLC analysis, resulted to be the enantiomer (2′*S*,3′*S*)-**12a**, i.e., the minor enantiomer obtained during the direct organocatalytic epoxidation of the model substrate **11a** (entry 1, Table 1).

Therefore, the first attack on **11m** occurs at the top face of the double bond, i.e., oppositely to what was observed when *N*-methyl α-ylideneoxindoles **11a**–**l** were employed.

**Scheme 2:**
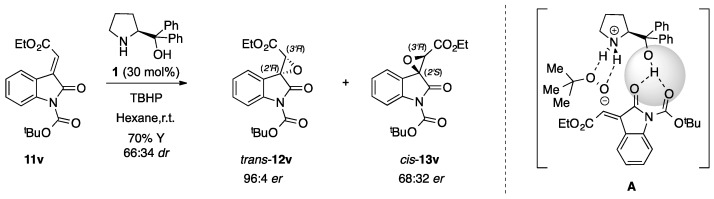
Organocatalytic epoxidation reaction carried on *N*-Boc protected α-ylidenoxindole **11v [30]** using 2.0 equivalent of oxidating agent (TBHP).

Additionally, to our delight, the epoxidation reaction carried on the *N*-Boc protected α-ylideneoxindole **11v** successfully provided the desired spiroepoxide *trans*-**12v** and *cis*-**13v** not only with good diastereomeric excess (66:34 *dr*), as previously detected, but especially with excellent enantioselectivity (97:3 *er*) in the case of the major isomer *trans*-**12v**. Actually, in the first trial complete conversion was accomplished only after 144 h with a quite poor yield of 36%, an outcome that was remarkably improved (70% of total yield, 24h of reaction time) by using a higher excess of the oxidizing agent (2.0 equiv. instead of 1.2 equiv.) without any loss in stereoselectivity (66:34 *dr,* 96:4 *er* for the major diastereomer *trans*-**12v**) (Scheme 2).

### 2.3. Stereochemistry Analysis

To better investigate the stereochemistry of compounds *trans*-**12h** and *cis*-**13h,** we employed chiroptical spectroscopies. In particular, we performed electronic and vibrational circular dichroism (ECD and VCD) and optical rotatory dispersion (ORD) measurements with subsequent analysis by density functional theory (DFT) and time-dependent DFT (TDDFT) calculations [31,32,33,34,35,36,37].

**Figure 2:**
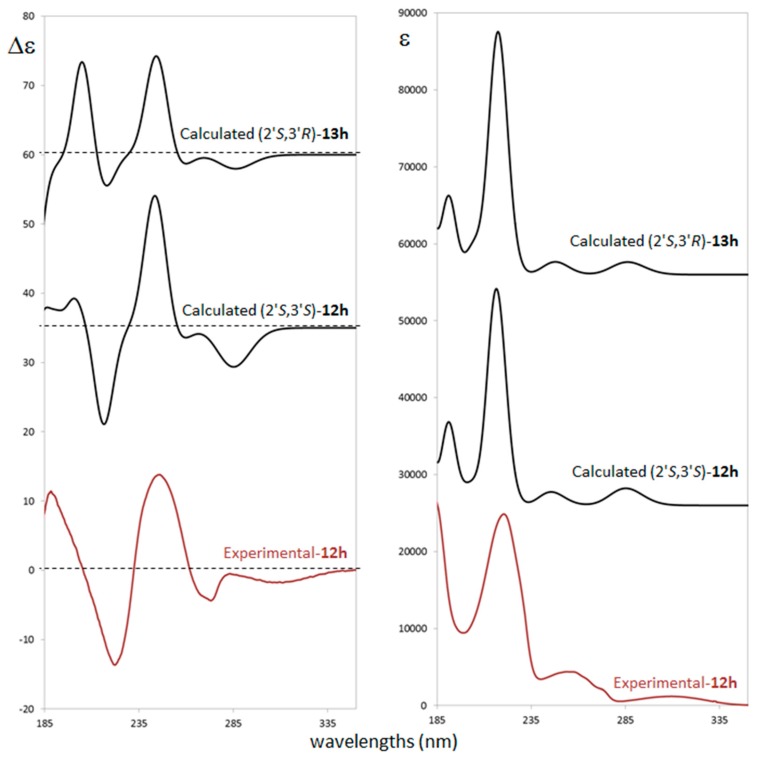
Comparison of experimental and computed electronic circular dichroism (ECD) (**left**) and ultraviolet (UV) (**right**) spectra for the different ACs for compounds *trans*-**12h** (experimental and calculated) and *cis*-**13h** (calculated).

ECD measurement was performed in acetonitrile solution (see experimental details in supporting material) in the range of 400–180 nm. The ECD spectrum exhibits a weak negative band at ca. 310 nm, a splitted negative band at ca. 273 nm, and a sequence of intense positive, negative and positive bands at 250, 224 and about 190 nm, respectively. The negative band at 273 nm and the positive at 250 nm are both allied to the ultraviolet (UV) band centered at 260 nm which shows multiple features (Figure 2). ECD/UV calculation was performed at TDDFT/CAM-B3LYP/TZVP level on the previously optimized most populated conformers at DFT/B3LYP/TZVP level with polarizable continuum model (PCM) [38] approximation (see Figure SM-2 for details) of both the (2′*S*,3′*S*) and the (2′*S*,3′*R*) diastereomers. In Figure 2 a comparison between experimental ECD and UV spectra with (2′*S*,3′*S*) and (2′*S*,3′*R*) calculated ones is presented.

Despite small differences on relative intensities of the negative and the positive bands at 224 and 190 nm respectively, the calculated spectra of both diastereomers do not clearly discriminate about 3′ carbon AC. On the other hand, unambiguous assignment is provided for 2′ carbon as *S*. Safe assignment is also supported by considering that all single conformers ECD spectra do not differ each other (Figure SM-3) showing that the calculation is also quite insensitive to populations and energy calculation accuracy. All conformers differ by relative orientation of phosphate and ethoxy moieties (see Figure SM-1) while oxindole chromophore has quite a rigid structure leading to similar ECD profiles which are driven by the *S* configuration of 2′ carbon.

**Figure 3:**
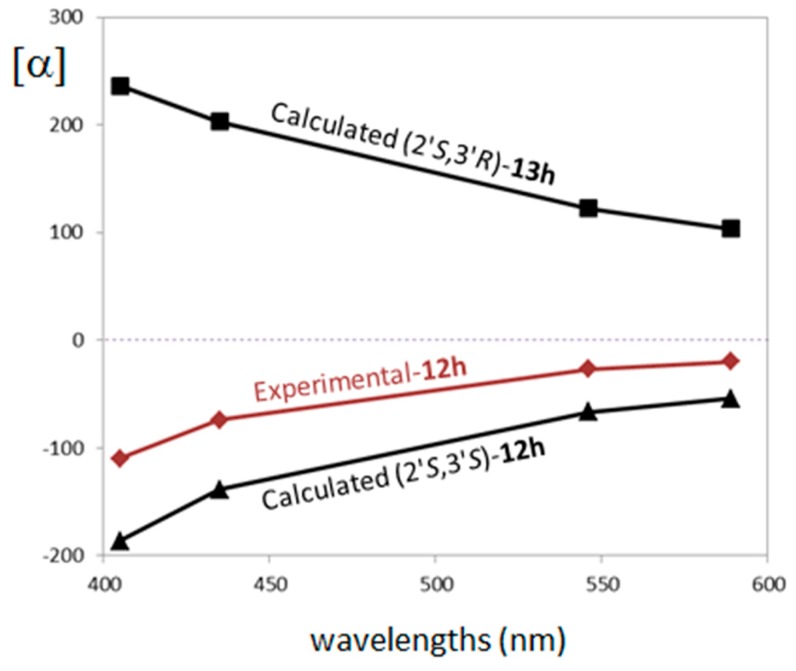
Comparison of experimental and computed optical rotatory dispersion (ORD) spectra for the different ACs for compounds *trans*-**12h** (experimental and calculated) and *cis*-**13h** (calculated).

Interestingly, the calculations of ORD spectra are sensitive to the 3′ carbon configuration. The experimental ORD curve was defined by measuring OR at four wavelengths 589, 546, 435 and 405 nm in chloroform solvent. The experimental ORD trend is negative in sign with specific rotation values from −20 to −110. The calculation was performed at CAM-B3LYP/6-311++G(d,p)/PCM(CHCl_3_) level at the measured wavelengths. In Figure 3 we report a comparison between experimental and calculated ORD curves.

**Figure 4:**
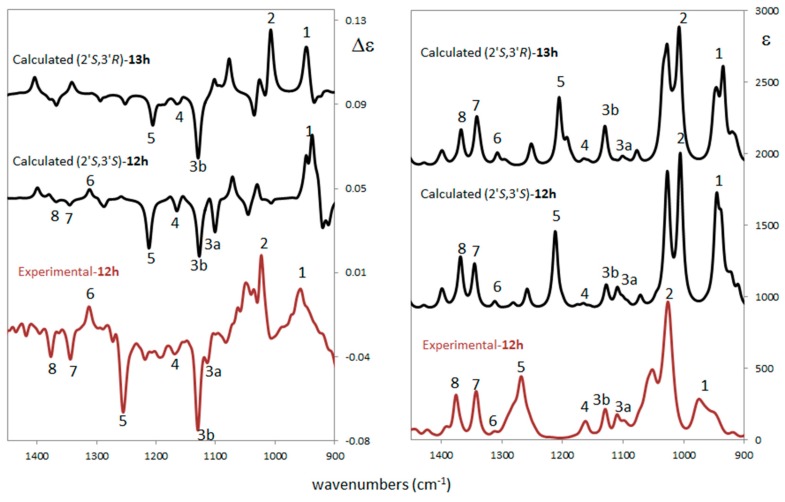
Comparison of experimental and computed vibrational circular dichroism (VCD) (**left**) and infrared (IR) (**right**) spectra for the different ACs for compound *trans*-**12h** (experimental and calculated) and *cis*-**13h** (calculated). Scaling factor = 0.98 (see Supplementary Materials for details).

The experimental ORD curve compares better to the one calculated for (2′*S*,3′*S*) with a specific rotation values from −54 to −186, while the (2′*S*,3′*R*) calculation provides a positive ORD trend and calculated rotation values from +103 to +236. It is worth noting that, like in the ECD case, the trend in sign of each separated conformer is consistent within each diastereomer calculation: the (2′*S*,3′*S*) conformer’s distribution presents negative specific rotation values, while the (2′*S*,3′*R*) diastereomer presents positive values for all conformers (see supporting material Figure SM-3 for details). These results strongly suggest (2′*S*,3′*S*) is the AC of the experimentally analyzed compound *trans*-**12h**. Figure 4 shows the comparison of experimental and calculated VCD/infrared (IR) spectra (900–1500 cm^−1^ range) for the two possible configurations considered for compounds *trans*-**12h** and *cis*-**13h**.

Experimental VCD/IR spectra were measured in CCl_4_ solution while calculations were performed at the B3LYP/TZVP/PCM(CCl_4_) level. The VCD/IR case is similar to the ECD/UV: also, from this technique there is no doubt that configuration at 2′ is *S*, while it is difficult to establish the configuration at 3′. In fact, there is not a clear discrimination between the two calculated VCD spectra. Indeed, some of the experimental features suggest a co-presence of the two diastereomers. In detail, it is possible to note the experimental peaks 7 and 8 which are well predicted in both IR spectra and they are present (even if very weak) in the (2′*S*,3′*S*) VCD spectrum. Narrow feature 2 seems to arise from the (2′*S*,3′*R*) diastereomer, while experimental band 3a is predicted only in (2′*S*,3′*S*). An additional analysis is possible on the basis of the similarity indices S.I. and Sim_NN of experimental and calculated VCD spectra. The first index [39] varies between −1 (reversed AC assignment has been made) and +1 (right assignment has been made) without intensity sensitivity; the second index [32] is also sensitive to intensity. Empirically, it is possible to confirm that a good AC assignment is reached when S.I. ≥ 0.5. The largest S.I. and Sim_NN values are found for (2′*S*,3′*S*) with a S.I. = +0.44 and Sim_NN = +0.16; similarity indices for (2′*S*,3′*R*) are positive but quite smaller (S.I. = +0.34 and Sim_NN = +0.09). The (2′*S*,3′*S*) AC is slightly favored by vibrational analysis and this agrees with ORD results for the AC assignment of compound *trans*-**12h**.

In conclusion, the ECD/UV experimental and computational analysis confirms that the AC of carbon 2′ is no doubt *S* but is unable to discriminate the AC of the second chiral center on carbon 3′ since the relevant electronic transitions are farther from 3′. ORD analysis suggests (2′*S*,3′*S*) to be the AC with a well predicted ORD curve in sign and order of magnitude with respect to the experimental one. VCD/IR analysis confirms the ECD/UV conclusion for center 2′, but it does not give a clear answer for center 3′. A co-presence of both (2′*S*,3′*S*) and (2′*S*,3′*R*) diastereomers of *trans*-**12h** and *cis*-**13h** cannot be excluded, with a prevalence of (2′*S*,3′*S*) as suggested by similarity indices.

## 3. Materials and Methods

Solvents and common reagents were purchased from a commercial source and used without further purification. All the known α-ylideneoxindoles (**11a**–**b**, **11e**–**f**, **11h**–**k**, and **11m**–**v**) were synthesised according the literature [26,28,29,30], whereas the unknown substrates (**11c**, **11d**, **11g**, **11l**) were analogously prepared and fully characterised as reported in the Supplementary Materials. All reactions were monitored by thin-layer chromatography (TLC) carried out on Merck F-254 silica glass plates and visualized with UV light or by 5% phosphomolibdic acid/ethanol test. Flash chromatography was performed on Sigma-Aldrich silica gel (60, particle size: 0.040–0.063 mm). ^1^H-NMR and ^13^C-NMR were recorded in CDCl_3_ (99.8% in deuterium) using a Varian Gemini 300 spectrometer (300 MHz, Varian inc., Palo Alto, CA, USA). All chemical shifts are expressed in parts per million (δ scale) and are referenced to the residual protons of the NMR solvent (CDCl_3_, δ 7.24 ppm). Optical rotations were made with the enantioenriched samples on a Jasco DIP-370 digital polimeter using a Na-lamp. The diastereomeric ratio of the epoxides was determined by ^1^H-NMR analysis of the crude reaction mixtures. The enantioselectivities were determined by HPLC analysis on chiral stationary phase [TSP Spectra Series P200, UV detector at λ = 254 nm, using a Daicel Chiralpack IC column and Daicel Chiralpack IA column]. Infrared spectra (FT-IR) were obtained using a Bruker Vector 22 spectrometer (Bruker, Billerica, MA, USA); data are presented as the frequency of absorption (cm^−1^). Melting points were determined with a Mel-Temp. High-resolution mass spectrometry (HRMS) spectra were recorded with Micromass Q-TOF micro mass spectrometer (Waters Corporations, Milford, MA, USA) and Micromass LCT (ESI, Waters Corporations, Milford, MA, USA) with Lock-Spray-Injector (Injection Loop-Modus in a HPLC system, Waters, Alliance 2695).

ORD spectra of *trans-***12h** were recorded with Jasco DIP370 digital polarimeter at four different wavelengths (589, 546, 435, 405 nm) at a concentration of 0.35 g/100 mL in chloroform solution. Experimental ECD/UV spectra were obtained by a JASCO 815SE apparatus from 400 nm to 180 nm under the following experimental conditions: integration time 1 s, scan speed 200 nm/min, bandpass 1 nm, 10 accumulations. Concentration used was 0.00354 M in acetonitrile solution in a 0.1 mm pathlength quartz cuvette. IR and VCD spectra were collected on a JASCO FVS6000 FTIR (JASCO Corporation, Easton, ML, USA) equipped with a liquid N_2_-cooled MCT detector, 5000 accumulations were averaged in the 850–1500 cm^−1^ region at 4 cm^−1^ resolution. The spectra were obtained in CCl_4_ solutions, in 200 mm pathlength BaF_2_ cells for a concentration of 0.046 M.

### Synthesis and Characterization of Epoxides Trans-***12a**–**v***

The synthetic procedures and analytical characterization of the diastereoisomers *cis*-**13a**–**v** are listed in the Supplementary Materials.

#### Typical Experimental Procedure for the Synthesis of Epoxides

To a solution of the catalyst **1** (38 mg, 0.15 mmol) and *trans*-α-ylideneoxindoles **11** (0.5 mmol) in *n*Hexane for HPLC grade (2.7 mL) was added TBHP (5.5 M in decane solution, 0.6 mmol, 0.11 mL). The resultant heterogeneous mixture was maintained under stirring at room temperature (25 °C) until reaction completion (TLC *n*Hexane/EtOAc). Afterwards, the crude reaction mixture was purified by flash chromatography on silica gel (*n*Hexane/EtOAc) to furnish the expected epoxy oxindoles *trans*-**12** and *cis*-**13**.

*(2′R,3′R)-ethyl 1-methyl-2-oxospiro[indoline-3,2′-oxirane]-3′-carboxylate* **12a**. Following the above general procedure, *trans* diastereoisomer **12a** was obtained as a whitish solid in 61% yield after purification by flash chromatography on silica gel (nHexane/EtOAc = 7/3), m.p. 132–134 °C. IR (CHCl_3_): $\tilde{\nu }$ = 3033, 3010, 2984, 1736, 1709, 1618, 1495, 1473, 1376, 1347 cm^−1^. ^1^H-NMR (CDCl_3_, 300 MHz, 25 °C): δ (ppm) 1.26 (t, *J* = 7.2 Hz, 3H, CH_3_CH_2_O), 3.25 (s, 3H, CH_3_N), 4.18 (s, 1H, OCH), 4.24 (dq, *J* = 10.9 Hz, 7.2 Hz, 1H, CH_3_CHHO), 4.29 (dq, *J* = 10.9 Hz, 7.2 Hz, 1H, CH_3_CHHO), 6.89 (ddd, *J* = 7.9 Hz, 0.9 Hz, 0.6 Hz, 1H, CHarom), 7.04 (dt, *J* = 7.7 Hz, 0.9 Hz,1H, CHarom), 7.39 (dt, *J* = 7.9 Hz, 1.3 Hz, 1H, CHarom), 7.45 (ddd, *J* = 7.7 Hz, 1.3 Hz, 0.6 Hz, 1H, CHarom). ^13^C-NMR (CDCl3, 75 MHz, 25 °C): δ (ppm) 14.3, 27.0, 60.0, 60.3, 62.4, 109.1, 119.5, 123.3, 125.0, 131.3, 145.9, 165.9, 170.1. HRMS: exact mass calculated for (C13H13NNaO4) requires *m*/*z* 270.0742, found *m*/*z* 270.0741. Chiral-phase HPLC analysis: [Daicel Chiralpack IC 5μ, λ = 254 nm, nHexane/EtOH = 7/3, flow rate 1.0 mL/min]: Tmajor = 11.91 min, Tminor = 14.97 min *er* = 91:9. [α]D = −93 (c = 1.6 g/cm^3^ inCH2Cl2).

*(2′R,3′R)-ethyl 5-iodo-1-methyl-2-oxospiro[indoline-3,2′-oxirane]-3′-carboxylate* **12b**. Following the above general procedure, *trans* diastereoisomer **12b** was obtained as a pale yellow solid in 32% yield after purification by flash chromatography on silica gel (nHexane/EtOAc = 7/3), m.p. 143–145 °C. IR (CHCl3): $\tilde{\nu }$ = 3028, 3017, 3008, 1736, 1727, 1611, 1535, 1486, 1358, 1340 cm^−1^. ^1^H-NMR (CDCl_3_, 300 MHz, 25 °C): δ (ppm) 1.33 (t, *J* = 7.1 Hz, 3H, CH_3_CH_2_O); 3.24 (s, 3H, NCH_3_); 3.94–4.41 (m, 3H, CH_3_CH_2_O, OCH); 6.69 (t, *J* = 8.2 Hz, 1H, CHarom); 6.68–6.78 (m, 2H, CHarom). ^13^C-NMR (CDCl_3_, 75 MHz, 25 °C): δ (ppm) 14.3, 27.0, 59.6, 60.0, 62.6, 85.6, 111.0, 121.7, 133.6, 140.0, 145.4, 165.5, 169.3. HRMS: exact mass calculated for (C_13_H_12_INNaO_4_) requires *m*/*z* 395.9709, found *m*/*z* 395.9712. Chiral-phase HPLC analysis: [Daicel Chiralpack IC 5μ, λ = 254 nm, nHexane/EtOH = 70/30, flow rate 1.0 mL/min]: T_major_ = 13.04 min, T_minor_ = 10.19 min *er* = 80:20. [α]_D_ = −9 (c = 0.0140 g/cm^3^ in CHCl_3_).

*(2′R,3′R)-ethyl 5,7-dichloro-1-methyl-2-oxospiro[indoline-3,2′-oxirane]-3′-carboxylate* **12c**. Following the above general procedure, *trans* diastereoisomer **12c** was obtained as a pale yellow solid in 38% yield after purification by flash chromatography on silica gel (nHexane/EtOAc = 8/2), m.p. 98–100 °C. IR (CHCl_3_): $\tilde{\nu }$ = 3025, 3011, 2959, 1739, 1727, 1625, 1494, 1471, 1369, 1346 cm^−1^. ^1^H-NMR (CDCl_3_, 300 MHz, 25 °C): δ (ppm) 1.20 (d, *J* = 6.8 Hz, 6H, (CH_3_)_2_CHC_arom_); 1.29 (t, *J* = 7.1 Hz, 3H, CH_3_CH_2_O); 2.80–2.95 (m, 1H, (CH_3_)_2_CHC_arom_); 3.26 (s, 3H, NCH_3_); 4.18–4.41 (m, 3H, CH_3_CH_2_O, OCH); 6.84 (d, *J* = 7.9 Hz, 1H, CH_arom_); 7.24 (d, *J* = 7.9 Hz, 1H, CH_arom_); 7.33 (m, 2H, CH_arom_). ^13^C-NMR (CDCl_3_, 75 MHz, 25 °C): δ (ppm) 14.3, 24.2 (2 × C), 26.9, 34.0, 59.9, 60.3, 62.3, 108.9, 119.3, 123.0, 129.0, 143.6, 144.3, 165.9, 170.0. HRMS: exact mass calculated for (C_16_H_19_NNaO_4_) requires *m*/*z* 312.1212, found *m*/*z* 312.1215. Chiral-phase HPLC analysis: [Daicel Chiralpack IC 5μ, λ = 254 nm, nHexane/EtOH = 70/30, flow rate 1.0 mL/min]: T_major_ = 14.85 min, T_minor_ = 8.42 min *er* = 89:11. [α]_D_ = −79 (c = 0.0220 g/cm^3^ in CHCl_3_).

*(2′R,3′R)-ethyl 5,7-dichloro-1-methyl-2-oxospiro[indoline-3,2′-oxirane]-3′- carboxylate* **12d**. Following the above general procedure, *trans* diastereoisomer **12d** was obtained as a pale red solid in 29% yield after purification by flash chromatography on silica gel (nHexane/EtOAc = 8/2), m.p. 124–126 °C. IR (CHCl_3_): $\tilde{\nu }$ = 3031, 3006, 1740, 1729, 1578, 1463, 1337, 1309 cm^−1^. ^1^H-NMR (CDCl_3_, 300 MHz, 25 °C): δ (ppm) 1.31 (t, *J* = 7.1 Hz, 3H, CH_3_CH_2_O); 3.62 (s, 3H, NCH_3_); 4.18–4.40 (m, 3H, CH_3_CH_2_O, OCH); 7.34 (s, 1H, CH_arom_); 7.40 (s, 1H, CH_arom_). ^13^C-NMR (CDCl_3_, 75 MHz, 25 °C): δ (ppm) 14.3, 30.5, 59.4, 60.6, 62.8, 117.0, 123.6, 124.0, 129.0, 132.9, 140.2, 165.1, 170.1. HRMS: exact mass calculated for (C_13_H_11_Cl_2_NNaO_4_) requires *m*/*z* 337.9963, found *m*/*z* 337.9961. Chiral-phase HPLC analysis: [Daicel Chiralpack IC 5μ, λ = 254 nm, nHexane/EtOH=70/30, flow rate 1.0 mL/min]: T_major_ = 11.74 min, T_minor_ = 9.48 min *er* = 93:7. [α]_D_ = −96 (c = 0.0160 g/cm^3^ in CHCl_3_).

*(2′R,3′R)-ethyl 5-methoxy-1-methyl-2-oxospiro[indoline-3,2′-oxirane]-3′-carboxylate* **12e**. Following the above general procedure, *trans* diastereoisomer **12e** was obtained as a pale yellow solid in 50% yield after purification by flash chromatography on silica gel (nHexane /EtOAc = 8/2), m.p. 105–107 °C. IR (CHCl_3_): $\tilde{\nu }$ = 1735, 1734, 1614, 1492, 1467, 1358, 1260 cm^−1^. ^1^H-NMR (CDCl_3_, 300 MHz, 25 °C): δ (ppm) 1.28 (t, *J* = 7.1 Hz, 3H, CH_3_CH_2_O); 3.23 (s, 3H, NCH_3_); 3.75 (s, 3H, OCH_3_); 4.18 (s, 1H, OCH); 4.22–4.25 (m, 2H, CH_3_CH_2_O); 6.80 (d, *J* = 8.5 Hz, 1H, CH_arom_); 6.92 (d, *J* = 8.5 Hz, 1H, CH_arom_); 7.09 (s, 1H, CH_arom_). ^13^C-NMR (CDCl_3_, 75 MHz, 25 °C): δ (ppm) 14.3, 26.9, 56.0, 59.9, 62.3, 66.1, 109.5, 112.3, 115.9, 120.5, 139.1, 156.3, 165.8, 169.8 ppm. HRMS: exact mass calculated for (C_14_H_15_NNaO_4_) requires *m*/*z* 300.0848, found *m*/*z* 300.0844. HPLC analysis: [Daicel Chiralpack IC 5μ, λ = 254 nm, nHeptane/EtOH = 70/30, flow rate 1.0 mL/min]: T_major_ = 16.33 min, T_minor_ = 11.45 min *er* = 90:10. [α]_D_ = −10 (c = 0.0451 g/cm^3^ in CHCl_3_).

*(2′R,3′R)-ethyl 1-methyl-5-nitro-2-oxospiro[indoline-3,2′-oxirane]-3′- carboxylate* **12f**. Following the above general procedure, *trans* diastereoisomer **12f** was obtained as a pale yellow solid in 19% yield after purification by flash chromatography on silica gel (DCM 100%), m.p. 170–172 °C. IR (CHCl_3_): $\tilde{\nu }$ = 3031, 3017, 3009, 1754, 1743, 1617, 1533, 1494, 1340 cm^−1^. ^1^H-NMR (CDCl_3_, 300 MHz, 25 °C): δ (ppm) 1.34 (t, *J* = 7.1 Hz, 3H, CH_3_CH_2_O); 3.35 (s, 3H, NCH_3_); 4.23–4.42 (m, 3H, CH_3_CH_2_O, OCH); 7.03 (d, *J* = 8.9 Hz, 1H, CH_arom_); 8.37 (d, *J* = 7.4 Hz, 2H, CH_arom_). ^13^C-NMR (CDCl_3_, 75 MHz, 25 °C): δ (ppm) 14.2, 27.4, 59.4, 60.1, 62.9, 108.9, 120.4, 121.1, 128.0, 143.8, 150.9, 165.1, 170.1. HRMS: exact mass calculated for (C_13_H_12_N_2_NaO_6_) requires *m*/*z* 315.0593, found *m*/*z* 315.0596. Chiral-phase HPLC analysis: [Daicel Chiralpack IC 5μ, λ = 254 nm, nHexane/EtOH = 70/30, flow rate 1.0 mL/min]: T_major_ = 23.13 min, T_minor_ = 22.02 min *er* = 85:15. [α]_D_ = −29 (c = 0.0180 g/cm^3^ in CDCl_3_).

*(2′R,3′R)-ethyl 1-methyl-2-oxo-1,2-dihydrospiro[benzo[g]indole-3,2′-oxirane]-3′-carboxylate* **12g**. Following the above general procedure, *trans* diastereoisomer **12g** was obtained as a pale red solid in 50% yield after purification by flash chromatography on silica gel (*n*Hexane/EtOAc = 8/2), m.p. 185–187 °C. ^1^H-NMR (CDCl_3_, 300 MHz, 25 °C): δ (ppm) 1.26–1.30 (m, 3H, CH_3_CH_2_O); 3.88 (s, 3H, NCH_3_); 4.21–4.31 (m, 3H, OCH, CH_3_CH_2_O), 7.50–7.55 (m, 4H, CH_arom_); 7.86–7.88 (m, 1H, CH_arom_), 8.40–8.43 (m, 1H, CH_arom_). ^13^C-NMR (CDCl_3_, 75 MHz, 25 °C): δ (ppm) 14.3, 28.3, 59.2, 62.5, 63.6, 109.2, 118.5, 122.2, 123.7, 124.7, 126.6, 128.1, 129.2, 142.3, 149.3, 168.6, 170.1. HRMS: exact mass calculated for (C_17_H_15_NNaO_4_) requires *m*/*z* 320.0899, found *m*/*z* 320.0896. Chiral-phase HPLC analysis: [Daicel Chiralpack IC 5μ, λ = 254 nm, *n*Hexane/EtOH = 70/30, flow rate 1.0 mL/min]: T_major_ = 18.48 min, T_minor_ = 13.56 min *er* = 91:9. [α]_D_ = −20.15 (c = 0.0059 g/cm^3^ in CHCl_3_).

*(2′S,3′S)-diethyl 1-methyl-2-oxospiro[indoline-3,2′-oxiran]-3′-yl)phosphonate* **12h**. Following the above general procedure, *trans* diastereoisomer **12h** was obtained as a pale yellow solid in 45% yield after purification by flash chromatography on silica gel (nHexane/EtOAc = 4/6), m.p. 166–168 °C. IR (CHCl_3_): $\tilde{\nu }$ = 1725, 1236 cm^−1^. ^1^H-NMR (CDCl_3_, 300 MHz, 25 °C): δ (ppm) 1.21 [t, *J* = 7.0 Hz, 3H, (CH_3_CH_2_O)_2_P], 1.41 [t, *J* = 7.1 Hz, 3H, (CH_3_CH_2_O)_2_P], 3.26 (s, 3H, NCH_3_), 3.73 (d, *J_HP_* = 27.6 Hz, 1H, OCH), 3.95–4.14 [m, 2H, (CH_3_CH_2_O)_2_P], 4.20–4.37 [m, 2H, (CH_3_CH_2_O)_2_P], 6.90 (d, *J* = 7.9 Hz, 1H, CH_arom_), 7.1 (dt, *J* = 7.7, 1.0 Hz, 1H, CH_arom_), 7.39 (dt, *J* = 7.9, 1.3 Hz, 1H, CH_arom_), 7.99 (d, *J* = 7.7 Hz, 1H, CH_arom_). ^13^C-NMR (CDCl_3_, 75 MHz, 25 °C): δ (ppm) 15.9 (d, *J_CCOP_* = 5.8 Hz), 16.0 (d, *J_CCOP_* = 5.7 Hz), 26.3, 55.5 (d, *J_CP_* = 203.5 Hz), 59.7, 62.7 (d, *J_COP_* = 6.3 Hz), 63.1 (d, *J_COP_* = 6.1 Hz), 108.4, 118.9, 122.6, 126.5, 130.6, 145.3, 170.2. HRMS: exact mass calculated for (C_14_H_18_NNaO_5_P) requires *m*/*z* 334.0820, found *m*/*z* 334.0824. Chiral-phase HPLC analysis: [Daicel Chiralpack IB 5μ, λ = 254 nm, nHexane/iPrOH = 9/1, flow rate 1.0 mL/min]: T_major_ = 42.20 min, T_minor_ = 23.60 min *er* = 80:20. [α]_D_ = −2 (c = 0.0101 g/cm^3^ in CHCl_3_).

*(2′S,3′S)-diethyl 1-phenyl-2-oxospiro[indoline-3,2′-oxiran]-3′-yl phosphonate* **12i**. Following the above general procedure, *trans* diastereoisomer **12i** was obtained as a pale yellow solid in 57% yield after purification by flash chromatography on silica gel (nHexane/EtOAc = 1/1), m.p. 197–199 °C. IR (CHCl_3_): $\tilde{\nu }$ = 1716, 1265 cm^−1^. ^1^H-NMR (CDCl_3_, 300 MHz, 25 °C): δ (ppm) 1.18 [t, *J* = 7.1 Hz, 3H, (CH_3_CH_2_O)_2_P], 1.34 [t, *J* =7.1 Hz, 3H, (CH_3_CH_2_O)_2_P], 3.76 (d, *J_HP_* = 27.6 Hz, 1H, OCH), 4.00–4.08 [m, 2H, (CH_3_CH_2_O)_2_P], 4.18–4.33 [m, 2H, (CH_3_CH_2_O)_2_P], 6.77 (d, *J* = 7.6 Hz, 1H, CH_arom_), 7.05 (t, *J* = 7.6 Hz, 1H, CH_arom_), 7.22 (t, *J* = 7.6 Hz, 1H, CH_arom_), 7.35–7.46 (m, 5H, CH_arom_), 7.96 (d, *J* = 7.6 Hz, 1H, CH_arom_). ^13^C-NMR (CDCl_3_, 75 MHz, 25 °C): δ (ppm) 16.0 (d, *J_CCOP_* = 5.7 Hz), 16.2 (d, *J_CCOP_* = 5.8 Hz), 58.1 (d, *J_CP_* = 203.7 Hz), 60.1, 62.9 (d, *J_COP_* = 6.2 Hz), 63.4 (d, *J_COP_* = 6.1 Hz), 109.8, 118.8, 123.3, 126.1, 126.9, 128.3, 129.5, 130.6, 133.5, 145.5, 169.8 ppm. HRMS: exact mass calculated for (C_19_H_20_NNaO_5_P) requires *m*/*z* 396.0977, found *m*/*z* 396.0975. Chiral-phase HPLC analysis: [Daicel Chiralpack IB 5μ, λ = 254 nm, nHexane/iPrOH = 9/1, flow rate 1.0 mL/min]: T_major_ = 39.90 min, T_minor_ = 17.50 min *er* = 79:21. [α]_D_ = −6.7 (c = 0.0122 g/cm^3^ in CHCl_3_).

*(2′S,3′S)-diethyl 1-benzyl-2-oxospiro[indoline-3,2′-oxiran]-3′-yl phosphonate* **12j**. Following the above general procedure, *trans* diastereoisomer **12j** was obtained as a yellow solid in 45% yield after purification by flash chromatography on silica gel (nHexane/EtOAc = 1/1), m.p. 205–207 °C. IR (CHCl_3_): $\tilde{\nu }$ = 1731, 1262 cm^−1^. ^1^H-NMR (CDCl_3_, 300 MHz, 25 °C): δ (ppm) 1.22 [t, *J* = 7.1 Hz, 3H, (CH_3_CH_2_O)_2_P], 1.43 [t, *J* = 7.1 Hz, 3H, (CH_3_CH_2_O)_2_P], 3.81 (d, *J_HP_* = 27.4 Hz, 1H, OCH), 4.02–4.14 [m, 2H, (CH_3_CH_2_O)_2_P], 4.24–4.39 [m, 2H, (CH_3_CH_2_O)_2_P], 4.96 (s, 2H, CH_2_N), 6.81 (d, *J* = 7.6 Hz, 1H, CH_arom_), 7.07 (t, *J* = 7.6 Hz, 1H, CH_arom_), 7.24–7.36 (m, 6H, CH_arom_), 8.00 (d, *J* = 7.6 Hz, 1H, CH_arom_). ^13^C-NMR (CDCl_3_, 75 MHz, 25 °C): δ (ppm) 16.2 (d, *J_CCOP_* = 5.7 Hz), 16.4 (d, *J_CCOP_* = 5.8 Hz), 44.4, 58.1 (d, *J_CP_* = 203.1 Hz), 60.2 (d, *J_CCP_* = 1.1 Hz), 62.9 (d, *J_COP_* = 6.0 Hz), 63.3 (d, *J_COP_* = 6.0 Hz), 109.3, 119.2, 122.1, 127.3, 127.5, 127.7, 128.7, 131.7, 135.2, 145.5, 170.7 ppm. HRMS: exact mass calculated for (C_20_H_22_NNaO_5_P) requires *m*/*z* 410.1133, found *m*/*z* 410.1130. Chiral-phase HPLC analysis: [Daicel Chiralpack IB 5μ, λ = 254 nm, nHexane/iPrOH = 9/1, flow rate 1.0 mL/min]: T_major_ = 39.90 min, T_minor_ = 17.50 min *er* = 74:26. [α]_D_ = −6.7 (c = 0.0122 g/cm^3^ in CHCl_3_).

*(2′S,3′S)-diethyl 1-(2,4-dichlorobenzyl)-2-oxospiro[indoline-3,2′-oxiran]-3′-yl)phosphonate* **12k**. Following the above general procedure, *trans* diastereoisomer **12k** was obtained as a pale yellow solid in 54% yield after purification by flash chromatography on silica gel (nHexane/EtOAc = 1/1), m.p. 235–237 °C. IR (CHCl_3_): $\tilde{\nu }$ = 1733, 1253 cm^−1^. ^1^H-NMR (CDCl_3_, 300 MHz, 25 °C): δ (ppm) 1.23 [t, *J* = 7.1 Hz, 3H, (CH_3_CH_2_O)_2_P], 1.42 [t, *J* =7.1 Hz, 3H, (CH_3_CH_2_O)_2_P], 3.81 (d, *J_HP_* = 27.4 Hz, 1H, OCH), 3.97–4.19 [m, 2H, (CH_3_CH_2_O)_2_P], 4.22–4.39 [m, 2H, (CH_3_CH_2_O)_2_P], 5.03 (s, 2H, CH_2_N), 6.73 (d, *J* = 7.9 Hz, 1H, CH_arom_), 7.05–7.20 (m, 3H, CH_arom_), 7.30–7.34 (m, 3H, CH_arom_), 7.43 (d, *J* = 1.9 Hz, 1H, CH_arom_), 8.00 (d, *J* = 7.6 Hz, 1H, CH_arom_). ^13^C-NMR (CDCl_3_, 75 MHz, 25 °C): δ (ppm) 15.9 (d, *J_CCOP_* = 5.6 Hz), 16.0 (d, *J_CCOP_* = 5.5 Hz), 41.1, 57.9 (d, *J_CP_* = 203.0 Hz), 59.7 (d, *J_CCP_* = 1.1 Hz), 62.6 (d, *J_COP_* = 6.2 Hz), 63.1 (d, *J_COP_* = 6.2 Hz), 109.2, 118.9, 123.0, 126.8, 127.2, 128.8, 129.2, 130.6, 130.8, 133.2, 133.8, 144.0, 170.6 ppm. HRMS: exact mass calculated for (C_20_H_20_Cl_2_NNaO_5_P) requires *m*/*z* 478.0354, found *m*/*z* 478.0356. Chiral-phase HPLC analysis: [Daicel Chiralpack IB 5μ, λ = 254 nm, *n*Hexane/*i*PrOH = 9/1, flow rate 1.0 mL/min]: T_major_ = 26.20 min, T_minor_ = 16.80 min *er* = 72:28. [α]_D_ = −20.4 (c = 0.0091 g/cm^3^ in CHCl_3_).

*(2′S,3′S)-diethyl 5-chloro-1-methyl-2-oxospiro[indoline-3,2′-oxiran]-3′-yl)phosphonate* **12l**. Following the above general procedure, *trans* diastereoisomer **12l** was obtained as a white solid in 38% yield after purification by flash chromatography on silica gel (nHexane/EtOAc = 1/1), m.p. 211–215 °C. IR (CHCl_3_): $\tilde{\nu }$ = 1734, 1260 cm^−1^. ^1^H-NMR (CDCl_3_, 300 MHz, 25 °C): δ (ppm) 1.26 [t, *J* = 7.0 Hz, 3H, (CH_3_CH_2_O)_2_P], 1.42 [t, *J* =7.0 Hz, 3H, (CH_3_CH_2_O)_2_P], 3.26 (s, 3H, NCH_3_); 3.73 (d, *J_HP_* = 26.6 Hz, 1H, OCH), 4.05–4.18 [m, 2H, (CH_3_CH_2_O)_2_P], 4.25–4.34 [m, 2H, (CH_3_CH_2_O)_2_P], 6.83 (d, *J* = 8.3 Hz, 1H, CH_arom_), 7.38 (d, *J* = 8.3 Hz, 1H, CH_arom_), 8.02 (s, 2H, CH_arom_). ^13^C-NMR (CDCl_3_, 75 MHz, 25 °C): δ (ppm) 16.4 (d, *J_CP_* = 5.9 Hz), 16.6 (d, *J_CP_* = 5.6 Hz), 27.1, 58.1 (d, *J_CP_* = 203.4 Hz), 59.9, 63.3 (d, *J_CP_* = 6.3 Hz), 63.8 (d, *J_CP_* = 6.1 Hz), 109.8, 121.2, 127.5, 128.8, 131.0, 144.3, 170.4. HRMS: exact mass calculated for (C_14_H_17_ClNNaO_5_P) requires *m*/*z* 368.0431, found *m*/*z* 368.0433. Chiral-phase HPLC analysis: [Daicel Chiralpack IC 5μ, λ = 254 nm, nHexane/EtOH = 9/1, flow rate 1.0 mL/min]: T_major_ = 18.92 min, T_minor_ = 17.56 min *er* = 77:23. [α]_D_ = +4 (c = 0.0170 g/cm^3^ in CHCl_3_).

*(2′S,3′S)-2-oxospiro[indoline-3,2′-oxirane]-3′-carboxylate* **12m**. Following the above general procedure, *trans* diastereoisomer **12m** was obtained as a white solid in 62% yield after purification by flash chromatography on silica gel (nHexane/EtOAc = 7/3). IR (CHCl_3_): $\tilde{\nu }$ = 3433, 3035, 3009, 1751, 1726, 1622, 1474, 1340, 1318 cm^−1^. ^1^H-NMR (CDCl_3_, 300 MHz, 25 °C): δ (ppm) 1.29 (t, *J* = 7.1 Hz, 3H, CH*_3_*CH_2_O); 4.17–4.47 (m, 3H, CH_3_CH_2_O, OCH); 6.87–7.17 (m, 2H, CH_arom_); 7.27–7.51 (m, 2H, CH_arom_); 9.38 (s, 1H, NH). ^13^C-NMR (CDCl_3_, 75 MHz, 25 °C): δ (ppm) 14.3, 59.9, 60.5, 62.5, 111.4, 119.6, 123.3, 125.2, 131.3, 143.0, 165.7, 172.6. HRMS: exact mass calculated for (C_12_H_11_NNaO_4_) requires *m*/*z* 256.0586, found *m*/*z* 256.0582. Chiral-phase HPLC analysis: [Daicel Chiralpack IC 5μ, λ = 254 nm, nHexane/EtOH = 90/10, flow rate 1.0 mL/min]: T_major_ = 6.28 min, T_minor_ = 5.51 min *er* = 75:25. [α]_D_ = −84.22 (c = 0.0155 g/cm^3^ in CHCl_3_).

*(2′S,3′S)-ethyl 5-fluoro-2-oxospiro[indoline-3,2′-oxirane]-3′-carboxylate* **12n**. Following the above general procedure, *trans* diastereoisomer **12n** was obtained as a white solid in 32% yield after purification by flash chromatography on silica gel (nHexane/EtOAc = 7/3). IR (CHCl_3_): $\tilde{\nu }$ = 3429, 3207, 3031, 2979, 1715, 1767, 1752, 1630, 1481, 1319, 1231, 1204 cm^−1^. ^1^H-NMR (CDCl_3_, 300 MHz, 25 °C): δ (ppm) 1.32 (t, 3H, *J* = 7.1 Hz, CH_3_CH_2_O); 4.19 (s, 1H, OCH); 4.26–4.36 (m, 2H, CH_3_CH_2_O); 6.88 (m, 1H, CH_arom_); 7.06 (t, 1H, *J* = 8.4 Hz, CH_arom_); 7.24 (m, 1H, CH_arom_); 8.66 (s, 1H, NH). ^13^C-NMR (CDCl_3_, 75 MHz, 25 °C): δ (ppm) 14.3, 60.0, 60.5, 62.7, 111.9 (d, *J_CF_* = 7.9 Hz), 113.5 (d, *J_CF_* = 26.7 Hz), 117.9 (d, *J_CF_* = 23.9 Hz), 121.3 (d, *J_CF_* = 9.1 Hz), 138.8, 159.3 (d, *J_CF_* = 242.2 Hz), 165.4, 172.3. HRMS: exact mass calculated for (C_12_H_10_FNNaO_4_) requires *m*/*z* 274.0492, found *m*/*z* 274.0497. Chiral-phase HPLC analysis: [Daicel Chiralpack IC 5μ, λ = 254 nm, nHexane/EtOH = 90/10, flow rate 1.0 mL/min]: T_major_ = 10.44 min, T_minor_ = 9.05 min *er* = 93:7. [α]_D_ = −132 (c = 0.0155 g/cm^3^ in CHCl_3_).

*(2′S,3′S)-ethyl 5-chloro-2-oxospiro[indoline-3,2′-oxirane]-3′-carboxylate* **12o**. Following the above general procedure, *trans* diastereoisomer **12o** was obtained as a white solid in 33% yield after purification by flash chromatography on silica gel (nHexane/EtOAc = 1/1). IR (CHCl_3_): $\tilde{\nu }$ = 3433, 3213, 3021, 1764, 1755, 1602, 1441, 1240, 1228, 1213 cm^−1^. ^1^H-NMR (CDCl_3_, 300 MHz, 25 °C): δ (ppm) 1.32 (t, 3H, *J* = 7.1 Hz, CH_3_CH_2_O); 4.19 (s, 1H, OCH); 4.26–4.36 (m, 2H, CH_3_CH_2_O); 6.88 (m, 1H, CH_arom_); 7.06 (t, 1H, *J* = 8.4 Hz, CH_arom_); 7.24 (m, 1H, CH_arom_); 8.66 (s, 1H, NH). ^13^C-NMR (CDCl_3_, 75 MHz, 25 °C): δ (ppm) 14.3, 60.0, 60.5, 62.7, 112.1, 123.3, 125.9, 129.0, 131.3, 140.3, 165.4, 171.8. HRMS: exact mass calculated for (C_12_H_10_ClNNaO_4_) requires *m*/*z* 290.0196, found *m*/*z* 290.0198. Chiral-phase HPLC analysis: [Daicel Chiralpack IC 5μ, λ = 254 nm, nHexane/EtOH = 70/30, flow rate 1.0mL/min]: T_major_ = 5.15 min, T_minor_ = 4.66 min *er* = 73:27. [α]_D_ = −43 (c = 0.0108 g/cm^3^ in CHCl_3_).

*(2′S,3′S)-ethyl 5-iodo-2-oxospiro[indoline-3,2′-oxirane]-3′-carboxylate* **12p**. Following the above general procedure, *trans* diastereoisomer **12p** was obtained as a white solid in 71% yield after purification by flash chromatography on silica gel (nHexane/EtOAc = 1/1). IR (CHCl_3_): $\tilde{\nu }$ = 1765, 1760, 1602, 1441, 1240, 1228 cm^−1^. ^1^H-NMR (CDCl_3_, 300 MHz, 25 °C): δ (ppm) 1.34 (t, 3H, *J* = 7.0 Hz, CH_3_CH_2_O); 4.17–4.40 (m, 3H, CHO, CH_3_CH_2_O); 6.76 (d, 1H, *J* = 8.3 Hz, CH_arom_); 7.67 (d, 1H, *J* = 8.3 Hz, CH_arom_); 7.76 (s, 1H, CH_arom_), 8.73 (bs, 1H, NH). ^13^C-NMR (CDCl_3_, 75 MHz, 25 °C): δ (ppm) 14.4, 59.8, 60.1, 62.7, 85.6, 113.0, 122.0, 134.2, 140.1, 142.4, 165.4, 171.2. HRMS: exact mass calculated for (C_12_H_10_INNaO_4_) requires *m*/*z* 381.9552, found *m*/*z* 381.9555. Chiral-phase HPLC analysis: [Daicel Chiralpack IC 5μ, λ = 254 nm, nHexane/EtOH = 90/10, flow rate 1.0 mL/min]: T_major_ = 11.56 min, T_minor_ = 9.81 min *ee* = 68:32. [α]_D_ = −23 (c = 0.0120 g/cm^3^ in CHCl_3_).

*(2′S,3′S)-ethyl 2-oxo-5-(trifluoromethoxy)spiro[indoline-3,2′-oxirane]-3′-carboxylate* **12q**. Following the above general procedure, *trans* diastereoisomer **12q** was obtained as a white solid in 29% yield after purification by flash chromatography on silica gel (nHexane/EtOAc = 7/3). IR (CHCl_3_): $\tilde{\nu }$ = 3433, 3210, 3028, 1764, 1724, 1630, 1478, 1371, 1234, 1197 cm^−1^. ^1^H-NMR (CDCl_3_, 300 MHz, 25 °C): δ (ppm) 1.31 (t, 3H, *J* = 7.0 Hz, CH_3_CH_2_O); 4.20 (s, 1H, OCH); 4.31 (q, 2H, *J* = 7.0 Hz, CH_3_CH_2_O); 6.97 (d, 1H, *J* = 8.4 Hz, CH_arom_); 7.22–7.26 (m, 1H, CH_arom_); 7.41 (s, 1H, CH_arom_); 8.41 (bs, 1H, NH). ^13^C-NMR (CDCl_3_, 75 MHz, 25 °C): δ (ppm) 14.2, 60.1, 60.0, 62.7, 111.6, 119.6, 120.6 (q, *J_CF_* = 256.0 Hz), 121.3, 124.6, 141.3, 145.1, 165.3, 171.7. HRMS: exact mass calculated for (C_13_H_10_F_3_NNaO_5_) requires *m*/*z* 340.0409, found *m*/*z* 340.0410. Chiral-phase HPLC analysis: [Daicel Chiralpack IC 5μ, λ = 254 nm, nHexane/EtOH = 95/5, flow rate 1.0mL/min]: T_major_ = 12.04 min, T_minor_ = 9.15 min *er* = 87:13. [α]_D_ = −56.53 (c = 0.0168 g/cm^3^ in CHCl_3_).

*(2′S,3′S)-ethyl 7-fluoro-2-oxospiro[indoline-3,2′-oxirane]-3′-carboxylate* **12r**. Following the above general procedure, *trans* diastereoisomer **12r** was obtained as a white solid in 21% yield after purification by flash chromatography on silica gel (nHexane/EtOAc = 8/2). IR (CHCl_3_): $\tilde{\nu }$ = 3433, 3031, 2976, 2930, 1752, 1737, 1639, 1493, 1234, 1228 cm^−1^. ^1^H-NMR (CDCl_3_, 300 MHz, 25 °C): δ (ppm) 1.30 (t, 3H, *J* = 7.0 Hz, CH_3_CH_2_O); 4.20 (s, 1H, OCH); 4.25–4.30 (m, 2H, CH_3_CH_2_O); 7.01–7.05 (m, 1H, CH_arom_); 7.14 (t, 1H, *J* = 9.2 Hz, CH_arom_); 7.28 (d, 1H, *J* = 9.2 Hz, CH_arom_); 7.84 (s, 1H, NH). ^13^C-NMR (CDCl_3_, 75 MHz, 25 °C): δ (ppm) 14.3, 60.0, 60.1, 62.5, 118.4 (d, *J_CF_* = 17 Hz), 121.1 (d, *J_CF_* = 3.6 Hz), 122.2, 124.0 (d, *J_CF_* = 5.9 Hz), 129.9, 147.4 (d, *J_CF_* = 244.7 Hz), 165.3, 170.5. HRMS: exact mass calculated for (C_12_H_10_FNNaO_4_) requires *m*/*z* 274.0492, found *m*/*z* 274.0493. Chiral-phase HPLC analysis: [Daicel Chiralpack IC 5μ, λ = 254 nm, nHexane/EtOH = 8/2, flow rate 1.0 mL/min]: T_major_ = 7.88 min, T_minor_ = 8.60 min *er* = 92:8. [α]_D_ = −63.2 (c = 0.0150 g/cm^3^ in CHCl_3_).

*(2′S,3′S)-ethyl 7-chloro-2-oxospiro[indoline-3,2′-oxirane]-3′-carboxylate* **12s**. Following the above general procedure, *trans* diastereoisomer **12s** was obtained as a white solid in 35% yield after purification by flash chromatography on silica gel (nHexane/EtOAc = 7/3). IR (CHCl_3_): $\tilde{\nu }$ = 3420, 3177, 3009, 1764, 1749, 1624, 1478, 1316, 1234, 1189 cm^−1^. ^1^H-NMR (CDCl_3_, 300 MHz, 25 °C): δ (ppm) 1.29 (t, 3H, *J* = 6.8 Hz, CH_3_CH_2_O); 4.20–4.31 (m, 2H, CH_3_CH_2_O, OCH); 7.01 (t, 1H, *J* = 8.1 Hz, CH_arom_); 7.36 (t, 2H, *J* = 8.1 Hz, CH_arom_); 8.14 (s, 1H, NH). ^13^C-NMR (CDCl_3_, 75 MHz, 25 °C): δ (ppm) 14.2, 60.1, 60.7, 62.5, 116.3, 121.3, 123.6, 124.1, 131.1, 140.4, 165.3, 171.2. HRMS: exact mass calculated for (C_12_H_10_ClNNaO_4_) requires *m*/*z* 290.0196, found *m*/*z* 290.0197. Chiral-phase HPLC analysis: [Daicel Chiralpack IC 5μ, λ = 254 nm, nHexane/EtOH = 8/2, flow rate 1.0 mL/min]: T_major_ = 7.72 min, T_minor_ = 10.00 min *er* = 88:12. [α]_D_ = −186.6 (c = 0.0220 g/cm^3^ in CHCl_3_).

*(2′S,3′S)-ethyl 7-bromo-2-oxospiro[indoline-3,2′-oxirane]-3′-carboxylate* **12t**. Following the above general procedure, *trans* diastereoisomer **12t** was obtained as a white solid in 49% yield after purification by flash chromatography on silica gel (nHexane/EtOAc = 7/3). IR (CHCl_3_): $\tilde{\nu }$ = 3414, 3210, 3006, 1742, 1730, 1621, 1444, 1316, 1234 cm^−1^. ^1^H-NMR (CDCl_3_, 300 MHz, 25 °C): δ (ppm) 1.29 (t, 3H, *J* = 7.1 Hz, CH_3_CH_2_O); 4.20 (s, 1H, OCH); 4.23–4.37 (m, 2H, CH_3_CH_2_O); 6.96 (t, 1H, *J* = 7.9 Hz, CH_arom_); 7.42 (d, 1H, *J* = 7.9 Hz, CH_arom_); 7.48 (d, 1H, *J* = 7.9 Hz, CH_arom_); 8.06 (s, 1H, NH). ^13^C-NMR (CDCl_3_, 75 MHz, 25 °C): δ (ppm) 14.3, 60.1, 61.0, 62.6, 104.1, 121.4, 124.3, 124.5, 133.9, 141.9, 165.3, 170.5. HRMS: exact mass calculated for (C_12_H_10_BrNNaO_4_) requires *m*/*z* 333.9691, found *m*/*z* 333.9693. Chiralphase HPLC analysis: [Daicel Chiralpack IC 5μ, λ = 254 nm, nHexane/EtOH = 7/3, flow rate 1.0mL/min]: T_major_ = 6.14 min, T_minor_ = 7.18 min *er* = 92:8. [α]_D_ = −15.85 (c = 0.0132 g/cm^3^ in CHCl_3_).

*(2′S,3′S)-ethyl 5,7-dichloro-2-oxospiro[indoline-3,2′-oxirane]-3′-carboxylate* **12u**. Following the above general procedure, *trans* diastereoisomer **12u** was obtained as a white solid in 27% yield after purification by flash chromatography on silica gel (nHexane/EtOAc = 7/3). IR (CHCl_3_): $\tilde{\nu }$ = 3423, 3031, 3009, 1757, 1743, 1463, 1323, 1290 cm^−1^. ^1^H-NMR (CDCl_3_, 300 MHz, 25 °C): δ (ppm) 1.32 (t, *J* = 7.0 Hz, 3H, CH_3_CH_2_O); 4.18–4.36 (m, 3H, CH_3_CH_2_O, OCH); 7.38 (s, 1H, CH_arom_); 7.42 (s, 1H, CH_arom_); 8.09 (s, 1H, NH). ^13^C-NMR (CDCl_3_, 75 MHz, 25 °C): δ (ppm) 14.3, 60.1, 60.4, 62.9, 116.6, 122.4, 124.4, 129.3, 130.8, 138.9, 165.1, 170.1. HRMS: exact mass calculated for (C_12_H_9_Cl_2_NNaO_4_) requires *m*/*z* 323.9806, found *m*/*z* 323.9808. Chiral-phase HPLC analysis: [Daicel Chiralpack IC 5μ, λ = 254 nm, nHexane/EtOH = 7/3, flow rate 1.0 mL/min]: T_major_ = 5.52 min, T_minor_ = 6.34 min *er* = 83:17. [α]_D_ = +137 (c = 0.0090 g/cm^3^ in CHCl_3_).

*(2R,3′R)-1-(tert-butyl) 3′-ethyl-2-oxospiro[indoline-3,2′-oxirane]-1,3′-dicarboxylate* **12v**. Following the above general procedure, *trans* diastereoisomer **12v** was obtained as a white solid in 60% yield after purification by flash chromatography on silica gel (nHexane/EtOAc = 7/3). IR (CHCl_3_): $\tilde{\nu }$ = 3035, 307, 2984, 1736, 1618, 1603, 1495, 1473, 1376, 1347 cm^−1^. ^1^H-NMR (CDCl_3_, 300 MHz, 25 °C): δ (ppm) 1.23 (t*, J* = 5.9 Hz, 3H, *CH_3_*CH_2_O), 1.48 (s, 9H), 4.22 (s, 2H, CH_3_*CH_2_*O), 4.59–4.45 (m, 1H), 7.22 (dtd, *J* = 26.3, 7.4, 2.0 Hz, 1H, CH_arom_), 7.38 (dd, *J* = 7.3, 2.2 Hz, 2H, CH_arom_), 7.89 (dd, *J* = 7.4, 2.1 Hz, 1H, CH_arom_). ^13^C-NMR (CDCl_3_, 75 MHz, 25 °C): δ (ppm) 14.0, 27.8, 60.5, 61.1, 69.2, 83.4, 114.4, 115.3, 125.2, 125.8, 129.3, 131.3, 150.5, 166.4, 170.8. HRMS: exact mass calculated for (C_17_H_19_NNaO_6_) requires *m*/*z* 356,1110, found *m*/*z* 356,1112. Chiralphase HPLC analysis: [Daicel Chiralpack IC 5μ, λ = 254 nm, nHexane/EtOH = 9/1, flow rate 1.0 mL/min]: T_major_ = 6.80 min, T_minor_ = 5.75 min *er* = 96:4. [α]_D_ = +27 (c = 0.0103 g/cm^3^ in CHCl_3_).

## 4. Conclusions

In summary, we have investigated the organocatalytic epoxidation of α-ylidene oxindoles in depth, highlighting the strong and weak features of the overall procedure. The above depicted findings clearly underline the importance of installing a robust H-bond network that mainly involves the catalyst and the substrate. Specifically, although a few organocatalysts having manifold scaffolds and diverse substituents have been tested, the simplest α,α-diphenylprolinol **1** results in being the most suitable structure to successfully perform the devised nucleophilic epoxidation in terms of yield, time and enantioselectivity.

Additionally, the extension of the substrate scope proves that the nucleophile attack on the electrophilic center (C_β_) only slightly endures the replacement of the EWG on the exocyclic double bond, while it is dramatically affected by the substitution pattern on the amidic moiety: (i) noticeable is the presence of the free NH function that makes the *Si*-face approach of the oxidizing agent less-hindered, and therefore preferential; (ii) the effect of the *tert*–butyloxycarbonyl protecting group able to induce excellent levels of stereoselectivity is also remarkable. Such unexpected findings complement our efforts to undertake supplementary mechanistic studies and establish additional improvements to the developed protocol, which not only should lead to a higher value of enantioselectivity, but also could furnish the desired spiroepoxyoxindoles bearing a quaternary stereogenic center with variable stereochemistry by simply modifying the nitrogen-protecting group.

## Figures and Tables

**Table 1:**
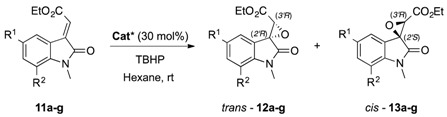
Effect of selected organocatalyst (**1**–**10**, Figure 1) on the asymmetric epoxidation of various *N*-methyl α-ylidene oxindoles (**11a**–**g**) bearing a carboxylate (CO_2_Et) as electron-withdrawing group (EWG) on the exocyclic double bond *^a,b^*.

Entry	Sub	R^1^	R^2^	Cat *	Time (h)	Yield (%) *^c^*	dr *^d^*	*trans*-12 *er ^e^*	*cis*-13 *er ^e^*
1	a	H	H	1	72	92	64:36	91:9	60:40
2	a	H	H	2	168	56	85:15	56:44	67:33
3	a	H	H	3	48	44	55:45	81:19	72:28
4	a	H	H	4	48	30	25:75	69:31	80:20
5	a	H	H	5	144	36	33:67	43:57	47:53
6	a	H	H	6	240	4	79:21	92:8	39:61
7	a	H	H	7	72	20	60:40	49:51	56:44
8	a	H	H	8	72	33	78:22	53:47	51:49
9	a	H	H	9	72	10	65:35	67:33	71:29
10	a	H	H	10	144	36	40:60	90:10	58:42
11	b *^f^*	I	H	1	24	97	33:67	80:20	66:34
12	c *^g^*	*^i^*Pr	H	1	96	90	42:58	89:11	64:36
13	d *^g^*	Cl	Cl	1	33	79	37:63	93:7	77:23
14	e *^h^*	OCH_3_	H	1	48	98	51:49	90:10	62:38
15	e *^h^*	NO_2_	H	1	48	72	27:73	85:15	62:38
16	g *^g^*	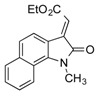		1	24	98	51:49	91:9	55:45

*^a^* Reaction conditions: *N*-Methyl α-ylideneoxindole **11** (0.5 mmol), catalyst **1** (0.15 mmol), THBP (0.6 mmol), and high-performance liquid chromatography (HPLC) grade hexane (2.7 mL) at rt. *^b^* The given stereochemical assignments are based on the previously reported data for spiroepoxides *trans*-**12a** and *cis*-**13a** [25]. *^c^* The yields of the isolated products are expressed as the sum of the diastereomers. *^d^* Determined by proton nuclear magnetic resonance (^1^H-NMR) of the crude reaction mixture. *^e^* Determined by chiral-phase HPLC analysis. *^f^* For the already known α-ylideneoxyndole **11b** see reference [28]. *^g^* The novel α-ylideneoxyndoles **11c**, **11d**, and **11g** were prepared and fully characterized as reported in the supporting material. *^h^* For the already known α-ylideneoxyndoles **11e** and **11f,** see reference [29]. *^i^* isopropyl.

**Table 2:**
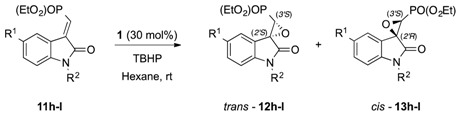
Substrate scope of the organocatalytic epoxidation on α-ylideneoxindoles **11h**–**l** bearing a PO(OEt)_2_ moiety as the EWG group of the exocyclic double bond *^a^*.

	Sub	R^1^	R^2^	Yield (%) *^b^*	dr *^c^*	*trans*-12 *er ^d^*	*cis*-13 *er ^d^*
1	**11h** *^e^*	H	CH_3_	80	56:44	80:20	61:39
2	**11i** *^e^*	H	Ph	95	60:40	79:21	62:38
3	**11j** *^e^*	H	Bn	80	56:44	74:26	55:45
4	**11k** *^e^*	H	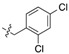	83	65:35	72:28	55:45
5	**11l** *^f^*	Cl	CH_3_	65	40:60	77:23	57:43

*^a^* Reaction conditions: α-ylideneoxindole **11**, (0.5 mmol) catalyst **1**, (0.15 mmol). THBP (0.6 mmol) and HPLC-grade hexane (2.7 mL) at rt. *^b^* The yields of the isolated products are expressed as the sum of the diastereomers. *^c^* Determined by ^1^H-NMR of the crude reaction mixture. *^d^* Determined by chiral-phase HPLC analysis. *^e^* For the already known α-ylideneoxyndole **11h**–**k** see reference [26]. *^f^* The novel α-ylideneoxyndole **11l** was prepared and fully characterized as reported in the Supporting Material.

**Table 3:**
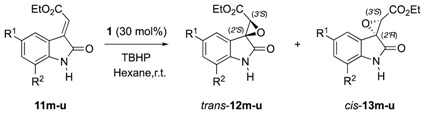
Substrate scope of the organocatalytic epoxidation of α-ylideneoxindoles **11m**–**u** featured with *N*-unprotected amide *^a^*.

	Sub	R^1^	R^2^	Time (h)	Yield (%) *^b^*	dr *^c^*	*trans*-12 *er ^d^*	*cis*-13 *er ^d^*
1	**11m***^e^*	H	H	144	96	65:35	75:25	61:39
2	**11n***^e^*	F	H	43	97	34:66	93:7	61:39
3	**11o***^e^*	Cl	H	140	83	40:60	73:27	59:41
4	**11p***^e^*	I	H	170	91	78:22	68:32	59:41
5	**11q***^e^*	OCF_3_	H	96	76	38:62	87:13	66:34
6	**11r***^e^*	H	F	66	93	23:77	92:8	70:30
7	**11s***^e^*	H	Cl	48	99	36:64	88:12	62:38
8	**11t***^e^*	H	Br	86	87	56:44	92:8	65:35
9	**11u***^e^*	Cl	Cl	78	42	35:65	83:17	86:14

*^a^* Reaction conditions: α-ylideneoxindole **11**. (0.5 mmol), catalyst **1** (0.15 mmol). THBP (0.6 mmol) and HPLC-grade hexane (2.7 mL) at rt. *^b^* The yields of the isolated products are expressed as the sum of the diastereomers. *^c^* Determined by ^1^H-NMR of the crude reaction mixture. *^d^* Determined by chiral-phase HPLC analysis. *^e^* For the already known α-ylideneoxyndole **11m**–**u,** see reference [30].

## Supplementary Materials

The following are available online.
