# Supplementary material for: Asymmetric Synthesis of Spirooxindoles via Nucleophilic Epoxidation Promoted by Bifunctional Organocatalysts
Source: Molecules. 2018 Feb 16;23(2):438. doi: 10.3390/molecules23020438 (PMC6017607; doi:10.3390/molecules23020438)
Supplement: Supplementary file 1 [file molecules-23-00438-s001.pdf]

Supplementary Material

for

Asymmetric synthesis of spirooxindoles via nucleophilic epoxidation promoted by bifunctional organocatalysts

Martina Miceli [1], Andrea Mazziotta [1], Chiara Palumbo [1], Elia Roma [1], Eleonora Tosi [1], Giovanna Longhi [2], Sergio Abbate [2], Paolo Lupattelli [3], Giuseppe Mazzeo[2], and Tecla Gasperi [1,*]

[1] Dipartimento di Scienze-Sezione di Nanoscienze e Nanotecnologie, Università degli Studi di Roma Tre, V.le G. Marconi 446, I-00146, Roma, Italia; martina.miceli@icloud.com; adma@kemi.dtu.dk; ch.palumbo@hotmail.it; elia.roma@uniroma3.it; eleonoratosi93@gmail.com; tecla.gasperi@uniroma3.it.

[2] Dipartimento di Medicina Molecolare e Traslazionale (DMMT), Università di Brescia, viale Europa 11, 25123 Brescia, Italia: giuseppe.mazzeo@unibs.it, giovanna.longhi@unibs.it; sergio.abbate@unibs.it.

[3] Dipartimento di Scienze, Università degli Studi della Basilicata, via dell'Ateneo Lucano 10, I-85100 Potenza, Italia; paolo.lupattelli@uniba.it

* Correspondence: tecla.gasperi@uniroma3.it; Tel.: +393386711045

Abstract: Taking into account the postulated reaction mechanism for the organocatalytic epoxidation of electron poor olefins developed by our laboratory, we have deeply investigated the key factors able to positively influence the H-bond network installed inside the substrate/catalyst/oxidizing agent. With this aim, we have (*i*) tested a few catalysts displaying various effects that noticeably differs in term of steric hindrance and electron demand, (*ii*) employed α-alkylidene oxindoles decorated with different substituents on the aromatic ring (**11a-g**) on the exocylic double bond (**11h-l**), and the amide moiety (**11m-v**). The observed results suggest that the modification of the EWG weakly conditions the overall outcomes, conversely a strong influence is unambiguously ascribable to the either *N*-protected or *N*-unprotected lactam framework. Specifically, when the NH free substrate (**11m-u**) are employed an inversion of the stereochemical control is observed, while the introduction of Boc protecting group afford the desired product **12v** in excellent enantioselectivity (97:3 *er*).

Keywords: Epoxidation; Organicatalysis; Epoxyoxindole; Alkylidenoxindoles, H-bond network. Non-covalent catalysis, Chiroptical properties.

1. General Information 2
2. Syntheses of α -ylideneoxindoles **11a-v** 2
   2.1 General Procedure for preparing α-ylideneoxindoles **11a-g** 2
   2.2. Characterization Data for α -ylideneoxindoles **11c**, **11d**, and **11g** 2
   2.3 General Procedure for preparing α -ylideneoxindoles **11h-l** 3
   2.4 Characterization Data for α -ylideneoxindoles **11l** 3
   2.6 General Procedure for preparing α -ylideneoxindoles **11n-u** 4
   2.8 Synthesis and characterization of α -ylideneoxindoles **11v** 4
3. Organocatalytic nucleophilic epoxidation of α-alkyliden oxindoles **11a-v** 5
   3.1 Experimental Procedure for the synthesis of Epoxides *trans* **12 a-v** and *cis* **13a-v** 5
   3.2 Characterization of epoxy oxindoles *trans* **12 a-v** and *cis* **13a-v** 5
4 Conformational analysis 16
   4.1 Calculation of ECD/UV spectra 17
   4.2 Calculation of ORD spectra 18
   4.3 Calculation of VCD/IR spectra 19

1. General Information

Solvents and common reagents were purchased from a commercial source and used without further purification. All the known α-ylideneoxindoles (**11a-b**, **11e-f**, **11h-k**, and **11m-v**) were synthesised according the literature [1-4], whereas the unknown substrates (**11c**, **11d**, **11g**, **11l**) were analogously prepared and fully characterised as reported in the Supporting Information. All reactions were monitored by thin layer chromatography (TLC) carried out on Merck F-254 silica glass plates and visualized with UV light or by 5% phosphomolibdic acid/ethanol test. Flash chromatography was performed on Sigma-Aldrich silica gel (60, particle size:0.040-0.063 mm). ^{1}H NMR and ^{13}C NMR were recorded in $CDCl_3$ (99.8% in deuterium) using a Varian Gemini 300 spectrometer (300 MHz). All chemical shifts are expressed in parts per million (δ scale) and are referenced to the residual protons of the NMR solvent ($CDCl_3$, δ 7.24 ppm). Optical rotations were made with the enantioenriched samples on a Jasco DIP-370 digital polimeter using a Na-lamp. The diastereomeric ratio of the epoxides was determined by ^{1}H NMR analysis of the crude reaction mixtures. The enantioselectivities were determined by HPLC analysis on chiral stationary phase [TSP Spectra Series P200, UV detector at λ = 254 nm, using Daicel Chiralpack IC column and Daicel Chiralpack IA column]. Infrared Spectra (FT-IR) were obtained using a Bruker Vector 22 spectrometer; data are presented as the frequency of absorption (cm^{-1}). Melting points were determined with a Mel-Temp. HRMS Spectra were recorded with Micromass Q-TOF micro Mass Spectrometer (Waters). Micromass LCT (ESI) with Lock-Spray-Injector (Injection Loop-Modus in a HPLC system, Waters, Alliance 2695). ORD spectra were recorded with Jasco DIP370 digital polarimeter at four different wavelengths (589, 546, 435, 405 nm) at concentration of 0.35 g/100 mL in chloroform solution. Experimental ECD/UV spectra were obtained by a JASCO 815SE apparatus from 400 to 180 nm under the following experimental conditions: integration time 1 s, scan speed 200 nm/min, bandpass 1 nm, 10 accumulations. Concentration used was 0.00354 M in acetonitrile solution in a 0.1 mm pathlength quartz cuvette. IR and VCD spectra were collected on a JASCO FVS6000 FTIR equipped with a liquid N_2-cooled MCT detector, 5000 accumulations were averaged in the 850-1500 cm^{-1} region at 4 cm^{-1} resolution. The spectra were obtained in CCl_4 solutions, in 200 mm pathlength BaF_2 cells for a concentration of 0.046 M.

2. Syntheses of 3-ylideneoxindoles 11a-v

*2.1 General Procedure for preparing α-ylideneoxindoles **11a-g***

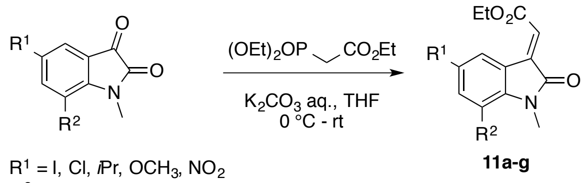

11a-g

R^1 = I, Cl, *i*Pr, OCH_3, NO_2
R^2 = H,Cl

To a stirred solution of *N*-methyl isatine (2.7 mmol) in THF (9.0 mL) at 0 °C triethyl phosphonoacetate (3.0 mmol) and a solution of K_2CO_3 (8.7 mmol) in water (1.8 mL) were added. The mixture was stirred at 0 °C for 15 min and once reached the room temperature was kept under stirring until the reaction completion (TLC Hexane /EtOAc). Afterwards, diethyl ether (50.0 mL) was added and the organic phase was washed with brine, dried with anhydrous Na_2SO_4 and concentrated under *vacuum*. The crude product was subsequently purified by flash chromatography on silica gel (*n*Hexane/EtOAc).

The analytical data of compounds **11a**, **11b**, **11e**, and **11f** were fully in agreement with the characterization reported in literature [1,3].

*2.2. Characterization Data for α-ylideneoxindoles **11c, 11d,** and **11g***

(E)-ethyl 2-(5-isopropyl-1-methyl-2-oxoindolin-3-ylidene) **11c**

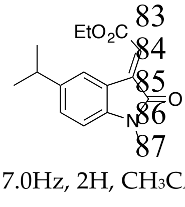

Following the general procedure, the single *E* diastereoisomer **11c** was obtained as an orange solid in 63% yield after purification by flash chromatography on silica gel (*n*Hexane/EtOAc=8/2), m.p. 70-72 °C. IR ($CHCl_3$): $\tilde{\nu}$= 3025, 3020, 3011, 1722, 1712, 1612, 1490, 1370 cm^{-1}. ^{1}H NMR ($CDCl_3$, 300MHz, 25 °C): δ (ppm) 1.26 (d, *J* = 7.0Hz, 6H, $(CH_3)_2$CH), 1.38 (t, *J* = 7.1Hz, 3H, CH_3CH_2O); 2.88–2.98 (m, 1H, $(CH_3)_2CHC$); 3.22 (s, 3H, NCH_3); 4.34 (q, *J* = 7.0Hz, 2H, CH_3CH_2O); 6.72 (d, *J* = 7.9Hz, 1H, CH_{arom}); 6.89 (s, 1H, CH=C); 7.24 (d, *J* = 7.9Hz, 1H, CH_{arom}); 8.47 (s,

1H, CH$_{arom}$). ^{13}C NMR (CDCl$_3$, 75MHz, 25 °C): δ (ppm) 14.3, 24.3, 26.4, 34, 61.2, 108, 119.9, 122.2, 127.2, 130.3, 138.3, 143.7, 144.1, 165.8, 167.7. HRMS: exact mass calculated for ($C_{16}H_{19}NNaO_3$) requires m/z 296.1263, found m/z 296.1265.

(E)-ethyl 2-(5,7-dichloro-1-methyl-2-oxoindolin-3-ylidene)acetate ***11d***

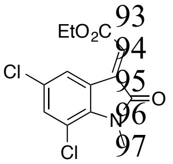

Following the general procedure, the single *E* diastereoisomer **11d** was obtained as an orange solid in 89% yield after purification by flash chromatography on silica gel (*n*Hexane/EtOAc=8/2), m.p. 148-150 °C. IR (CHCl$_3$): $\tilde{\nu}$= 3029, 3016, 1726, 1714, 1574, 1453, 1374, 1339 cm^{-1}. ^{1}H NMR (CDCl$_3$, 300MHz, 25 °C): δ (ppm) 1.36 (t, *J* = 6.9Hz, 3H, C*H*$_3$CH$_2$O); 3.54 (s, 3H, NCH$_3$); 4.31 (q, *J* = 6.9Hz, 2H, CH$_3$C*H*$_2$O); 6.90 (s, 1H, CH=C); 7.23 (s, 1H, CH$_{arom}$); 8.51 (s, 1H, CH$_{arom}$). ^{13}C NMR (CDCl$_3$, 75MHz, 25 °C): δ (ppm) 14.4, 30, 61.9, 116.1, 123.3, 125.4, 127.6, 128.4, 133.7, 135.7, 140.3, 165.2, 167.5. HRMS: exact mass calculated for ($C_{17}H_{15}NNaO_3$) requires m/z 322.0014, found m/z 322.0017.

(E)-ethyl 2-(1-methyl-2-oxo-1H-benzo[g]indol-3(2H)-ylidene)acetate ***11g***

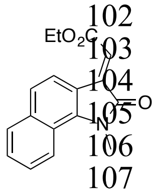

Following the general procedure, the single *E* diastereoisomer **11g** was obtained as an orange solid in 51% yield after purification by flash chromatography on silica gel (*n*Hexane/EtOAc=8/2), m.p. 180-182 °C. IR (CHCl$_3$): $\tilde{\nu}$ =3038, 3027, 1710, 1644, 1620, 1590, 1466, 1377 cm^{-1}. ^{1}H NMR (CDCl$_3$, 300MHz, 25 °C): δ (ppm) 1.39 (t, *J* = 6.8 Hz, 3H, C*H*$_3$CH$_2$O); 3.83 (s, 3H, NCH$_3$); 4.35 (q, *J* = 6.8 Hz, 2H, CH$_3$C*H*$_2$O); 6.97 (s, 1H, CH=C); 7.42-7.53 (m, 3H, CH$_{arom}$); 7.82 (d, *J* = 8.2 Hz, 1H, CH$_{arom}$); 8.41 (d, *J* = 8.2 Hz, 1H, CH$_{arom}$); 8.63 (d, *J* = 8.6 Hz, 1H, CH$_{arom}$). ^{13}C NMR (CDCl$_3$, 75MHz, 25 °C): δ (ppm) 14.3, 31.1, 61.4, 115.9, 120.9, 122.6, 122.8, 122.9, 124.2, 126.1, 127.6, 129.5, 137.0, 137.3, 143.5, 165.9, 169.4. HRMS: exact mass calculated for ($C_{17}H_{15}NNaO_3$) requires m/z 304.0950, found m/z 304.0971.

2.3 General Procedure for preparing α-ylideneoxindoles ***11h-l***

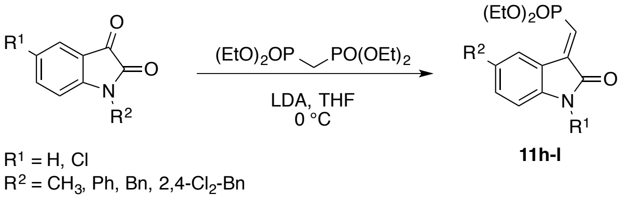

11h-l

To a stirred solution of tetraethyl methylenebis(phosphonate) (2.6 mmol) in anhydrous THF (2 mL) under Ar at 0 °C, LDA (2 m in THF, 1.43 mL) was added dropwise. After 30 min, the mixture was warmed to room temperature, stirred for an additional 30 min and cooled again to 0 °C. At this temperature, a solution of isatine (2 mmol) in anhydrous THF (8 mL) was slowly added. The reaction mixture could reach room temperature, and stirred until complete (monitored by TLC; hexane/ethyl acetate). Saturated aqueous NH_4Cl solution was slowly added, and the aqueous layer was extracted with ethyl acetate (3x50 mL). The combined organic phases were dried with anhydrous Na_2SO_4 and concentrated under reduced pressure to give the crude product, which was subsequently purified by flash chromatography on silica gel (hexane/ethyl acetate)

The analytical data of compounds **11h**-**k** were fully in agreement with the characterization reported in literature [3].

2.4 Characterization Data for α-ylideneoxindoles ***11l***

(E)-diethyl ((5-chloro-1-methyl-2-oxoindolin-3-ylidene)methyl)phosphonate ***11l***

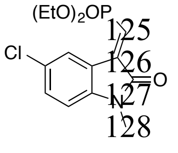

Following the general procedure, the single E diastereoisomer **11l** was obtained as an orange amorphous solid in 30% yield after purification (nHexane/EtOAc=6/4). IR (CHCl$_3$): $\tilde{\nu}$ = 1727, 1606 cm^{-1}. ^{1}H NMR (CDCl$_3$, 300MHz, 25 °C): δ (ppm) 1.34 [t, *J* = 7.0Hz, 6H, (CH$_3$CH$_2$O)$_2$P], 3.20 (s, 3H, CH$_3$N), 4.12–4.21 [m, 4H, (CH$_3$CH$_2$O)$_2$P], 6.71 (d, *J* = 8.2Hz, 1H, CH$_{arom}$), 6.85 (d, J_{HP} = 13.0Hz, 1H, CHP=O), 7.31 (d, *J* = 8.2Hz, 1H, CH$_{arom}$), 8.49 (s, 1H, CH$_{arom}$). ^{13}C NMR (CDCl$_3$, 75MHz, 25 °C): δ (ppm) 16.4, 26.5, 62.7 (d, J_{CP} = 5.6Hz), 109.2, 121.1 (d, J_{CP} = 7.0Hz), 121.4 (d, J_{CP} = 189.5Hz), 128.0, 128.5, 132.0,

140.5 (d, J_{CP} = 4.9Hz), 144.2, 166.2 (d, J_{CP} = 25.7Hz). HRMS: exact mass calculated for ($C_{14}H_{17}ClNNaO_4P$) requires m/z 352.0627, found m/z 352.0631.

2.6 General Procedure for preparing 3-ylideneoxindoles ***11n-u***

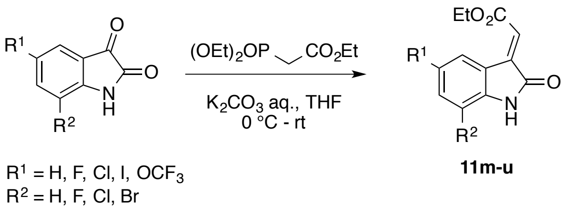

To a stirred solution of the simple isatine (2.7 mmol) in THF (9.0 mL) at 0 °C triethyl phosphonoacetate (3.0 mmol) and a solution of K_2CO_3 (8.7 mmol) in water (1.8 mL) were added. The mixture was stirred at 0 °C for 15 min and once reached the room temperature was kept under stirring until the reaction completion (TLC Hexane /EtOAc). Afterwards, diethyl ether (50.0 mL) was added and the organic phase was washed with brine, dried with anhydrous Na_2SO_4 and concentrated under *vacuum*. The crude product was subsequently purified by flash chromatography on silica gel (*n*Hexane/EtOAc).

The analytical data of compounds **11n-u** were fully in agreement with the characterization reported in literature [4].

2.8 Synthesis and characterization of α -ylideneoxindoles ***11v***

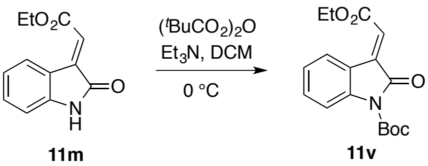

In a round bottomed flask, 262mg of Boc_2O were added drop by drop to a solution of α-ylideneoxindole **11m** (217 mg, 1 mmol) and DMAP (12 mg, 0.1 mmol) in CH_3CN (10mL). The solution was stirred at room temperature overnight. The solvent was removed under reduced pressure and the crude was purified *via* flash chromatography, yielding 314 mg (0.99 mmol) of clean product as a yellow solid.

The analytical data of compounds **11v** were fully in agreement with the characterization reported in literature [4].

3. Organocatalytic nucleophilic epoxidation of α-alkyliden oxindoles 11a-v

*3.1 Experimental Procedure for the synthesis of Epoxides trans **12 a-v** and cis **13a-v***

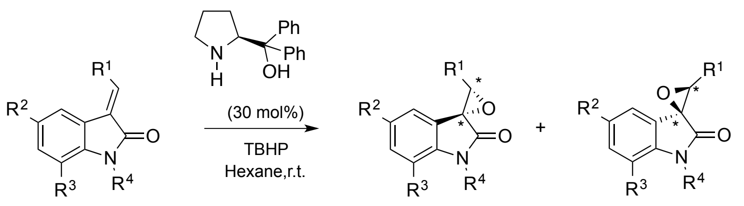

11a-v *trans* **12a-v** *cis* **13a-v**

To a solution of the catalyst **1** (38 mg, 0.15 mmol) and *trans*-α-ylideneoxindoles **11** (0.5 mmol) in *n*Hexane for HPLC grade (2.7 mL) was added TBHP (5.5 M in decane solution, 0.6 mmol, 0.11 mL). The resultant heterogeneous mixture was maintained under stirring at room temperature (25 °C) until the reaction completion (TLC *n*Hexane/EtOAc). Afterwards, the crude reaction mixture was purified by flash chromatography on silica gel (*n*Hexane/EtOAc) to furnish the expected epoxy oxindoles *trans*-**12** and *cis*-**13**.

*3.2 Characterization of epoxy oxindoles trans-**12** and cis-**13***

*(2'R,3'R)-ethyl 1-methyl-2-oxospiro[indoline-3,2'-oxirane]-3'-carboxylate **12a***

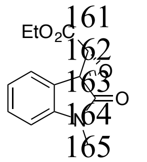

Following the above general procedure, *trans* diastereoisomer **12a** was obtained as a whitish solid in 61% yield after purification after purification by flash chromatography on silica gel (nHexane/EtOAc=7/3), m.p. 132-134 °C. IR ($CHCl_3$): $\tilde{\nu}$ = 3033, 3010, 2984, 1736, 1709,1618, 1495, 1473, 1376, 1347 cm^{-1}. ^{1}H NMR ($CDCl_3$, 300 MHz, 25 °C): δ (ppm) 1.26 (t, *J* = 7.2 Hz, 3H, CH_3CH_2O), 3.25 (s, 3H, CH_3N), 4.18 (s, 1H, OCH), 4.24 (dq, *J* = 10.9 Hz, 7.2 Hz, 1H, CH_3CHHO), 4.29 (dq, *J* = 10.9 Hz, 7.2 Hz, 1H,CH_3CHHO), 6.89 (ddd, *J* = 7.9 Hz, 0.9 Hz, 0.6 Hz, 1H, CH_{arom}), 7.04 (dt, *J* = 7.7 Hz, 0.9 Hz,1H, CH_{arom}), 7.39 (dt, *J* = 7.9 Hz, 1.3 Hz, 1H, CH_{arom}), 7.45 (ddd, *J* = 7.7 Hz, 1.3 Hz, 0.6 Hz, 1H, CH_{arom}). ^{13}C NMR ($CDCl_3$, 75 MHz, 25 °C): δ (ppm) 14.3, 27.0, 60.0, 60.3, 62.4, 109.1, 119.5, 123.3, 125.0, 131.3, 145.9, 165.9, 170.1. HRMS: exact mass calculated for ($C_{13}H_{13}NNaO_4$) requires *m*/*z* 270.0742, found *m*/*z* 270.0741. Chiral-phase HPLC analysis: [Daicel Chiralpack IC 5μ, λ=254 nm, nHexane/EtOH=7/3, flow rate 1.0mL/min]: T_{major} = 11.91 min, T_{minor} = 14.97 min *er* = 91:9. $[\alpha]_D$ = -93 (c = 1.6 g/cm^3 inCH_2Cl_2).

*(2'S,3'R)-ethyl 1-methyl-2-oxospiro[indoline-3,2'-oxirane]-3'-carboxylate, **13a***

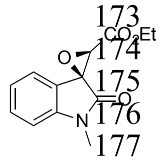

Following the above general procedure, *cis* diastereoisomer **13a** was obtained as a pale yellow solid in 34% yield after purification by flash chromatography on silica gel (nHexane/EtOAc=7/3), m.p. 160-161 °C IR ($CHCl_3$): $\tilde{\nu}$ = 3034, 3010, 2933, 1759, 1733, 1621, 1472, 1375, 1345 cm^{-1}. ^{1}H NMR ($CDCl_3$, 300 MHz, 25 °C): δ (ppm) 1.35 (t, *J* = 7.2 Hz, 3H, CH_3CH_2O), 3.22 (s, 3H, CH_3N), 4.15 (s, 1H, OCH), 4.34 (q, *J* = 7.2 Hz, 2H, CH_3CH_2O), 6.89 (dd, *J* = 7.8 Hz, 0.5 Hz, 1H, CH_{arom}), 7.04-7.14 (m, 2H, CH_{arom}), 7.40 (ddd, *J* = 7.9 Hz, 6.2 Hz, 2.8 Hz, 1H, CH_{arom}). ^{13}C NMR ($CDCl_3$, 75 MHz, 25 °C): δ (ppm) 14.3, 26.9, 60.3, 60.4, 62.3, 109.2, 121.4, 122.6, 123.2, 131.4, 145.5, 165.0, 169.0. HRMS: exact mass calculated for ($C_{13}H_{13}NNaO_4$) requires *m*/*z* 270.0742, found *m*/*z* 270.0739. Chiral-phase HPLC analysis: [Daicel Chiralpack IC 5μ, λ=254 nm, nHexane/EtOH=7/3, flow rate 1.0mL/min]: T_{major} = 14.60 min, T_{minor} = 19.82 min, *er* = 60:40. $[\alpha]_D$ = -105 (c = 0.035 g/cm^3 in $CHCl_3$).

*(2'R,3'R)-ethyl 5-iodo-1-methyl-2-oxospiro[indoline-3,2'-oxirane]-3'-carboxylate **12b***

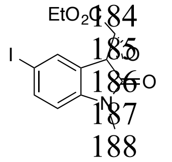

Following the above general procedure, *trans* diastereoisomer **12b** was obtained as a pale yellow solid in 32% yield after purification by flash chromatography on silica gel (nHexane/EtOAc=7/3), m.p. 143-145 °C. IR ($CHCl_3$): $\tilde{\nu}$= 3028, 3017, 3008, 1736, 1727, 1611, 1535, 1486, 1358, 1340 cm^{-1}. ^{1}H NMR ($CDCl_3$, 300 MHz, 25 °C): δ (ppm) 1.33 (t, *J* = 7.1 Hz, 3H, CH_3CH_2O); 3.24 (s, 3H, NCH_3); 3.94-4.41 (m, 3H, CH_3CH_2O, OCH); 6.69 (t, *J* = 8.2 Hz, 1H, CH_{arom}); 6.68-6.78 (m, 2H, CH_{arom}). ^{13}C NMR ($CDCl_3$, 75 MHz, 25°C): δ (ppm) 14.3, 27.0, 59.6, 60.0, 62.6, 85.6, 111.0, 121.7, 133.6, 140.0, 145.4, 165.5, 169.3. HRMS: exact mass calculated for ($C_{13}H_{12}INNaO_4$) requires *m*/*z* 395.9709, found *m*/*z* 395.9712. Chiral-phase HPLC analysis: [Daicel Chiralpack IC 5μ, λ=254 nm,

nHexane/EtOH=70/30, flow rate 1.0mL/min]: T_{major} = 13.04 min, T_{minor} = 10.19 min *er* = 80:20. [α] $_D$ = -9 (c = 0.0140 g/cm^3 in $CHCl_3$).

(2′S,3′R)-ethyl 5-iodo-1-methyl-2-oxospiro[indoline-3,2′-oxirane]-3′-carboxylate ***13b***

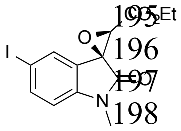

Following the above general procedure, *cis* diastereoisomer **13b** was obtained as a pale yellow solid in 32% yield after purification by flash chromatography on silica gel (nHexane/EtOAc=7/3), m.p. 165-170 °C. IR (CHCl3): $\tilde{\nu}$ = 3025, 3017, 3008, 1753, 1736, 1611, 1486, 1465, 1430, 1340 cm^{-1}. ^{1}H NMR ($CDCl_3$, 300 MHz, 25 °C): δ (ppm) 1.35 (t, *J* = 7.1 Hz, 3H, CH_3CH_2O); 3.21 (s, 3H, NCH_3); 4.14 (s, 1H, OCH_3); 4.35 (dq, *J* = 9.8 Hz, 7.1 Hz, 1H, CH_3CHHO); 4.38 (dq, *J* = 9.8 Hz, 7.1Hz, 1H, CH_3CHHO); 6.69 (d, *J* = 8.2 Hz, 1H, CH_{arom}); 7.37 (s, 1H, CH_{arom}); 7.71 (dd, *J* = 8.2 Hz, 1.5 Hz, 1H, CH_{arom}). ^{13}C NMR ($CDCl_3$, 75 MHz, 25 °C): δ (ppm) 14.3, 27.0, 59.6, 60.0, 62.6, 85.6, 111.0, 121.7, 133.6, 140.0, 145.4, 165.5, 169.3. HRMS: exact mass calculated for ($C_{13}H_{12}INNaO_4$) requires *m*/*z* 395.9709, found *m*/*z* 395.9712. Chiral-phase HPLC analysis: [Daicel Chiralpack IC 5μ, λ=254 nm, nHexane/EtOH=70/30, flow rate 1.0mL/min]: T_{major} = 14.94 min, T_{minor} = 19.58 min *er* = 66:34. [α]$_D$ = -9 (c = 0.0140 g/cm^3 in $CHCl_3$)

(2′R,3′R)-ethyl 5,7-dichloro-1-methyl-2-oxospiro[indoline-3,2′-oxirane]-3′- carboxylate ***12c***

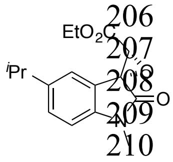

Following the above general procedure, *trans* diastereoisomer **12c** was obtained as a pale yellow solid in 38% yield after purification by flash chromatography on silica gel (nHexane/EtOAc=8/2), m.p. 98-100 °C. IR ($CHCl_3$): $\tilde{\nu}$= 3025, 3011, 2959, 1739, 1727, 1625, 1494, 1471, 1369, 1346 cm^{-1}. ^{1}H NMR ($CDCl_3$, 300 MHz, 25 °C): δ (ppm) 1.20 (d, *J* = 6.8 Hz, 6H, $(CH_3)_2CHC_{arom}$); 1.29 (t, *J* = 7.1 Hz, 3H, CH_3CH_2O); 2.80-2.95 (m, 1H, $(CH_3)_2CHC_{arom}$); 3.26 (s, 3H, NCH_3); 4.18-4.41 (m, 3H, CH_3CH_2O, OCH); 6.84 (d, *J* = 7.9 Hz, 1H, CH_{arom}); 7.24 (d, *J* = 7.9 Hz, 1H, CH_{arom}); 7.33 (m, 2H, CH_{arom}). ^{13}C NMR ($CDCl_3$, 75 MHz, 25 °C): δ (ppm) 14.3, 24.2 (2xC), 26.9, 34.0, 59.9, 60.3, 62.3, 108.9, 119.3, 123.0, 129.0, 143.6, 144.3, 165.9, 170.0. HRMS: exact mass calculated for ($C_{16}H_{19}NNaO_4$) requires *m*/*z* 312.1212, found *m*/*z* 312.1215. Chiral-phase HPLC analysis: [Daicel Chiralpack IC 5μ, λ=254 nm, nHexane/EtOH=70/30, flow rate 1.0mL/min]: T_{major} = 14.85 min, T_{minor} = 8.42 min *er* = 89:11. [α]$_D$ = -79 (c = 0.0220 g/cm^3 in $CHCl_3$).

(2′S,3′R)-ethyl 5,7-dichloro-1-methyl-2-oxospiro[indoline-3,2′-oxirane]-3′- carboxylate ***13c***

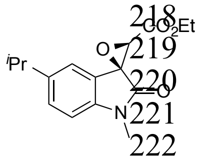

Following the above general procedure, *cis* diastereoisomer ***13c*** was obtained as a pale yellow solid in 52% yield after purification by flash chromatography on silica gel (nHexane/EtOAc=8/2), m.p. 120-122 °C. IR ($CHCl_3$): $\tilde{\nu}$ = 3025, 3011, 2959, 1739, 1727, 1625, 1494, 1471, 1369, 1346 cm^{-1}. ^{1}H NMR ($CDCl_3$, 300 MHz, 25 °C): δ (ppm) 1.22 (d, *J* = 6.8 Hz, 6H, $(CH_3)_2CHC_{arom}$); 1.36 (t, *J* = 7.1 Hz, 3H, CH_3CH_2); 2.80-2.95 (m, 1H, $(CH_3)_2CHC_{arom}$); 3.23 (s, 3H, NCH_3); 4.18 (s, 1H, OCH); 4.30-4.40 (m, 3H, CH_3CH_2O, OCH); 6.83 (d, *J* = 7.9 Hz, 1H, CH_{arom}); 6.98 (s, 1H, CH_{arom}); 7.28 (d, *J* = 7.9 Hz, 1H, CH_{arom}). ^{13}C NMR ($CDCl_3$, 75 MHz, 25 °C): δ (ppm) 14.2, 24.2 (2xC), 26.8, 33.9, 60.2, 60.3, 62.1, 109.0, 120.5, 121.1, 129.2, 143.2, 144.3, 164.9, 168.9. HRMS: exact mass calculated for ($C_{16}H_{19}NNaO_4$) requires *m*/*z* 312.1212, found *m*/*z* 312.1215. Chiral-phase HPLC analysis: [Daicel Chiralpack IC 5μ, λ=254 nm, *n*Hexane/EtOH=70/30, flow rate 1.0mL/min]: T_{major} = 13.73 min, T_{minor} = 15.19 min *er* = 64:36. [α]$_D$ = -33 (*c* = 0.0250 g/cm^3 in $CHCl_3$).

(2′R,3′R)-ethyl 5,7-dichloro-1-methyl-2-oxospiro[indoline-3,2′-oxirane]-3′- carboxylate ***12d***

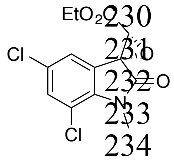

Following the above general procedure, *trans* diastereoisomer ***12d*** was obtained as a pale red solid in 29% yield after purification by flash chromatography on silica gel (nHexane/EtOAc=8/2), m.p. 124-126 °C. IR ($CHCl_3$): $\tilde{\nu}$ = 3031, 3006, 1740, 1729, 1578, 1463, 1337, 1309 cm^{-1}. ^{1}H NMR ($CDCl_3$, 300 MHz, 25 °C): δ (ppm) 1.31 (t, *J* = 7.1 Hz, 3H, CH_3CH_2O); 3.62 (s, 3H, NCH_3); 4.18-4.40 (m, 3H, CH_3CH_2O, OCH); 7.34 (s, 1H, CH_{arom}); 7.40 (s, 1H, CH_{arom}). ^{13}C NMR ($CDCl_3$, 75 MHz, 25 °C): δ (ppm) 14.3, 30.5, 59.4, 60.6, 62.8, 117.0, 123.6, 124.0, 129.0, 132.9, 140.2, 165.1, 170.1. HRMS: exact mass calculated for ($C_{13}H_{11}Cl_2NNaO_4$) requires *m*/*z* 337.9963, found *m*/*z* 337.9961. Chiral-phase HPLC analysis: [Daicel Chiralpack IC 5μ, λ=254 nm, nHexane/EtOH=70/30, flow rate 1.0mL/min]: T_{major} = 11.74 min, T_{minor} = 9.48 min *er* = 93:7. [α]$_D$ = -96 (c = 0.0160 g/cm^3 in $CHCl_3$).

(2′S,3′R)-ethyl 5,7-dichloro-1-methyl-2-oxospiro[indoline-3,2′-oxirane]-3′- carboxylate ***13d***

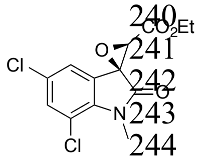

Following the above general procedure, *cis* diastereoisomer **13d** was obtained as a pale red solid in 50% yield after purification by flash chromatography on silica gel (nHexane/EtOAc=8/2), m.p. 194-196 °C. IR ($CHCl_3$): $\tilde{\nu}$ = 3034, 3003, 1759, 1740, 1583, 1463, 1334, 1306 cm^{-1}. ^1H NMR ($CDCl_3$, 300 MHz, 25 °C): δ (ppm) 1.36 (t, *J* = 7.1 Hz, 3H, CH_3CH_2O); 3.59 (s, 3H, NCH_3); 4.12 (s, 1H, OCH); 4.35 (q, *J* = 7.1 Hz, 2H, CH_3CH_2O); 6.97 (s, 1H, CH_{arom}); 7.35 (s, 1H, CH_{arom}). ^{13}C NMR ($CDCl_3$, 75 MHz, 25 °C): δ (ppm) 14.2, 30.2, 59.2, 60.7, 62.5, 117.1, 121.4, 125.3, 128.9, 132.8, 139.7, 164.0, 168.8. HRMS: exact mass calculated for ($C_{13}H_{11}Cl_2NNaO_4$) requires *m/z* 337.9963, found *m/z* 337.9961. Chiral-phase HPLC analysis: [Daicel Chiralpack IC 5μ, λ=254 nm, nHexane/EtOH=70/30, flow rate 1.0mL/min]: T_{major} = 21.69 min, T_{minor} = 20.20 min *er* = 77:23. $[\alpha]_D$ = -42 (*c* = 0.0220 g/cm³ in $CHCl_3$).

(2′R,3′R)-ethyl 5-methoxy-1-methyl-2-oxospiro[indoline-3,2′-oxirane]-3′-carboxylate ***12e***

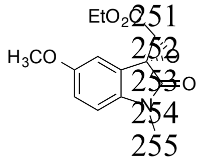

Following the above general procedure, *trans* diastereoisomer **12e** was obtained as a pale yellow solid in 50% yield after purification by flash chromatography on silica gel (nHexane /EtOAc = 8/2), m.p. 105-107 °C. IR ($CHCl_3$): $\tilde{\nu}$ = 1735, 1734, 1614, 1492, 1467, 1358, 1260 cm^{-1}. ^1H NMR ($CDCl_3$, 300MHz, 25 °C): δ (ppm) 1.28 (t, *J* = 7.1 Hz, 3H, CH_3CH_2O); 3.23 (s, 3H, NCH_3); 3.75 (s, 3H, OCH_3); 4.18 (s, 1H, OCH); 4.22-4.25 (m, 2H, CH_3CH_2O); 6.80 (d, *J* = 8.5 Hz, 1H, CH_{arom}); 6.92 (d, *J* = 8.5 Hz, 1H, CH_{arom}); 7.09 (s, 1H, CH_{arom}). ^{13}C NMR ($CDCl_3$, 75 MHz, 25 °C): δ (ppm) 14.3, 26.9, 56.0, 59.9, 62.3, 66.1, 109.5, 112.3, 115.9, 120.5, 139.1, 156.3, 165.8, 169.8 ppm. HRMS: exact mass calculated for ($C_{14}H_{15}NNaO_4$) requires *m/z* 300.0848, found *m/z* 300.0844. HPLC analysis: [Daicel Chiralpack IC 5μ, λ=254 nm, nHeptane/EtOH=70/30, flow rate 1.0mL/min]: T_{major} = 16.33 min, T_{minor} = 11.45 min *er* = 90:10. $[\alpha]_D$ = -10 (c = 0.0451 g/cm³ in $CHCl_3$).

(2′S,3′R)-ethyl 5-methoxy-1-methyl-2-oxospiro[indoline-3,2′-oxirane]-3′-carboxylate ***13e***

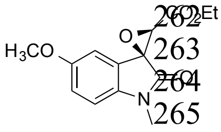

Following the above general procedure, *cis* diastereoisomer ***13e*** was obtained as a pale yellow solid in 50% yield after purification by flash chromatography on silica gel (nHexane/EtOAc = 8/2), m.p. 121-123 °C. IR ($CDCl_3$): $\tilde{\nu}$ = 1763, 1603, 1502, 1367, 1290 cm^{-1}.^1H NMR ($CDCl_3$, 300 MHz, 25 °C): δ (ppm) 1.34 (t, *J* = 7.2 Hz, 3H, CH_3CH_2O); 3.18 (s, 3H, NCH_3); 3.75 (s, 3H, OCH_3); 4.12 (s, 1H, OCH); 4.33 (q, *J* = 7.2 Hz, 2H, CH_3CH_2O); 6.69 (s, 1H, CH_{arom}); 6.69 (d, *J* = 8.5 Hz, 1H, CH_{arom}); 6.90 (d, *J* = 8.5 Hz, 1H, CH_{arom}). ^{13}C NMR ($CDCl_3$, 75 MHz, 25 °C): δ (ppm) 14.1, 26.7, 56.0, 60.3, 62.1, 65.8, 109.4, 109.7, 116,4, 122.4, 138.6, 156.5, 164.8, 168.6. HRMS: exact mass calculated for ($C_{14}H_{15}NNaO_5$) requires *m/z* 300.0848, found *m/z* 300.0846. HPLC analysis: [Daicel Chiralpack IC 5μ, λ=254 nm, *n*Heptane/EtOH=70/30, flow rate 1.0mL/min]: T_{major} = 16.35 min, T_{minor} = 19.33 min *er* = 62:38. $[\alpha]_D$ = -4 (*c* = 0.0390 g/cm³ in $CHCl_3$).

(2′R,3′R)-ethyl 1-methyl-5-nitro-2-oxospiro[indoline-3,2′-oxirane]-3′- carboxylate ***12f***

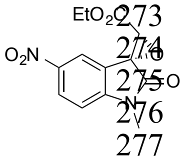

Following the above general procedure, *trans* diastereoisomer **12f** was obtained as a pale yellow solid in 19% yield after purification by flash chromatography on silica gel (DCM 100%), m.p. 170-172 °C. IR ($CHCl_3$): $\tilde{\nu}$ = 3031, 3017, 3009, 1754, 1743, 1617, 1533, 1494, 1340 cm^{-1}. ^1H NMR ($CDCl_3$, 300 MHz, 25 °C): δ (ppm) 1.34 (t, *J* = 7.1 Hz, 3H, CH_3CH_2O); 3.35 (s, 3H, NCH_3); 4.23-4.42 (m, 3H, CH_3CH_2O, OCH); 7.03 (d, *J* = 8.9 Hz, 1H, CH_{arom}); 8.37 (d, *J* = 7.4 Hz, 2H, CH_{arom}). ^{13}C NMR ($CDCl_3$, 75 MHz, 25 °C): δ (ppm) 14.2, 27.4, 59.4, 60.1, 62.9, 108.9, 120.4, 121.1, 128.0, 143.8, 150.9, 165.1, 170.1. HRMS: exact mass calculated for ($C_{13}H_{12}N_2NaO_6$) requires *m/z* 315.0593, found *m/z* 315.0596. Chiral-phase HPLC analysis: [Daicel Chiralpack IC 5μ, λ=254 nm, nHexane/EtOH=70/30, flow rate 1.0mL/min]: T_{major} = 23.13 min, T_{minor} = 22.02 min *er* = 85:15. $[\alpha]_D$ = -29 (c = 0.0180 g/cm³ in $CDCl_3$).

(2′S,3′R)-ethyl 1-methyl-5-nitro-2-oxospiro[indoline-3,2′-oxirane]-3′-carboxylate ***13f***

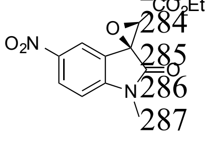

Following the above general procedure, *cis* diastereoisomer ***13f*** was obtained as a pale yellow solid in 53% yield after purification by flash chromatography on silica gel (DCM 100%), m.p. 168-170 °C. IR ($CHCl_3$): $\tilde{\nu}$ = 3029, 3017, 3009, 1754, 1729, 1630, 1527, 1494, 1463, 1340 cm^{-1}. ^1H NMR ($CDCl_3$, 300 MHz, 25 °C): δ (ppm) 1.37 (t, *J* = 7.1 Hz, 3H, CH_3CH_2O); 3.33 (s, 3H, NCH_3); 4.29 (s, 1H, OCH); 4.36 (dq, *J* = 9.2 Hz, 7.1 Hz, 1H,

CH_3CHHO); 4.38 (dq, *J* = 9.2 Hz, 7.1 Hz, 1H, CH_3CHHO); 7.03 (d, *J* = 8.9 Hz, 1H, CH_{arom}); 8.02 (d, *J* = 1.9 Hz, 1H, CH_{arom}); 8.39 (dd, *J* = 8.7 Hz, 2.2 Hz, 1H, CH_{arom}). ^{13}C NMR ($CDCl_3$, 75 MHz, 25 °C): δ (ppm) 14.1, 27.3, 59.2, 60.4, 62.5, 109, 118.5, 122.2, 128.1, 143.2, 150.5, 163.9, 169.1. HRMS: exact mass calculated for ($C_{13}H_{12}N_2NaO_6$) requires *m*/*z* 315.0593, found *m*/*z* 315.0596. Chiral-phase HPLC analysis: [Daicel Chiralpack IB 5μ, λ=254 nm, *n*Heptane/EtOH/DEA=70/30/0.1, flow rate 1.0mL/min]: T_{major} = 12.89 min, T_{minor} = 14.80 min *er* = 62:38. $[\alpha]_D$ = -29 (*c* = 0.0160 g/cm³ in $CHCl_3$).

(2′R,3′R)-ethyl 1-methyl-2-oxo-1,2-dihydrospiro[benzo[g]indole-3,2′-oxirane]-3′- carboxylate ***12g***

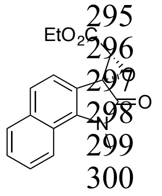

Following the above general procedure, *trans* diastereoisomer **12g** was obtained as a pale red solid in 50% yield after purification by flash chromatography on silica gel (*n*Hexane/EtOAc=8/2), m.p. 185-187 °C. ^1H NMR ($CDCl_3$, 300 MHz, 25 °C): δ (ppm) 1.26-1.30 (m, 3H, CH_3CH_2O); 3.88 (s, 3H, NCH_3); 4.21-4.31 (m, 3H, OCH, CH_3CH_2O), 7.50-7.55 (m, 4H, CH_{arom}); 7.86-7.88 (m, 1H, CH_{arom}), 8.40-8.43 (m, 1H, CH_{arom}). ^{13}C NMR ($CDCl_3$, 75 MHz, 25 °C): δ (ppm) 14.3, 28.3, 59.2, 62.5, 63.6, 109.2, 118.5, 122.2, 123.7, 124.7, 126.6, 128.1, 129.2, 142.3, 149.3, 168.6, 170.1. HRMS: exact mass calculated for ($C_{17}H_{15}NNaO_4$) requires *m*/*z* 320.0899, found *m*/*z* 320.0896. Chiral-phase HPLC analysis: [Daicel Chiralpack IC 5μ, λ=254 nm, *n*Hexane/EtOH=70/30, flow rate 1.0mL/min]: T_{major} = 18.48 min, T_{minor} = 13.56 min *er* = 91:9. $[\alpha]_D$ = -20.15 (*c* = 0.0059 g/cm³ in $CHCl_3$).

(2′S,3′R)-ethyl 1-methyl-2-oxo-1,2-dihydrospiro[benzo[g]indole-3,2′-oxirane]-3′- carboxylate ***13g***

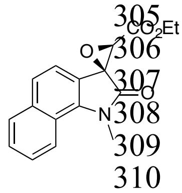

Following the above general procedure, *cis* diastereoisomer **13g** was obtained as a pale red solid in 48% yield after purification by flash chromatography on silica gel (*n*Hexane/EtOAc=8/2), m.p. 199-200 °C. ^1H NMR ($CDCl_3$, 300 MHz, 25 °C): δ (ppm) 1.39 (t, *J* = 7.0 Hz, 3H, CH_3CH_2O); 3.83 (s, 3H, NCH_3); 4.27 (s, 1H, OCH), 4.39 (q, *J* = 7.0 Hz, 2H, CH_3CH_2O), 7.16 (d, *J* = 8.1 Hz, 1H, CH_{arom}), 7.51-7.54 (m, 1H, CH_{arom}); 7.60 (d, *J* = 8.2 Hz, 1H, CH_{arom}), 7.87-7.90 (m, 1H, CH_{arom}); 8.38-8.41 (m, 1H, CH_{arom}). ^{13}C NMR ($CDCl_3$, 75 MHz, 25 °C): δ (ppm) 14.1, 28.9, 61.3, 63.5, 66.7, 113.3, 121.1, 122.8, 123.9, 126.2, 126.6, 127.6, 128.7, 139.3, 145.3, 166.4, 170.1. HRMS: exact mass calculated for ($C_{17}H_{15}NNaO_4$) requires m/z 320.0899, found m/z 320.0902. Chiral-phase HPLC analysis: [Daicel Chiralpack IB 5μ, λ=254 nm, *nH*eptane/EtOH/DEA=70/30/0.1, flow rate 1.0mL/min]: T_{major} = 8.91 min, T_{minor} = 9.71 min *er* = 55:45.

(2′S,3′S)- diethyl 1-methyl-2-oxospiro[indoline-3,2′-oxiran]-3′-yl)phosphonate ***12h***

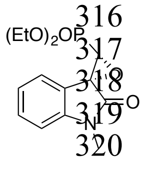

Following the above general procedure, *trans* diastereoisomer **12h** was obtained as a pale yellow solid in 45% yield after purification by flash chromatography on silica gel (nHexane/EtOAc=4/6), m.p. 166-168 °C. IR ($CHCl_3$): $\tilde{\nu}$ = 1725, 1236 cm⁻¹. ^1H NMR ($CDCl_3$, 300 MHz, 25 °C): δ (ppm) 1.21 [t, *J* = 7.0 Hz, 3H, $(CH_3CH_2O)_2P$], 1.41 [t, *J* = 7.1 Hz, 3H, $(CH_3CH_2O)_2P$], 3.26 (s, 3H, NCH_3), 3.73 (d, J_{HP} = 27.6 Hz, 1H, OCH), 3.95–4.14 [m, 2H, $(CH_3CH_2O)_2P$], 4.20–4.37 [m, 2H, $(CH_3CH_2O)_2P$], 6.90 (d, *J* = 7.9 Hz, 1H, CH_{arom}), 7.1 (dt, *J* = 7.7, 1.0 Hz, 1H, CH_{arom}), 7.39 (dt, *J* = 7.9, 1.3 Hz, 1H, CH_{arom}), 7.99 (d, *J* = 7.7 Hz, 1H, CH_{arom}). ^{13}C NMR ($CDCl_3$, 75 MHz, 25 °C): δ (ppm) 15.9 (d, J_{CCOP} = 5.8 Hz), 16.0 (d, J_{CCOP} = 5.7 Hz), 26.3, 55.5 (d, J_{CP} = 203.5 Hz), 59.7, 62.7 (d, J_{COP} = 6.3 Hz), 63.1 (d, J_{COP} = 6.1 Hz), 108.4, 118.9, 122.6, 126.5, 130.6, 145.3, 170.2. HRMS: exact mass calculated for ($C_{14}H_{18}NNaO_5P$) requires *m*/*z* 334.0820, found *m*/*z* 334.0824. Chiral-phase HPLC analysis: [Daicel Chiralpack IB 5μ, λ=254 nm, nHexane/iPrOH=9/1, flow rate 1.0mL/min]: T_{major} = 42.20 min, T_{minor} = 23.60 min *er* = 80:20. $[\alpha]_D$ = -2 (c = 0.0101 g/cm³ in $CHCl_3$).

(2′S,3′R)- diethyl 1-methyl-2-oxospiro[indoline-3,2′-oxiran]-3′-yl phosphonate ***13h***

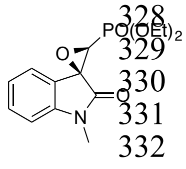

Following the above general procedure, *cis* diastereoisomer **13h** was obtained as a pale yellow solid in 35% yield after purification by flash chromatography on silica gel (*n*Hexane/EtOAc=4/6), m.p. 179-182 °C. ^1H NMR ($CDCl_3$, 300 MHz, 25 °C): δ (ppm) 1.35–1.43 [m, 6H, $(CH_3CH_2O)_2P$], 3.26 (s, 3H, NCH_3), 3.71 (d, J_{HP} = 27.4 Hz, 1H, OCH), 4.18–4.48 [m, 4H, $(CH_3CH_2O)_2P$], 6.89 (d, *J* = 7.9 Hz, 1H, CH_{arom}), 7.04–7.08 (m, 2H, CH_{arom}), 7.35–7.44 (m, 1H, CH_{arom}). ^{13}C NMR ($CDCl_3$, 75 MHz, 25 °C): δ (ppm) 16.0 (d, J_{CCOP} = 5.8 Hz), 16.3 (d, J_{CCOP} = 5.7 Hz), 27.3, 57.5 (d, J_{CP} = 203.5 Hz), 58.4, 62.5 (d, J_{COP} = 6.3 Hz), 73.1 (d, J_{COP} = 6.1 Hz), 107.9, 117.7, 124.1, 126.1, 131.5, 147.2, 168.1 ppm. HRMS: exact mass calculated for ($C_{14}H_{18}NNaO_5P$) requires *m*/*z* 334.0820, found *m*/*z* 334.0818. Chiral-phase HPLC analysis: [Daicel Chiralpack IB 5μ, λ=254 nm, *n*Hexane/*i*PrOH=9/1, flow rate 1.0mL/min]: T_{major} = 16.50 min, T_{minor} = 12.80 min *er* = 61:39. $[\alpha]_D$ = -19.45 (*c* = 0.0098 g/cm³ in $CHCl_3$).

(2′S,3′S)- diethyl 1-phenyl-2-oxospiro[indoline-3,2′-oxiran]-3′-yl phosphonate ***12i***

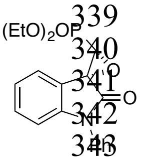

Following the above general procedure, *trans* diastereoisomer ***12i*** was obtained as a pale yellow solid in 57% yield after purification by flash chromatography on silica gel (nHexane/EtOAc=1/1), m.p. 197-199 °C. IR ($CHCl_3$): $\tilde{\nu}$ = 1716, 1265 cm^{-1}. ^1H NMR ($CDCl_3$, 300 MHz, 25 °C): δ (ppm) 1.18 [t, *J* = 7.1 Hz, 3H, $(CH_3CH_2O)_2P$], 1.34 [t, *J* =7.1 Hz, 3H, $(CH_3CH_2O)_2P$], 3.76 (d, J_{HP} = 27.6 Hz, 1H, OCH), 4.00–4.08 [m, 2H, $(CH_3CH_2O)_2P$], 4.18–4.33 [m, 2H, $(CH_3CH_2O)_2P$], 6.77 (d, *J* = 7.6 Hz, 1H, CH_{arom}), 7.05 (t, *J* = 7.6 Hz, 1H, CH_{arom}), 7.22 (t, *J* = 7.6 Hz, 1H, CH_{arom}), 7.35–7.46 (m, 5H, CH_{arom}), 7.96 (d, *J* = 7.6 Hz, 1H, CH_{arom}). ^{13}C NMR ($CDCl_3$, 75 MHz, 25 °C): δ (ppm) 16.0 (d, J_{CCOP} = 5.7 Hz), 16.2 (d, J_{CCOP} = 5.8 Hz), 58.1 (d, J_{CP} = 203.7 Hz), 60.1, 62.9 (d, J_{COP} = 6.2 Hz), 63.4 (d, J_{COP} = 6.1 Hz), 109.8, 118.8, 123.3, 126.1, 126.9, 128.3, 129.5, 130.6, 133.5, 145.5, 169.8 ppm. HRMS: exact mass calculated for ($C_{19}H_{20}NNaO_5P$) requires *m/z* 396.0977, found *m/z* 396.0975. Chiral-phase HPLC analysis: [Daicel Chiralpack IB 5μ, λ=254 nm, nHexane/iPrOH=9/1, flow rate 1.0mL/min]: T_{major} = 39.90 min, T_{minor} = 17.50 min *er* = 79:21. $[\alpha]_D$ = -6.7 (c = 0.0122 g/cm^3 in $CHCl_3$).

(2′S,3′R)- diethyl 1-phenyl-2-oxospiro[indoline-3,2′-oxiran]-3′-yl phosphonate ***13i***

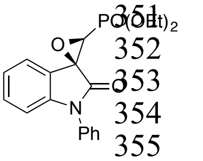

Following the above general procedure, *cis* diastereoisomer ***13i*** was obtained as a pale-yellow solid in 36% yield after purification flash chromatography on silica gel (*n*Hexane/EtOAc=1/1), m.p. 211-213 °C. ^1H NMR ($CDCl_3$, 300 MHz, 25 °C): δ (ppm) 1.33–1.42 [m, 6H, $(CH_3CH_2O)_2P$], 3.79 (d, J_{HP} = 27.3 Hz, 1H, OCH), 4.18–4.45 [m, 4H, $(CH_3CH_2O)_2P$], 6.86 (d, *J* = 7.9 Hz, 1H, CH_{arom}), 7.05– 7.14 (m, 2H, CH_{arom}), 7.28–7.39 (m, 1H, CH_{arom}), 7.40–7.57 (m, 5H, CH_{arom}). ^{13}C NMR ($CDCl_3$, 75 MHz, 25 °C): δ (ppm) 16.2 (d, J_{CCOP} = 5.7 Hz), 16.4 (d, J_{CCOP} = 5.8 Hz), 58.6 (d, J_{CP} = 203.7 Hz), 60.1, 63.3 (d, J_{COP} = 6.2 Hz), 63.6 (d, J_{COP} = 6.1 Hz), 108.8, 117.6, 123.3, 126.0, 126.9, 128.4, 129.5, 130.6, 133.5, 146.2, 169.8 ppm. HRMS: exact mass calculated for ($C_{19}H_{20}NNaO_5P$) requires *m/z* 396.0977, found *m/z* 396.0975. Chiral-phase HPLC analysis: [Daicel Chiralpack IB 5μ, λ=254 nm, *n*Hexane/*i*PrOH=9/1, flow rate 1.0mL/min]: T_{major} = 12.40 min, T_{minor} = 9.29 min *er* = 62:38. $[\alpha]_D$ = -20.5 (*c* = 0.0047 g/cm^3 in $CHCl_3$).

(2′S,3′S)- diethyl 1-benzyl-2-oxospiro[indoline-3,2′-oxiran]-3′-yl phosphonate ***12j***

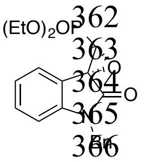

Following the above general procedure, *trans* diastereoisomer **12j** was obtained as a yellow solid in 45% yield after purification by flash chromatography on silica gel (nHexane/EtOAc=1/1), m.p. 205-207 °C. IR ($CHCl_3$): $\tilde{\nu}$ = 1731, 1262 cm^{-1}. ^1H NMR ($CDCl_3$, 300 MHz, 25 °C): δ (ppm) 1.22 [t, *J* = 7.1 Hz, 3H, $(CH_3CH_2O)_2P$], 1.43 [t, *J* =7.1 Hz, 3H, $(CH_3CH_2O)_2P$], 3.81 (d, J_{HP} = 27.4 Hz, 1H, OCH), 4.02–4.14 [m, 2H, $(CH_3CH_2O)_2P$], 4.24–4.39 [m, 2H, $(CH_3CH_2O)_2P$], 4.96 (s, 2H, CH_2N), 6.81 (d, *J* = 7.6 Hz, 1H, CH_{arom}), 7.07 (t, *J* = 7.6 Hz, 1H, CH_{arom}), 7.24–7.36 (m, 6H, CH_{arom}), 8.00 (d, *J* = 7.6 Hz, 1H, CH_{arom}). ^{13}C NMR ($CDCl_3$, 75 MHz, 25 °C): δ (ppm) 16.2 (d, J_{CCOP} = 5.7 Hz), 16.4 (d, J_{CCOP} = 5.8 Hz),44.4, 58.1 (d, J_{CP} = 203.1 Hz), 60.2 (d, J_{CCP} = 1.1 Hz), 62.9 (d, J_{COP} = 6.0 Hz), 63.3 (d, J_{COP} = 6.0 Hz), 109.3, 119.2, 122.1, 127.3, 127.5, 127.7, 128.7, 131.7, 135.2, 145.5, 170.7 ppm. HRMS: exact mass calculated for ($C_{20}H_{22}NNaO_5P$) requires *m/z* 410.1133, found *m/z* 410.1130. Chiral-phase HPLC analysis: [Daicel Chiralpack IB 5μ, λ=254 nm, *n*Hexane/*i*PrOH=9/1, flow rate 1.0mL/min]: T_{major} = 39.90 min, T_{minor} = 17.50 min *er* = 74:26. $[\alpha]_D$ = -6.7 (c = 0.0122 g/cm^3 in $CHCl_3$).

(2′S,3′R)- diethyl 1-benzyl-2-oxospiro[indoline-3,2′-oxiran]-3′-yl phosphonate ***13j***

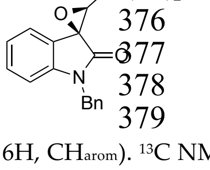

Following the above general procedure, *cis* diastereoisomer ***13j*** was obtained as a yellow solid in 35% yield after purification by flash chromatography on silica gel (*n*Hexane/EtOAc=1/1), m.p. 222-225 °C. ^1H NMR ($CDCl_3$, 300 MHz, 25 °C): δ (ppm) 1.33 [t, *J* = 7.1 Hz, 6H $(CH_3CH_2O)_2P$], 3.69 (d, J_{HP} = 27.6 Hz, 1H, OCH), 4.14–4.40 [m, 4H, $(CH_3CH_2O)_2P$], 4.90 (s, 2H, CH_2N), 6.71 (d, *J* = 7.8 Hz, 1H, CH_{arom}), 6.92–7.02 (m, 2H, CH_{arom}), 7.16–7.30 (m, 6H, CH_{arom}). ^{13}C NMR ($CDCl_3$, 75 MHz, 25 °C): δ (ppm) 16.2 (d, J_{CCOP} = 5.7 Hz), 16.4 (d, J_{CCOP} = 5.8 Hz),44.6, 57.9 (d, J_{CP} = 203.1 Hz), 60.7 (d, J_{CCP} = 1.1 Hz), 62.6 (d, J_{COP} = 6.0 Hz), 62.3 (d, J_{COP} = 6.0 Hz), 109.3, 119.2, 122.5, 127.4, 127.6, 127.7, 128.7, 131.7, 135.2, 145.5, 170.7 ppm. HRMS: exact mass calculated for ($C_{20}H_{22}NNaO_5P$) requires *m/z* 410.1133, found *m/z* 410.1130. Chiral-phase HPLC analysis: [Daicel Chiralpack IB 5μ, λ=254 nm, *n*Hexane/*i*PrOH=9/1, flow rate 1.0mL/min]: T_{major} = 10.00 min, T_{minor} = 12.10 min *er* = 55:45. $[\alpha]_D$ = -10.7 (*c* = 0.0053 g/cm^3 in $CHCl_3$).

(2′S,3′S)- diethyl 1-(2,4-dichlorobenzyl)-2-oxospiro[indoline-3,2′-oxiran]-3′-yl)phosphonate ***12k***

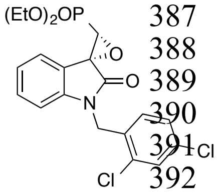

Following the above general procedure, *trans* diastereoisomer **12k** was obtained as a pale yellow solid in 54% yield after purification by flash chromatography on silica gel (nHexane/EtOAc=1/1), m.p. 235-237 °C. IR ($CHCl_3$): $\tilde{\nu}$ = 1733, 1253 cm^{-1}. ^{1}H NMR ($CDCl_3$, 300 MHz, 25 °C): δ (ppm) 1.23 [t, *J* = 7.1 Hz, 3H, $(CH_3CH_2O)_2P$], 1.42 [t, *J* =7.1 Hz, 3H, $(CH_3CH_2O)_2P$], 3.81 (d, J_{HP} = 27.4 Hz, 1H, OCH), 3.97–4.19 [m, 2H, $(CH_3CH_2O)_2P$], 4.22–4.39 [m, 2H, $(CH_3CH_2O)_2P$], 5.03 (s, 2H, CH_2N), 6.73 (d, *J* = 7.9 Hz, 1H, CH_{arom}), 7.05–7.20 (m, 3H, CH_{arom}), 7.30–7.34 (m, 3H, CH_{arom}), 7.43 (d, *J* = 1.9 Hz, 1H, CH_{arom}), 8.00 (d, *J* = 7.6 Hz, 1H, CH_{arom}). ^{13}C NMR ($CDCl_3$, 75 MHz, 25 °C): δ (ppm) 15.9 (d, J_{CCOP} = 5.6 Hz), 16.0 (d, J_{CCOP} = 5.5 Hz), 41.1, 57.9 (d, J_{CP} = 203.0 Hz), 59.7 (d, J_{CCP} = 1.1 Hz), 62.6 (d, J_{COP} = 6.2 Hz), 63.1 (d, J_{COP} = 6.2 Hz), 109.2, 118.9, 123.0, 126.8, 127.2, 128.8, 129.2, 130.6, 130.8, 133.2, 133.8, 144.0, 170.6 ppm. HRMS: exact mass calculated for ($C_{20}H_{20}Cl_2NNaO_5P$) requires *m/z* 478.0354, found *m/z* 478.0356. Chiral-phase HPLC analysis: [Daicel Chiralpack IB 5μ, λ=254 nm, *n*Hexane/*i*PrOH=9/1, flow rate 1.0mL/min]: T_{major} = 26.20 min, T_{minor} = 16.80 min *er* = 72:28. [α]$_D$ = -20.4 (c = 0.0091 g/cm^3 in $CHCl_3$).

(2′S,3′R)- diethyl 1-(2,4-dichlorobenzyl)-2-oxospiro[indoline-3,2′-oxiran]-3′-yl)phosphonate ***13k***

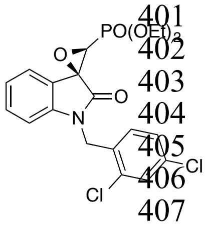

Following the above general procedure, *cis* diastereoisomer ***13k*** was obtained as a pale yellow solid in 29% yield after purification by flash chromatography on silica gel (*n*Hexane/EtOAc=1/1), m.p. 227-229 °C. ^{1}H NMR ($CDCl_3$, 300 MHz, 25 °C): δ (ppm) 1.50 [t, *J* = 7.1 Hz, 6H ($CH_3CH_2O)_2P$], 3.87 (d, J_{HP} = 27.6 Hz, 1H, OCH), 4.21–4.57 [m, 4H, $(CH_3CH_2O)_2P$], 5.15 (s, 2H, CH_2N), 6.83 (d, *J* = 7.2 Hz, 1H, CH_{arom}), 7.14–7.52 (m, 6H, CH_{arom}). ^{13}C NMR ($CDCl_3$, 75 MHz, 25 °C): δ (ppm) 16.2 (d, J_{CCOP} = 5.6 Hz), 16.4 (d, J_{CCOP} = 5.5 Hz), 41.1, 58.2 (d, J_{CP} = 203.0 Hz), 59.9 (d, J_{CCP} = 1.1 Hz), 62.8 (d, J_{COP} = 6.2 Hz), 63.5 (d, J_{COP} = 6.2 Hz), 109.2, 118.9, 123.0, 126.8, 127.2, 128.8, 129.2, 130.6, 130.8, 133.2, 133.8, 144.0, 170.4 ppm. HRMS: exact mass calculated for ($C_{20}H_{20}Cl_2NNaO_5P$) requires *m/z* 478.0354, found *m/z* 478.0357. Chiral-phase HPLC analysis: [Daicel Chiralpack IB 5μ, λ=254 nm, *n*Hexane/*i*PrOH=9/1, flow rate 1.0mL/min]: T_{major} = 11.00 min, T_{minor} = 9.90 min *ee* = 55:45. [α]$_D$ = -5.32 (*c* = 0.0064 g/cm^3 in $CHCl_3$).

(2′S,3′S)- diethyl 5-chloro-1-methyl-2-oxospiro[indoline-3,2′-oxiran]-3′-yl)phosphonate ***12l***

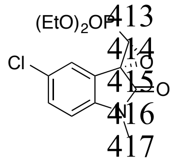

Following the above general procedure, *trans* diastereoisomer **12l** was obtained as a white solid in 38% yield after purification by flash chromatography on silica gel (nHexane/EtOAc=1/1), m.p. 211-215 °C. IR ($CHCl_3$): $\tilde{\nu}$ = 1734, 1260 cm^{-1}. ^{1}H NMR ($CDCl_3$, 300 MHz, 25 °C): δ (ppm) 1.26 [t, *J* = 7.0 Hz, 3H, $(CH_3CH_2O)_2P$], 1.42 [t, *J* =7.0 Hz, 3H, $(CH_3CH_2O)_2P$], 3.26 (s, 3H, NCH_3); 3.73 (d, J_{HP} = 26.6 Hz, 1H, OCH), 4.05–4.18 [m, 2H, $(CH_3CH_2O)_2P$], 4.25–4.34 [m, 2H, $(CH_3CH_2O)_2P$], 6.83 (d, *J* = 8.3 Hz, 1H, CH_{arom}), 7.38 (d, *J* = 8.3 Hz, 1H, CH_{arom}), 8.02 (s, 2H, CH_{arom}). ^{13}C NMR ($CDCl_3$, 75 MHz, 25 °C): δ (ppm) 16.4 (d, J_{CP} = 5.9 Hz), 16.6 (d, J_{CP} = 5.6 Hz), 27.1, 58.1 (d, J_{CP} = 203.4 Hz), 59.9, 63.3 (d, J_{CP} = 6.3 Hz), 63.8 (d, J_{CP} = 6.1 Hz), 109.8, 121.2, 127.5, 128.8, 131.0, 144.3, 170.4. HRMS: exact mass calculated for ($C_{14}H_{17}ClNNaO_5P$) requires *m/z* 368.0431, found *m/z* 368.0433. Chiral-phase HPLC analysis: [Daicel Chiralpack IC 5μ, λ=254 nm, nHexane/EtOH=9/1, flow rate 1.0mL/min]: T_{major} = 18.92 min, T_{minor} = 17.56 min *er* = 77:23. [α]$_D$ = +4 (c = 0.0170 g/cm^3 in $CHCl_3$).

(2′S,3′R)- diethyl 5-chloro-1-methyl-2-oxospiro[indoline-3,2′-oxiran]-3′-yl)phosphonate ***13l***

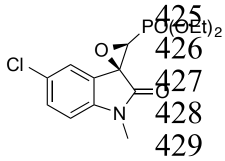

Following the above general procedure, *cis* diastereoisomer **13l** was obtained as a pale yellow solid in 58% yield after purification by flash chromatography on silica gel (nHexane/EtOAc=1/1), m.p. 229-231 °C. IR ($CHCl_3$): $\tilde{\nu}$ = 1752, 1258 cm^{-1}. ^{1}H NMR ($CDCl_3$, 300 MHz, 25 °C): δ (ppm) 1.59-1.65 [m, 6H $(CH_3CH_2O)_2P$], 3.48 (s, 3H, NCH_3); 3.92 (d, J_{HP} = 27.0 Hz, 1H, OCH), 4.44–4.68 [m, 4H, $(CH_3CH_2O)_2P$], 7.05 (d, *J* = 7.9 Hz, 1H, CH_{arom}), 7.50–7.60 (m, 2H, CH_{arom}) ppm. ^{13}C NMR ($CDCl_3$, 75 MHz, 25 °C): δ (ppm) 16.2 (t, J_{CP} = 6.3 Hz), 16.9 (d, J_{CP} = 5.6 Hz),27.0, 60.0 (d, J_{CP} = 200.7 Hz), 60.6, 62.8 (d, J_{CP} = 6.5 Hz), 64.4 (d, J_{CP} = 6.6 Hz), 122.5, 125.8 (d, J_{CP} = 27.0 Hz), 126.5 (d, J_{CP} = 9.2 Hz), 128.2 (d, J_{CP} = 20.0 Hz), 131.0, 143.7, 168.5. HRMS: exact mass calculated for ($C_{14}H_{17}ClNNaO_5P$) requires *m/z* 368.0431, found *m/z* 368.0433. Chiral-phase HPLC analysis: [Daicel Chiralpack IC 5μ, λ=254 nm, nHexane/EtOH=85/15, flow rate 1.0mL/min]: T_{major} = 32.17 min, T_{minor} = 30.15 min *er* = 57:43. [α]$_D$ = -1 (c = 0.0060 g/cm^3 in $CHCl_3$).

(2'S,3'S)-2-oxospiro[indoline-3,2'-oxirane]-3'-carboxylate ***12m***

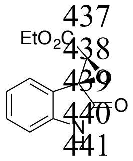

Following the above general procedure, *trans* diastereoisomer **12m** was obtained as a white solid in 62% yield after purification by flash chromatography on silica gel (nHexane/EtOAc=7/3). IR ($CHCl_3$): $\tilde{\nu}$ = 3433, 3035, 3009, 1751, 1726, 1622, 1474, 1340, 1318 cm^{-1}. ^1H NMR ($CDCl_3$, 300 MHz, 25 °C): δ (ppm) 1.29 (t, *J* = 7.1 Hz, 3H, CH_3CH_2O); 4.17 – 4.47 (m, 3H, CH_3CH_2O, OCH); 6.87–7.17 (m, 2H, CH_{arom}); 7.27 – 7.51 (m, 2H, CH_{arom}); 9.38 (s, 1H, NH). ^{13}C NMR ($CDCl_3$, 75 MHz, 25 °C): δ (ppm) 14.3, 59.9, 60.5, 62.5, 111.4, 119.6, 123.3, 125.2, 131.3, 143.0, 165.7, 172.6. HRMS: exact mass calculated for ($C_{12}H_{11}NNaO_4$) requires *m/z* 256.0586, found *m/z* 256.0582. Chiral-phase HPLC analysis: [Daicel Chiralpack IC 5μ, λ=254 nm, nHexane/EtOH=90/10, flow rate 1.0mL/min]: T_{major} = 6.28 min, T_{minor} = 5.51 min *er* = 75:25. $[\alpha]_D$ = -84.22 (c = 0.0155 g/cm^3 in $CHCl_3$).

(2'R,3'S)-2-oxospiro[indoline-3,2'-oxirane]-3'-carboxylate ***13m***

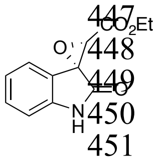

Following the above general procedure, *cis* diastereoisomer **13m** was obtained as a white solid in 34% yield after purification by flash chromatography on silica gel (nHexane/EtOAc=7/3). IR ($CHCl_3$): $\tilde{\nu}$ = 3434, 3026, 3009, 1759, 1734, 1723, 1620, 1469, 1337 cm^{-1}. ^1H NMR ($CDCl_3$, 300 MHz, 25 °C): δ (ppm) 1.33 (t, *J* = 6,8 Hz, 3H, CH_3CH_2O); 4.18 (s, OCH); 4.34 (q, *J* = 6.8 Hz, 2H, CH_3CH_2O); 6.91 – 7.13 (m, 3H, CH_{arom}); 7.28 – 7.40 (m, 1H, CH_{arom}); 9.19 (s, 1H, NH). ^{13}C NMR ($CDCl_3$, 75 MHz, 25 °C): δ (ppm) 14.3, 29.8, 60.4, 62.2, 111.5, 121.5, 122.7, 123.2, 131.4, 142.6, 164.7, 171.4. HRMS: exact mass calculated for ($C_{12}H_{11}NNaO_4$) requires *m/z* 256.0586, found *m/z* 256.0589. Chiral-phase HPLC analysis: [Daicel Chiralpack IC 5μ, λ=254 nm, nHexane/EtOH=90/10, flow rate 1.0mL/min]: T_{major} = 16.35 min, T_{minor} = 15.61 min *er*= 61:39. $[\alpha]_D$ = -22.78 (c = 0.0155 g/cm^3 in $CHCl_3$).

(2'S,3'S)-ethyl 5-fluoro-2-oxospiro[indoline-3,2'-oxirane]-3'-carboxylate ***12n***

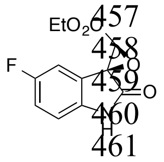

Following the above general procedure, *trans* diastereoisomer **12n** was obtained as a white solid in 32% yield after purification by flash chromatography on silica gel (nHexane/EtOAc=7/3). IR ($CHCl_3$): $\tilde{\nu}$ = 3429, 3207, 3031, 2979, 1715, 1767, 1752, 1630, 1481, 1319, 1231, 1204 cm^{-1}. ^1H NMR ($CDCl_3$, 300 MHz, 25°C): δ (ppm) 1.32 (t, 3H, *J* = 7.1 Hz, CH_3CH_2O); 4.19 (s, 1H, OCH); 4.26-4.36 (m, 2H, CH_3CH_2O); 6.88 (m, 1H, CH_{arom}); 7.06 (t, 1H, *J* = 8.4 Hz, CH_{arom}); 7.24 (m, 1H, CH_{arom}); 8.66 (s, 1H, NH). ^{13}C NMR ($CDCl_3$, 75 MHz, 25 °C): δ (ppm) 14.3, 60.0, 60.5, 62.7, 111.9 (d, J_{CF} = 7.9 Hz), 113.5 (d, J_{CF} = 26.7 Hz), 117.9 (d, J_{CF} = 23.9 Hz), 121.3 (d, J_{CF} = 9.1 Hz), 138.8, 159.3 (d, J_{CF} = 242.2 Hz), 165.4, 172.3. HRMS: exact mass calculated for ($C_{12}H_{10}FNNaO_4$) requires *m/z* 274.0492, found *m/z* 274.0497. Chiral-phase HPLC analysis: [Daicel Chiralpack IC 5μ, λ=254 nm, nHexane/EtOH=90/10, flow rate 1.0mL/min]: T_{major} = 10.44 min, T_{minor} = 9.05 min *er* = 93:7. $[\alpha]_D$ = -132 (c = 0.0155 g/cm^3 in $CHCl_3$).

(2'R,3'S)-ethyl 5-fluoro-2-oxospiro[indoline-3,2'-oxirane]-3'-carboxylate, ***13n***

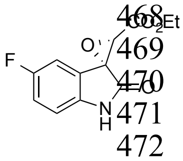

Following the above general procedure, *cis* diastereoisomer **13n** was obtained as a white solid in 58% yield after purification by flash chromatography on silica gel (nHexane/EtOAc=7/3). IR ($CHCl_3$): $\tilde{\nu}$ = 3429, 3207, 3031, 2979, 1715, 1767, 1752, 1630, 1481, 1319, 1231, 1204 cm^{-1}. ^1H NMR ($CDCl_3$, 300 MHz, 25 °C): δ (ppm) 1.35 (t, 3H, *J* = 7.0 Hz, CH_3CH_2O); 4.15 (s, 1H, OCH); 4.35 (q, 2H, *J* = 7.0 Hz, CH_3CH_2O); 6.84–6.93 (m, 2H, CH_{arom}); 7.07 (t, 1H, *J* = 8.6 Hz, CH_{arom}); 8.44 (s, 1H, NH). ^{13}C NMR ($CDCl_3$, 75 MHz, 25 °C): δ (ppm) 15.1, 59.5, 61.1, 62.9, 113.1 (d, J_{CF} = 7.9 Hz), 115.3 (d, J_{CF} = 26.7 Hz), 116.4 (d, J_{CF} = 23.9 Hz), 120.1 (d, J_{CF} = 9.1 Hz), 139.1, 158.1(d, J_{CF} = 242.2 Hz), 166.8, 171.5 HRMS: exact mass calculated for ($C_{12}H_{10}FNNaO_4$) requires *m/z* 274.0492, found *m/z* 274.0497. Chiral-phase HPLC analysis: [Daicel Chiralpack IB 5μ, λ=254 nm, *n*Heptane/EtOH/DEA=70/30/0.1, flow rate 1.0mL/min]: T_{major} = 5.22 min, T_{minor} = 5.97 min *er* = 61:39. $[\alpha]_D$ = -381 (*c* = 0.0221 g/cm^3 in $CHCl_3$).

(2'S,3'S)-ethyl 5-chloro-2-oxospiro[indoline-3,2'-oxirane]-3'-carboxylate ***12o***

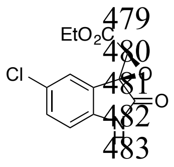

Following the above general procedure, *trans* diastereoisomer **12o** was obtained as a white solid in 33% yield after purification by flash chromatography on silica gel (nHexane/EtOAc=1/1). IR ($CHCl_3$): $\tilde{\nu}$ = 3433, 3213, 3021, 1764, 1755, 1602, 1441, 1240, 1228, 1213 cm^{-1}. ^1H NMR ($CDCl_3$, 300 MHz, 25 °C): δ (ppm) 1.32 (t, 3H, *J* = 7.1 Hz, CH_3CH_2O); 4.19 (s, 1H, OCH); 4.26-4.36 (m, 2H, CH_3CH_2O); 6.88 (m, 1H, CH_{arom}); 7.06 (t, 1H, *J* = 8.4 Hz, CH_{arom}); 7.24 (m, 1H, CH_{arom}); 8.66 (s, 1H, NH). ^{13}C NMR ($CDCl_3$, 75 MHz, 25 °C): δ (ppm) 14.3, 60.0, 60.5, 62.7, 112.1, 123.3,

125.9, 129.0, 131.3, 140.3, 165.4, 171.8. HRMS: exact mass calculated for ($C_{12}H_{10}ClNNaO_4$) requires *m/z* 290.0196, found *m/z* 290.0198. Chiral-phase HPLC analysis: [Daicel Chiralpack IC 5μ, λ=254 nm, nHexane/EtOH=70/30, flow rate 1.0mL/min]: T_{major} = 5.15 min, T_{minor} = 4.66 min *er* = 73:27. $[\alpha]_D$ = -43 (c = 0.0108 g/cm^3 in $CHCl_3$).

(2′R,3′S)-ethyl 5-chloro-2-oxospiro[indoline-3,2′-oxirane]-3′-carboxylate ***13o***

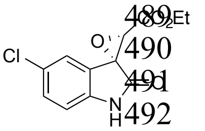

Following the above general procedure, *cis* diastereoisomer ***13o*** was obtained as a white solid in 50% yield after purification by flash chromatography on silica gel (nHexane/EtOAc=1/1). IR ($CHCl_3$): $\tilde{\nu}$ = 3433, 3265, 3024, 1761, 1733, 1633, 1481, 1277, 1200 cm^{-1}. ^{1}H NMR ($CDCl_3$, 300 MHz, 25 °C): δ (ppm) 1.34 (t, 3H, *J* = 7.2 Hz, CH_3CH_2O); 4.16 (s, 1H, OCH); 4.34 (q, 2H, *J* = 7.2 Hz, CH_3CH_2O); 6.92 (d, 1H, *J* = 8.3 Hz, CH_{arom}); 7.08 (s, 1H, CH_{arom}); 7.33 (d, 1H, *J* = 8.3 Hz, CH_{arom}); 8.65 (s, 1H, NH). ^{13}C NMR ($CDCl_3$, 75 MHz, 25 °C): δ (ppm) 14.2, 60.1, 60.5, 62.4, 112.4, 123.3, 128.9, 131.3, 131.4, 140.9, 164.2, 170.7. HRMS: exact mass calculated for ($C_{12}H_{10}ClNNaO_4$) requires *m/z* 290.0196, found *m/z* 290.0198 Chiral-phase HPLC analysis: [Daicel Chiralpack IC 5μ, λ=254 nm, *n*Heptane/EtOH/DEA=70/30/0.1, flow rate 1.0mL/min]: T_{major} = 5.30 min, T_{minor} = 6.13 min *er* = 59:41. $[\alpha]_D$ = -29 (*c* = 0.0164 g/cm^3 in $CHCl_3$).

(2′S,3′S)-ethyl 5-iodo-2-oxospiro[indoline-3,2′-oxirane]-3′-carboxylate ***12p***

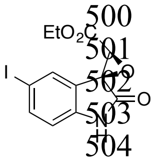

Following the above general procedure, *trans* diastereoisomer **12p** was obtained as a white solid in 71% yield after purification by flash chromatography on silica gel (nHexane/EtOAc=1/1). IR ($CHCl_3$): $\tilde{\nu}$ = 1765, 1760, 1602, 1441, 1240, 1228 cm^{-1}. ^{1}H NMR ($CDCl_3$, 300 MHz, 25 °C): δ (ppm) 1.34 (t, 3H, *J* = 7.0 Hz, CH_3CH_2O); 4.17-4.40 (m, 3H, CHO, CH_3CH_2O); 6.76 (d, 1H, *J* = 8.3 Hz, CH_{arom}); 7.67 (d, 1H, *J* = 8.3 Hz, CH_{arom}); 7.76 (s, 1H, CH_{arom}), 8.73 (bs, 1H, NH). ^{13}C NMR ($CDCl_3$, 75 MHz, 25 °C): δ (ppm) 14.4, 59.8, 60.1, 62.7, 85.6, 113.0, 122.0, 134.2, 140.1, 142.4, 165.4, 171.2. HRMS: exact mass calculated for ($C_{12}H_{10}INNaO_4$) requires *m/z* 381.9552, found *m/z* 381.9555. Chiral-phase HPLC analysis: [Daicel Chiralpack IC 5μ, λ=254 nm, nHexane/EtOH=90/10, flow rate 1.0mL/min: T_{major} = 11.56 min, T_{minor} = 9.81 min *ee* = 68:32. $[\alpha]_D$ = -23 (c = 0.0120 g/cm^3 in $CHCl_3$).

(2′R,3′S)-ethyl 5-iodo-2-oxospiro[indoline-3,2′-oxirane]-3′-carboxylate ***13p***

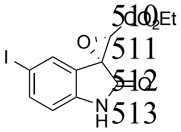

Following the above general procedure, *cis* diastereoisomer **13p** was obtained as a white solid in 20% yield after purification by flash chromatography on silica gel (*n*Hexane/EtOAc=1/1). IR ($CHCl_3$): $\tilde{\nu}$ = 3025, 3017, 3008, 1753, 1736, 1611, 1486, 1465, 1430, 1340 cm^{-1}. ^{1}H NMR ($CDCl_3$, 300 MHz, 25 °C): δ (ppm) 1.35 (t, 3H, *J* = 7.1 Hz, CH_3CH_2O); 4.18 (s, 1H, OCH); 4.25-4.43 (m, 2H, CH_3CH_2O); 6.77 (d, 1H, *J* = 8.2 Hz, CH_{arom}); 7.67 (d, 1H, *J* = 8.2 Hz, CH_{arom}); 7.76 (s, 1H, CH_{arom}); 8.81 (s, 1H, NH). ^{13}C NMR ($CDCl_3$, 75 MHz, 25 °C): δ (ppm) 14.2, 60.1, 60.5, 62.4, 112.4, 123.3, 128.9, 131.3, 131.4, 140.9, 164.2, 170.7. HRMS: exact mass calculated for ($C_{12}H_{10}INNaO_4$) requires *m/z* 381.9552, found *m/z* 381.9555. Chiral-phase HPLC analysis: [Daicel Chiralpack IB 5μ, λ=254 nm, *n*Heptane/EtOH/DEA=70/30/0.1, flow rate 1.0mL/min: T_{major} = 5.46 min, T_{minor} = 6.43 min *er* = 59:41. $[\alpha]_D$ = -2 (*c* = 0.0101 g/cm^3 in $CHCl_3$).

(2′S,3′S)-ethyl 2-oxo-5-(trifluoromethoxy)spiro[indoline-3,2′-oxirane]-3′-carboxylate ***12q***

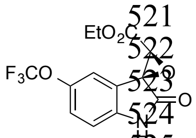

Following the above general procedure, *trans* diastereoisomer **12q** was obtained as a white solid in 29% yield after purification by flash chromatography on silica gel (nHexane/EtOAc=7/3). IR ($CHCl_3$): $\tilde{\nu}$ = 3433, 3210, 3028, 1764, 1724, 1630, 1478, 1371, 1234, 1197 cm^{-1}. ^{1}H NMR ($CDCl_3$, 300 MHz, 25 °C): δ (ppm) 1.31 (t, 3H, *J* = 7.0 Hz, CH_3CH_2O); 4.20 (s, 1H, OCH); 4.31 (q, 2H, *J* = 7.0 Hz, CH_3CH_2O); 6.97 (d, 1H, *J* = 8.4 Hz, CH_{arom}); 7.22-7.26 (m, 1H, CH_{arom}); 7.41 (s, 1H, CH_{arom}); 8.41 (bs, 1H, NH). ^{13}C NMR ($CDCl_3$, 75 MHz, 25 °C): δ (ppm) 14.2, 60.1, 60.0, 62.7, 111.6, 119.6, 120.6 (q, J_{CF} = 256.0 Hz), 121.3, 124.6, 141.3, 145.1, 165.3, 171.7. HRMS: exact mass calculated for ($C_{13}H_{10}F_3NNaO_5$) requires *m/z* 340.0409, found *m/z* 340.0410. Chiral-phase HPLC analysis: [Daicel Chiralpack IC 5μ, λ=254 nm, nHexane/EtOH=95/5, flow rate 1.0mL/min: T_{major} = 12.04 min, T_{minor} = 9.15 min *er* = 87:13. $[\alpha]_D$ = -56.53 (c = 0.0168 g/cm^3 in $CHCl_3$).

(2′R,3′S)-ethyl 2-oxo-5-(trifluoromethoxy)spiro[indoline-3,2′-oxirane]-3′-carboxylate ***13q***

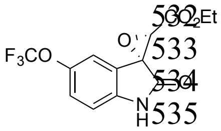

Following the above general procedure, *cis* diastereoisomer **13q** was obtained as a white solid in 47% yield after purification by flash chromatography on silica gel (nHexane/EtOAc=7/3). IR ($CHCl_3$): $\tilde{\nu}$ = 3433, 3207, 3015, 1764, 1724, 1636, 1584, 1487, 1264, 1234 cm^{-1}. ^1H NMR ($CDCl_3$, 300 MHz, 25 °C): δ (ppm) 1.35 (t, 3H, *J* = 7.0 Hz, CH_3CH_2O); 4.19 (s, 1H, OCH); 4.35 (q, 2H, J = 7.0 Hz, CH_3CH_2O); 6.99-7.01 (m, 2H, CH_{arom}); 7.23 (s, 1H, CH_{arom}); 8.57 (bs, 1H, NH). ^{13}C NMR ($CDCl_3$, 75 MHz, 25 °C): δ (ppm) 14.2, 60.3, 60.5, 62.5, 112.4, 116.6, 120.6 (q, J_{CF} = 257.3 Hz), 122.3, 124.7, 141.3, 145.1, 164.3, 171.3. HRMS: exact mass calculated for ($C_{13}H_{10}F_3NNaO_5$) requires *m/z* 340.0409, found *m/z* 340.0410. Chiral-phase HPLC analysis: [Daicel Chiralpack IB 5μ, λ=254 nm, *n*Heptane/EtOH/DEA=70/30/0.1, flow rate 1.0mL/min+: T_{major} = 4.44 min, T_{minor} = 5.31 min *er* = 66:34. $[\alpha]_D$ = -20.7 (*c* = 0.0133 g/cm^3 in $CHCl_3$).

(2′S,3′S)-ethyl 7-fluoro-2-oxospiro[indoline-3,2′-oxirane]-3′-carboxylate ***12r***

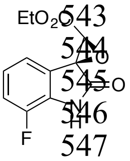

Following the above general procedure, *trans* diastereoisomer **12r** was obtained as a white solid in 21% yield after purification by flash chromatography on silica gel (nHexane/EtOAc=8/2). IR ($CHCl_3$): $\tilde{\nu}$ = 3433, 3031, 2976, 2930, 1752, 1737, 1639, 1493, 1234, 1228 cm^{-1}. ^1H NMR ($CDCl_3$, 300 MHz, 25 °C): δ (ppm) 1.30 (t, 3H, *J* = 7.0 Hz, CH_3CH_2O); 4.20 (s, 1H, OCH); 4.25-4.30 (m, 2H, CH_3CH_2O); 7.01-7.05 (m, 1H, CH_{arom}); 7.14 (t, 1H, *J* = 9.2 Hz, CH_{arom}); 7.28 (d, 1H, *J* = 9.2 Hz, CH_{arom}); 7.84 (s, 1H, NH). ^{13}C NMR ($CDCl_3$, 75 MHz, 25 °C): δ (ppm) 14.3, 60.0, 60.1, 62.5, 118.4 (d, J_{CF} = 17 Hz), 121.1 (d, J_{CF} = 3.6 Hz), 122.2, 124.0 (d, J_{CF} = 5.9 Hz), 129.9, 147.4 (d, J_{CF} = 244.7 Hz), 165.3, 170.5. HRMS: exact mass calculated for ($C_{12}H_{10}FNNaO_4$) requires *m/z* 274.0492, found *m/z* 274.0493. Chiral-phase HPLC analysis: [Daicel Chiralpack IC 5μ, λ=254 nm, nHexane/EtOH=8/2, flow rate 1.0mL/min: T_{major} = 7.88 min, T_{minor} = 8.60 min *er* = 92:8. $[\alpha]_D$ = -63.2 (c = 0.0150 g/cm^3 in $CHCl_3$).

(2′R,3′S)-ethyl 7-fluoro-2-oxospiro[indoline-3,2′-oxirane]-3′-carboxylate ***13r***

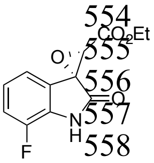

Following the above general procedure, *cis* diastereoisomer **13r** was obtained as a white solid in 72% yield after purification by flash chromatography on silica gel (nHexane/EtOAc=8/2). IR ($CHCl_3$): $\tilde{\nu}$ = 3433, 3201, 3056, 2988, 1773, 1761, 1605, 1328, 1252 cm^{-1}. ^1H NMR ($CDCl_3$, 300 MHz, 25 °C): δ (ppm) 1.36 (t, 3H, *J* = 7.1 Hz, CH_3CH_2O); 4.18 (s, 1H, OCH); 4.36 (q, 2H, *J* = 7.1 Hz, CH_3CH_2O); 6.92 (d, 1H, *J* = 7.4 Hz, CH_{arom}); 7.01-7.17 (m, 2H, CH_{arom}); 8.42 (s, 1H, NH). ^{13}C NMR ($CDCl_3$, 75 MHz, 25 °C): δ (ppm) 14.1, 60.3, 60.5, 62.4, 118.4 (d, J_{CF} = 8.6 Hz), 118.6 (d, J_{CF} = 5.1 Hz), 124.0, 124.2 (d, *JCF* = 3.5 Hz), 129.8 (d, J_{CF} = 13.2 Hz), 147.5 (d, *JCF* = 246.1 Hz), 164.5, 170.6. HRMS: exact mass calculated for ($C_{12}H_{10}FNNaO_4$) requires *m/z* 274.0492, found *m/z* 274.0493. Chiral-phase HPLC analysis: [Daicel Chiralpack IB 5μ, λ=254 nm, nHeptane/EtOH/DEA=70/30/0.1, flow rate 1.0 mL/min: T_{major} = 5.08 min, T_{minor} = 5.78 min *er* = 70:30. $[\alpha]_D$ = -44.4 (c = 0.0291 g/cm^3 in $CHCl_3$).

(2′S,3′S)-ethyl 7-chloro-2-oxospiro[indoline-3,2′-oxirane]-3′-carboxylate ***12s***

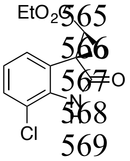

Following the above general procedure, *trans* diastereoisomer **12s** was obtained as a white solid in 35% yield after purification by flash chromatography on silica gel (nHexane/EtOAc=7/3). IR (CHCl3): $\tilde{\nu}$ = 3420, 3177, 3009, 1764, 1749, 1624, 1478, 1316, 1234, 1189 cm^{-1}. ^1H NMR ($CDCl_3$, 300 MHz, 25 °C): δ (ppm) 1.29 (t, 3H, *J* = 6.8 Hz, CH_3CH_2O); 4.20-4.31 (m, 2H, CH_3CH_2O, OCH); 7.01 (t, 1H, *J* = 8.1 Hz, CH_{arom}); 7.36 (t, 2H, *J* = 8.1 Hz, CH_{arom}); 8.14 (s, 1H, NH). ^{13}C NMR ($CDCl_3$, 75 MHz, 25 °C): δ (ppm) 14.2, 60.1, 60.7, 62.5, 116.3, 121.3, 123.6, 124.1, 131.1, 140.4, 165.3, 171.2. HRMS: exact mass calculated for ($C_{12}H_{10}ClNNaO_4$) requires *m/z* 290.0196, found *m/z* 290.0197. Chiral-phase HPLC analysis: [Daicel Chiralpack IC 5μ, λ=254 nm, nHexane/EtOH=8/2, flow rate 1.0mL/min: T_{major} = 7.72 min, T_{minor} = 10.00 min *er* = 88:12. $[\alpha]_D$ = -186.6 (c = 0.0220 g/cm^3 in $CHCl_3$).

(2′R,3′S)-ethyl 7-chloro-2-oxospiro[indoline-3,2′-oxirane]-3′-carboxylate ***13s***

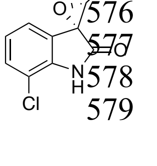

Following the above general procedure, *cis* diastereoisomer **13s** was obtained as a white solid in 63% yield after purification by flash chromatography on silica gel (nHexane/EtOAc=7/3). IR ($CHCl_3$): $\tilde{\nu}$ = 3426, 3201, 3003, 1764, 1736, 1627, 1478, 1328, 1237, 1200 cm^{-1}. ^1H NMR ($CDCl_3$, 300 MHz, 25°C): δ (ppm) 1.35 (t, 3H, *J* = 7.1 Hz, CH_3CH_2O); 4.16 (s, 1H, OCH); 4.38 (q, 2H, *J* = 7.1 Hz, CH_3CH_2O); 7.03-7.05 (m, 2H, CH_{arom}); 7.35 (d, 1H, *J* = 7.4 Hz, CH_{arom}); 8.02 (s, 1H, NH). ^{13}C NMR

($CDCl_3$, 75 MHz, 25 °C): δ (ppm) 14.2, 60.5, 60.6, 62.4, 116.4, 121.0, 123.1, 124.1, 131.2, 140.0, 164.3, 170.2. HRMS: exact mass calculated for ($C_{12}H_{10}ClNNaO_4$) requires *m*/*z* 290.0196, found *m*/*z* 290.0197. Chiralphase HPLC analysis: [Daicel Chiralpack IB 5μ, λ=254 nm, nHeptane/EtOH/DEA=70/30/0.1, flow rate 1.0mL/min: T_{major} = 5.24 min, T_{minor} = 5.67 min *er*= 62:38. $[\alpha]_D$ = -27.9 (c = 0.0168 g/cm^3 in $CHCl_3$).

*(2′S,3′S)-ethyl 7-bromo-2-oxospiro[indoline-3,2′-oxirane]-3′-carboxylate **12t***

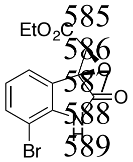

Following the above general procedure, *trans* diastereoisomer **12t** was obtained as a white solid in 49% yield after purification by flash chromatography on silica gel (nHexane/EtOAc=7/3). IR ($CHCl_3$): $\tilde{\nu}$ = 3414, 3210, 3006, 1742, 1730, 1621, 1444, 1316, 1234 cm^{-1}. ^{1}H NMR ($CDCl_3$, 300 MHz, 25 °C): δ (ppm) 1.29 (t, 3H, *J* = 7.1 Hz, CH_3CH_2O); 4.20 (s, 1H, OCH); 4.23-4.37 (m, 2H, CH_3CH_2O); 6.96 (t, 1H, *J* = 7.9 Hz, CH_{arom}); 7.42 (d, 1H, *J* = 7.9 Hz, CH_{arom}); 7.48 (d, 1H, *J* = 7.9 Hz, CH_{arom}); 8.06 (s, 1H, NH). ^{13}C NMR ($CDCl_3$, 75 MHz, 25 °C): δ (ppm) 14.3, 60.1, 61.0, 62.6, 104.1, 121.4, 124.3, 124.5, 133.9, 141.9, 165.3, 170.5. HRMS: exact mass calculated for ($C_{12}H_{10}BrNNaO_4$) requires *m*/*z* 333.9691, found *m*/*z* 333.9693. Chiralphase HPLC analysis: [Daicel Chiralpack IC 5μ, λ=254 nm, nHexane/EtOH=7/3, flow rate 1.0mL/min: T_{major} = 6.14 min, T_{minor} = 7.18 min *er* = 92:8. $[\alpha]_D$ = -15.85 (c = 0.0132 g/cm^3 in $CHCl_3$).

*(2′S,3′R)-ethyl 7-bromo-2-oxospiro[indoline-3,2′-oxirane]-3′-carboxylate **13t***

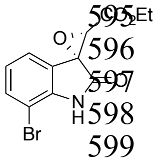

Following the above general procedure, *cis* diastereoisomer **13t** was obtained as a white solid in 38% yield after purification by flash chromatography on silica gel (nHexane/EtOAc=7/3). IR ($CHCl_3$): $\tilde{\nu}$ = 3414, 3210, 3006, 1742, 1730, 1621, 1444, 1316, 1234 cm^{-1}. ^{1}H NMR ($CDCl_3$, 300 MHz, 25 °C): δ (ppm) 1.35 (t, 3H, *J* = 6.9 Hz, CH_3CH_2O); 4.16 (s, 1H, OCH); 4.36 (q, 2H, *J* = 6.9 Hz, CH_3CH_2O); 6.97-7.07 (m, 2H, CH_{arom}); 7.49 (d, 1H, *J* = 7.8 Hz, CH_{arom}); 7.66 (s, 1H, NH). ^{13}C NMR ($CDCl_3$, 75 MHz, 25 °C): δ (ppm) 14.2, 60.7, 60.9, 62.4, 104.2, 121.7, 123.2, 124.4, 134.0, 141.6, 164.2, 169.5. HRMS: exact mass calculated for ($C_{12}H_{10}BrNNaO_4$) requires *m*/*z* 333.9691, found *m*/*z* 333.9693. Chiral-phase HPLC analysis: [Daicel Chiralpack IC 5μ, λ=254 nm, nHexane/EtOH=8/2, flow rate 1.0mL/min: T_{major} = 13.98 min, T_{minor} = 12.80 min *er* = 65:35. $[\alpha]_D$ = -32.92 (c = 0.0120 g/cm^3 in $CHCl_3$).

*(2′S,3′S)-ethyl 5,7-dichloro-2-oxospiro[indoline-3,2′-oxirane]-3′-carboxylate **12u***

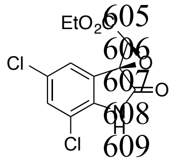

Following the above general procedure, *trans* diastereoisomer **12u** was obtained as a white solid in 27% yield after purification by flash chromatography on silica gel (nHexane/EtOAc=7/3). IR ($CHCl_3$): $\tilde{\nu}$ = 3423, 3031, 3009, 1757, 1743, 1463, 1323, 1290 cm^{-1}. ^{1}H NMR ($CDCl_3$, 300 MHz, 25 °C): δ (ppm) 1.32 (t, *J* = 7.0 Hz, 3H, CH_3CH_2O); 4.18-4.36 (m, 3H, CH_3CH_2O, OCH); 7.38 (s, 1H, CH_{arom}); 7.42 (s, 1H, CH_{arom}); 8.09 (s, 1H, NH). ^{13}C NMR ($CDCl_3$, 75 MHz, 25 °C): δ (ppm) 14.3, 60.1, 60.4, 62.9, 116.6, 122.4, 124.4, 129.3, 130.8, 138.9, 165.1, 170.1. HRMS: exact mass calculated for ($C_{12}H_9Cl_2NNaO_4$) requires *m*/*z* 323.9806, found *m*/*z* 323.9808. Chiral-phase HPLC analysis: [Daicel Chiralpack IC 5μ, λ=254 nm, nHexane/EtOH=7/3, flow rate 1.0mL/min: T_{major} = 5.52 min, T_{minor} = 6.34 min *er* = 83:17. $[\alpha]_D$ = +137 (c = 0.0090 g/cm^3 in $CHCl_3$).

*(2′S,3′R)-ethyl 5,7-dichloro-2-oxospiro[indoline-3,2′-oxirane]-3′-carboxylate **13u***

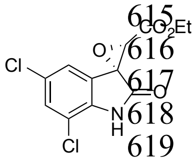

Following the above general procedure, *cis* diastereoisomer **13u** was obtained as a white solid in 51% yield after purification by flash chromatography on silica gel (nHexane/EtOAc=7/3). IR ($CHCl_3$): $\tilde{\nu}$ = 3423, 3031, 3003, 1740, 1633, 1469, 1315 cm^{-1}. ^{1}H NMR ($CDCl_3$, 300 MHz, 25 °C): δ (ppm) 1.35 (t, *J* = 7.2 Hz, 3H, CH_3CH_2O); 4.16 (s, OCH); 4.35(q, *J* = 6.6 Hz, 2H, CH_3CH_2O); 7.01 (s, 1H, CH_{arom}); 7.37 (s, 1H, CH_{arom}); 8.31 (s, 1H, NH). ^{13}C NMR ($CDCl_3$, 75 MHz, 25 °C): δ (ppm) 14.2, 60.1, 60.6, 62.6, 116.8, 121.8, 124.4, 129.2, 130.9, 138.6, 163.8, 169.3. HRMS: exact mass calculated for ($C_{12}H_9Cl_2NNaO_4$) requires *m*/*z* 323.9806, found *m*/*z* 323.9808. Chiralphase HPLC analysis: [Daicel Chiralpack IC 5μ, λ=254 nm, nHexane/EtOH=9/1, flow rate 1.0mL/min: T_{major} = 20.84 min, T_{minor} = 19.81 min *er* = 86:14. $[\alpha]_D$ = +10 (c = 0.0100 g/cm^3 in $CHCl_3$).

(2R,3'R)-1-(tert-butyl) 3'-ethyl -2-oxospiro[indoline-3,2'-oxirane]-1,3'-dicarboxylate ***12v***

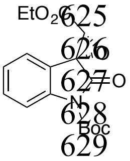

Following the above general procedure, *trans* diastereoisomer **12v** was obtained as a white solid in 60% yield after purification by flash chromatography on silica gel (nHexane/EtOAc=7/3). IR ($CHCl_3$): $\tilde{\nu}$ = 3035, 307, 2984, 1736, 1618, 1603,1495, 1473, 1376, 1347 cm^{-1}. ^1H NMR ($CDCl_3$, 300 MHz, 25 °C): δ (ppm) 1.23 (t, *J* = 5.9 Hz, 3H, *CH*$_3$CH$_2$O), 1.48 (s, 9H), 4.22 (s, 2H, CH$_3$*CH*$_2$O), 4.59 - 4.45 (m, 1H), 7.22 (dtd, *J* = 26.3, 7.4, 2.0 Hz, 1H, CH_{arom}), 7.38 (dd, *J* = 7.3, 2.2 Hz, 2H, CH_{arom}), 7.89 (dd, *J* = 7.4, 2.1 Hz, 1H, CH_{arom}). ^{13}C NMR ($CDCl_3$, 75 MHz, 25 °C): δ (ppm) 14.0, 27.8, 60.5, 61.1, 69.2, 83.4, 114.4, 115.3, 125.2, 125.8, 129.3, 131.3, 150.5, 166.4, 170.8. HRMS: exact mass calculated for ($C_{17}H_{19}NNaO_6$) requires *m/z* 356,1110, found *m/z* 356,1112. Chiralphase HPLC analysis: [Daicel Chiralpack IC 5μ, λ=254 nm, nHexane/EtOH=9/1, flow rate 1.0mL/min: T_{major} = 6.80 min, T_{minor} = 5.75 min *er* = 96:4. $[\alpha]_D$ = +27 (c = 0.0103 g/cm^3 in $CHCl_3$).

(2S,3'R)-1-(tert-butyl) 3'-ethyl -2-oxospiro[indoline-3,2'-oxirane]-1,3'-dicarboxylate ***13v***

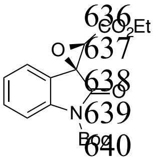

Following the above general procedure, *cis* diastereoisomer **13v** was obtained as a white solid in 60% yield after purification by flash chromatography on silica gel (nHexane/EtOAc=7/3). IR ($CHCl_3$): $\tilde{\nu}$ = 3034, 3076, 2984, 1736, 1618, 1608,1498, 1473, 1376, 1347cm^{-1}. ^1H NMR ($CDCl_3$, 300 MHz, 25 °C): δ (ppm) 1.37 (t, *J* = 5.9 Hz, 3H, *CH*$_3$CH$_2$O), 1.64 (s, 9H), 4.17 (s, 2H, CH$_3$*CH*$_2$O), 4.36 (q, *J* = 7,2 Hz, 2H, CH$_3$CH$_2$O), 6,91 - 7,10 (m, 2H, CH_{arom}); 7,42 - 7,46(m, 2H, CH_{arom}) ppm. ^{13}C NMR ($CDCl_3$, 75 MHz, 25 °C): δ (ppm) 14.2, 28.0, 60.3, 61.5, 68.9, 83.6, 114.4, 115.3, 125.8, 126.4, 127.3, 131.3, 150.5, 166.4, 170.8. HRMS: exact mass calculated for ($C_{17}H_{19}NNaO_6$) requires *m/z* 356,1110, found *m/z* 356,1116. Chiralphase HPLC analysis: [Daicel Chiralpack IC 5μ, λ=254 nm, nHexane/EtOH=9/1, flow rate 1.0mL/min: T_{major} = 15.27 min, T_{minor} = 14.48 min *er* = 64:36. $[\alpha]_D$ = -3 (c = 0.0112 g/cm^3 in $CHCl_3$).

4 Conformational analysis

Conformational analysis was carried out by arbitrarily fixing *S* configuration on 2' carbon and performing a molecular mechanics (MM) conformational search on the two possible diastereomers of *trans*-**12h** and *cis*-**13h** with (2'*S*,3'*S*) and (2'*S*,3'*R*) absolute configuration (AC). The searches provided 82 conformers for both diastereomer. All conformers were fully optimized at DFT level of theory with polarizable continuum model (PCM). In Figure SM-1 the most populated conformers (90% of overall population) considered in chiroptical properties calculations are reported.

It is important to notice (vide infra) that the relative conformers populations, calculated on the basis of free energies, slightly changes in the three distributions treated with different PCM solvent models. In any case, in the scope of AC assignment and considering the similar behavior of single calculated conformers, the overall property (ECD, ORD and VCD) is not affected.

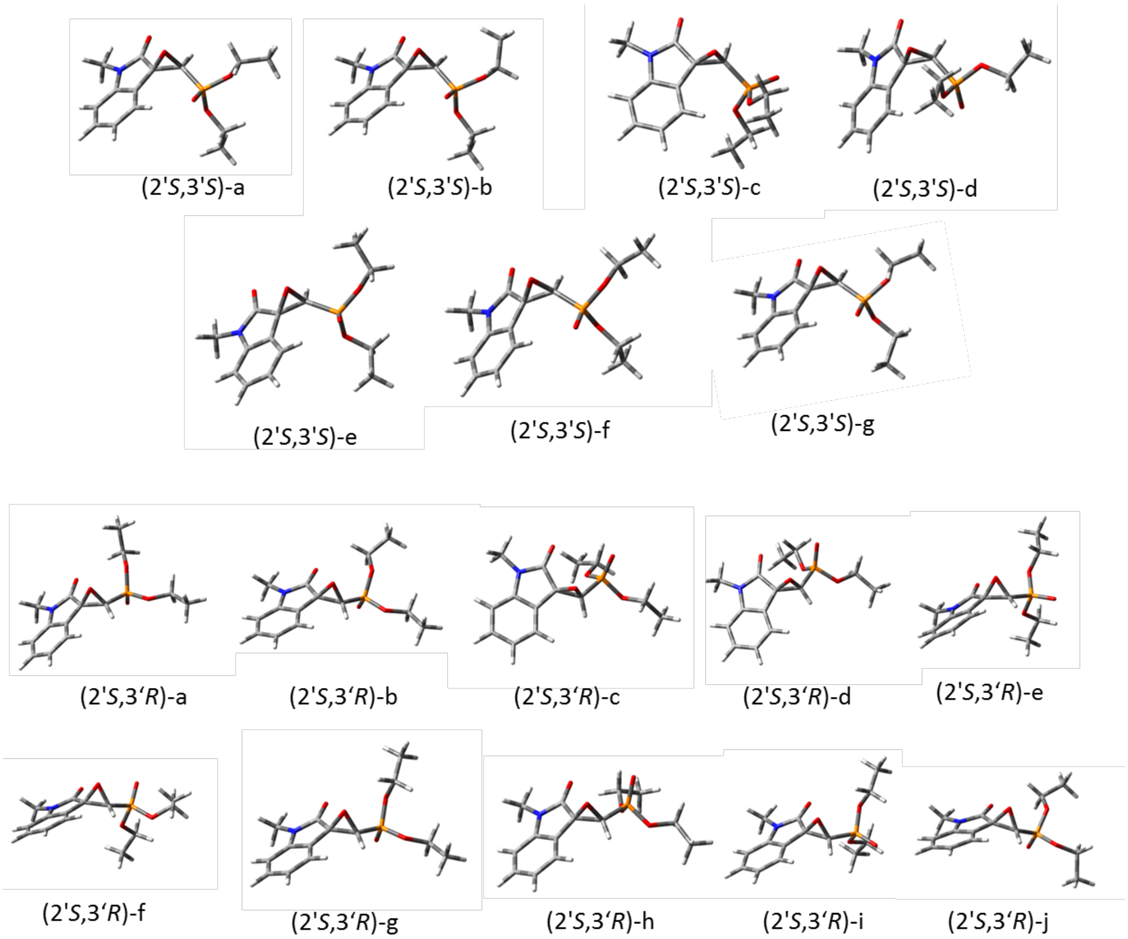

Figure SM-1. Most populated conformers involved in ECD, ORD, VCD calculations for both possible diastereomers (2'*S*,3'*S*), top, and (2'*S*,3'*R*), lower.

4.1 Calculation of ECD/UV spectra

Optimization and frequencies calculation at B3LYP/TZVP/PCM(CH_3CN) provided five most populated conformations for (2'*S*,3'*S*) and seven for (2'*S*,3'*R*) diastereomers covering 90% of overall population. For all provided conformers ECD/UV spectra were calculated at CAM-B3LYP/TZVP/PCM(CH_3CN) level (Table SM-1).

Table SM-1. Conformation analysis of the (2'*S*,3'*S*) and (2'*S*,3'*R*) diastereomers of *trans*-**12h** and *cis*-**13h**. Population factors (%pop) are calculated according to ΔG (in kcal/mol) at B3LYP/TZVP/PCM(CH_3CN) level.

CONFORMERS	ΔG	% pop	CONFORMERS	ΔG	% pop
(2'S,3'S)-a	0.00	77.0	(2'S,3'R)-a	0.00	22.0
(2'S,3'S)-b	1.20	10.1	(2'S,3'R)-b	0.13	17.6
(2'S,3'S)-c	1.48	6.3	(2'S,3'R)-c	0.24	14.7
(2'S,3'S)-d	1.72	4.2	(2'S,3'R)-d	0.43	10.9
(2'S,3'S)-e	2.06	2.4	(2'S,3'R)-e	0.42	10.7
			(2'S,3'R)-f	0.64	7.5
			(2'S,3'R)-g	0.71	6.6

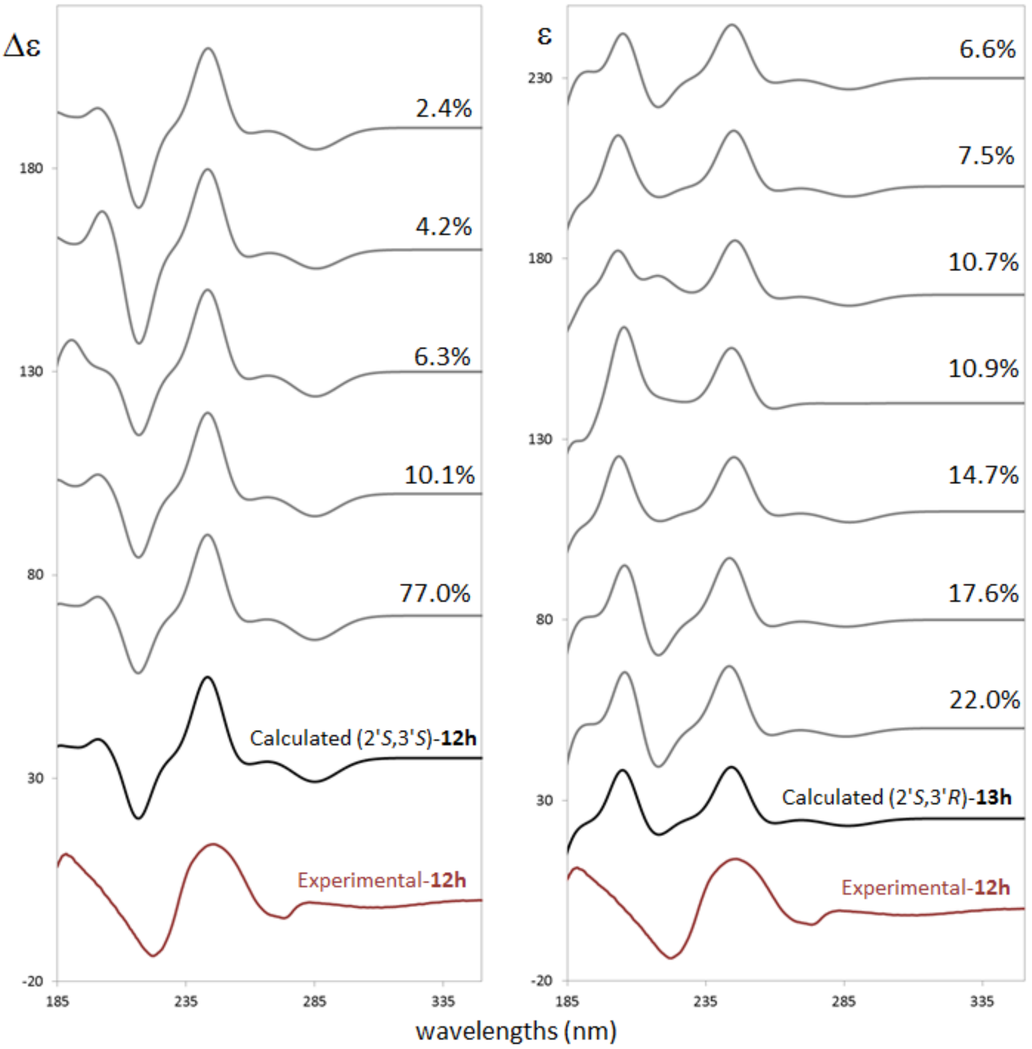

Figure SM-2. Comparison of experimental and calculated ECD spectra for the different AC of compound *trans*-**12h** and *cis*-**13h** for the statistical average and for single conformer (CAM-B3LYP/TZVP/PCM(CH_3CN)).

4.2 Calculation of ORD spectra

Optimization and frequencies calculation at B3LYP/TZVP/PCM($CHCl_3$) provided five most populated conformations for (2'*S*,3'*S*) and six for (2'*S*,3'*R*) diastereomers covering 90% of overall population. The experimental ORD was measured in $CHCl_3$ at concentration of 0.35 g/100 mL.

Table SM-2. Conformation analysis of the (2'*S*,3'*S*) and (2'*S*,3'*R*) diastereomers of *trans*-**12h** and *cis*-**13h**. Population factors (%pop) are calculated according to ΔG (in kcal/mol) at B3LYP/TZVP/PCM($CHCl_3$) level.

CONFORMERS	ΔG	% pop	589	546	435	405
(2'S,3'S)-b	0.00	47.4	-67	-82	-162	-211
(2'S,3'S)-a	0.08	41.5	-26	-33	-84	-122
(2'S,3'S)-c	1.29	5.4	-169	-204	-381	-481
(2'S,3'S)-e	1.58	3.3	-82	-100	-196	-255
(2'S,3'S)-f	1.76	2.4	7	6	-18	-44
		**Average**	**-54**	**-67**	**-139**	**-186**
		**Experimental**	**-20**	**-27**	**-74**	**-110**

CONFORMERS	ΔG	% pop	589	546	435	405
(2'S,3'R)-a	0.00	34.9	68	80	127	144
(2'S,3'R)-b	0.11	29.2	80	95	160	188
(2'S,3'R)-e	0.22	24.2	199	236	397	467
(2'S,3'R)-g	1.05	6.0	48	55	76	75
(2'S,3'R)-h	1.44	3.1	173	208	368	446
(2'S,3'R)-c	1.52	2.7	9	10	10	5
		**Average**	**103**	**122**	**203**	**236**
		**Experimental**	**-20**	**-27**	**-74**	**-110**

4.3 Calculation of VCD/IR spectra

Optimization and frequencies calculation at B3LYP/TZVP/PCM(CCl_4) provided five most populated conformations for (2'*S*,3'*S*) and (2'*S*,3'*R*) diastereomers covering 90% of overall population. All provided conformers VCD/IR spectra were calculated at the same level.

Table SM-3. Conformation analysis of the (2'*S*,3'*S*) and (2'*S*,3'*R*) diastereomers of *trans*-**12h** and *cis*-**13h**. Population factors (%pop) are calculated according to ΔG (in kcal/mol) at B3LYP/TZVP/PCM(CCl_4) level.

CONFORMERS	ΔG	% pop	CONFORMERS	ΔG	% pop
(2'S,3'S)-a	0	62.5	(2'S,3'R)-b	0	48.1
(2'S,3'S)-b	0.63	22.1	(2'S,3'R)-a	0.58	17.9
(2'S,3'S)-g	1.16	8.8	(2'S,3'R)-i	0.75	13.6
(2'S,3'S)-c	1.73	3.4	(2'S,3'R)-j	0.84	11.6
(2'S,3'S)-e	1.73	3.3	(2'S,3'R)-k	1.01	8.8

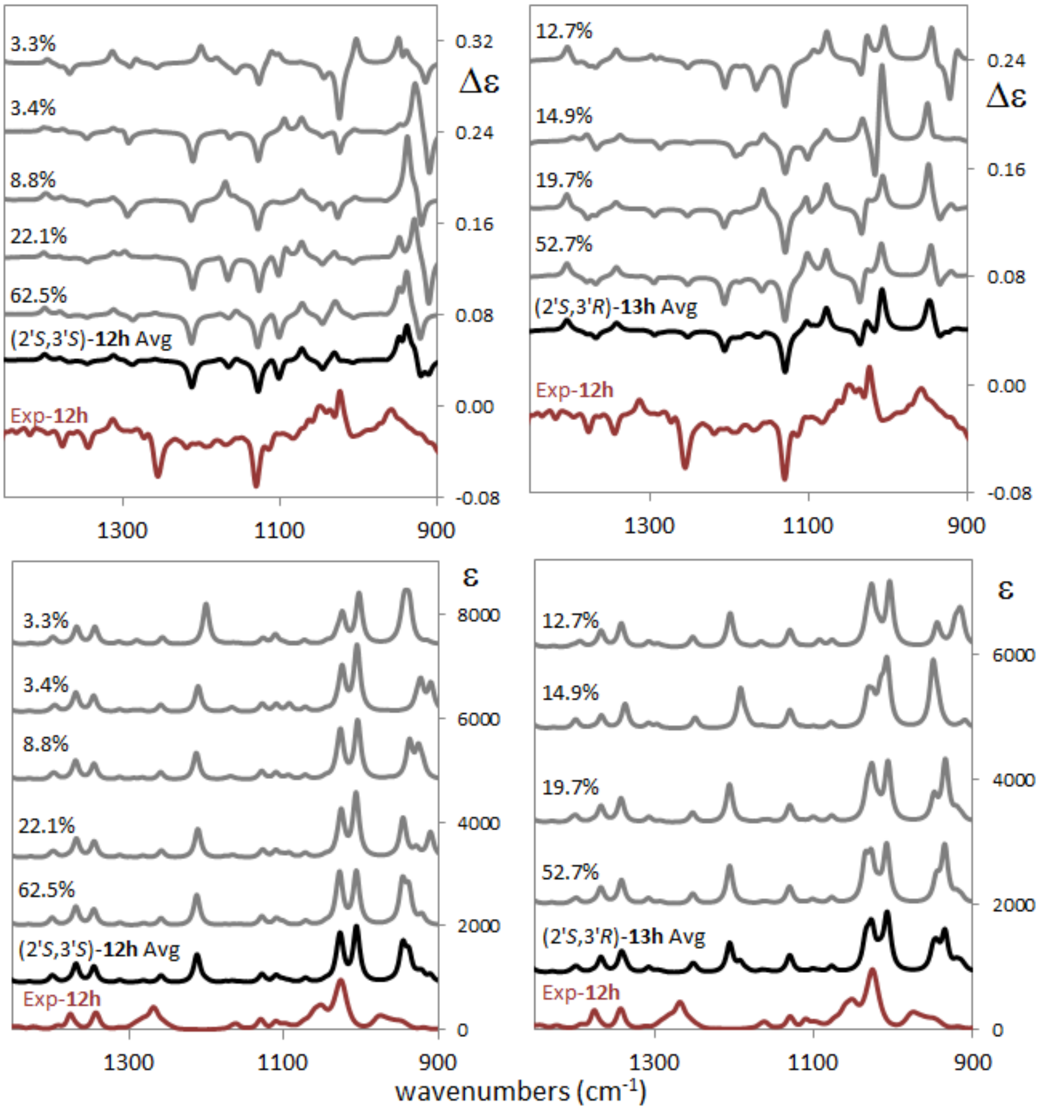

Figure SM-3. Comparison of experimental and calculated VCD (top) and IR (lower) spectra for the different AC of compound *trans*-**12h** and *cis*-**13h** for the statistical average and for single conformer (B3LYP/TZVP/PCM(CCl_4)).

References

1. Gao, Y.N.; Xu, Q.; Wei, Y.; Shi, M. Exploration of a new zwitterion: Phosphine-catalyzed 2+1+2 cycloaddition reaction. *Adv. Synth. Catal.* **2017**, *359*, 1663-1671.
2. Cao, Y.M.; Jiang, X.X.; Liu, L.P.; Shen, F.F.; Zhang, F.T.; Wang, R. Enantioselective michael/cyclization reaction sequence: Scaffold-inspired synthesis of spirooxindoles with multiple stereocenters. *Angew. Chem. Int. Edit.* **2011**, *50*, 9124-9127.
3. Gasperi, T.; Loreto, M.A.; Migliorini, A.; Ventura, C. Synthesis of aziridine- and oxirane-2-phosphonates spiro-fused with oxindoles. *Eur. J. Org. Chem.* **2011**, 385-391.
4. Jiang, T.; Kuhen, K.L.; Wolff, K.; Yin, H.; Bieza, K.; Caldwell, J.; Bursulaya, B.; Wu, T.Y.H.; He, Y. Design, synthesis and biological evaluations of novel oxindoles as hiv-1 non-nucleoside reverse transcriptase inhibitors. Part I. *Bioorg. Med Chem. Lett.* **2006**, *16*, 2105-2108.
